# Genome-Scale Modeling of the Protein Secretory Machinery in Yeast

**DOI:** 10.1371/journal.pone.0063284

**Published:** 2013-05-07

**Authors:** Amir Feizi, Tobias Österlund, Dina Petranovic, Sergio Bordel, Jens Nielsen

**Affiliations:** 1 Novo Nordisk Foundation Center for Biosustainability, Chalmers University of Technology, Gothenburg, Sweden; 2 Novo Nordisk Foundation Center for Biosustainability, Technical University of Denmark, Hørsholm, Denmark; The Centre for Research and Technology, Hellas, Greece

## Abstract

The protein secretory machinery in Eukarya is involved in post-translational modification (PTMs) and sorting of the secretory and many transmembrane proteins. While the secretory machinery has been well-studied using classic reductionist approaches, a holistic view of its complex nature is lacking. Here, we present the first genome-scale model for the yeast secretory machinery which captures the knowledge generated through more than 50 years of research. The model is based on the concept of a Protein Specific Information Matrix (PSIM: characterized by seven PTMs features). An algorithm was developed which mimics secretory machinery and assigns each secretory protein to a particular secretory class that determines the set of PTMs and transport steps specific to each protein. Protein abundances were integrated with the model in order to gain system level estimation of the metabolic demands associated with the processing of each specific protein as well as a quantitative estimation of the activity of each component of the secretory machinery.

## Introduction

Compartmentalization of cellular processes is one of the main characteristics of eukaryal cells and allows for a spatial separation of different processes within the cell [1]. Along with the evolution of compartmentalization, eukaryotic cells have developed so-called the protein secretory machinery which mostly comprises the endoplasmic reticulum (ER) and the Golgi apparatus that is in charge of transporting of many secretory and transmembrane proteins as well as carrying out the post-translational modifications (PTMs) necessary for the correct functionality of each protein. Comprehensive investigation on the membrane trafficking mechanisms in eukaryal, which was initiated in the early 1980s by Schekman and colleagues, has provided extensive mechanistic information about the secretory machinery in yeast and human nerve cells [2]–[4]. Many enzymes, protein complexes, and receptors of the secretory machinery are involved in processes such as glycosylation, folding, and trafficking and in human, malfunction of these processes can result in diseases such as *Congenital Disorders of Glycosylation (CDG), Alzheimer’s*, and *Parkinson’s* [5]–[10].

Here, we developed a genome-scale network reconstruction approach to enable quantitative analysis of this complex machinery and capture its protein-specific function. *Genome-scale network reconstruction* is a comprehensive compilations of the molecular components and their mechanistic interactions involved in one or multiple cellular processes [11]. The molecular components in a genome-scale reconstruction are related to each other by functional relationships that are condensed in some form of mathematical structure [11], [12]. The mentioned interactions can be used as a source for different kinds of systemic-level analysis. The most reconstructed genome-scale networks are the so called genome-scale metabolic models (GEMs), which contain the metabolic enzymes present in the cell, linked to their associated chemical reactions [12]. The different enzymes are linked to each other by sharing products and substrates and the nature of these interactions is condensed in a stoichiometric matrix that represents a quantitative description of the system [13]. In the genome-scale network presented here, the interactions between components are also defined by the sharing of substrates (which are the proteins processed by the secretory machinery). Metabolic networks involve reactions with well-defined stoichiometry in which the substrates are small molecules whose concentrations are much higher than the concentrations of the enzymes catalyzing their transformations. For other complex cellular processes, such as transcription, translation, translocation from the cytosol to the ER, there is not any well-defined chemistry. This makes it difficult to expand the concept of genome-scale modeling to describe other cellular processes than metabolism. Accordingly, reconstruction and utilization of genome-scale networks for biological processes, is still a relatively unexplored field, while recently some successful examples have been performed [14]–[16]. The aim of this study was to build a genome-scale network for the protein secretory machinery in yeast and explore some of its potential applications. The reconstructed genome-scale network provides more detailed insights into the functions of the eukaryotes protein secretory machinery particularly in yeast.

## Results and Discussion

The genome-scale model for the secretory machinery of yeast was built using a bottom-up approach. We then used the model as scaffold to compare the secretion system of yeast and human. By using protein abundance data for yeast, we further utilized the model to estimate the metabolic demands associated to the processing of clients by the secretory machinery. Finally the specific activities of each molecular component of the machinery were calculated.

### Defining Components and Subsystems of the Secretory Machinery

**Figure 1:**
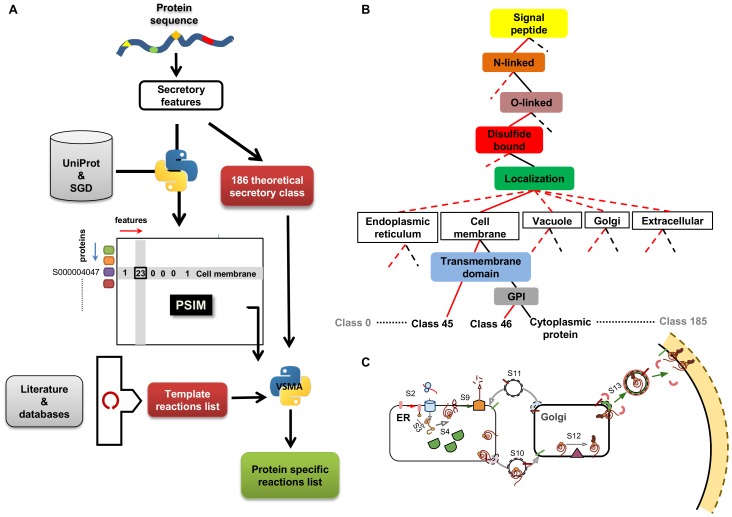
Workflow for the model reconstruction. Each protein sequence (blue string) contains motifs and signals that determine the PTMs and transport steps that the protein will undergo when it is processed by the secretory machinery (panel A, different shapes with different colors). A decision tree is used to define all the possible feature combinations (panel B). The two types of glycosylation features (*N*- and *O*-linked) are treated as two separate features. Transmembrane domain information and GPI information were used after localization to distinguish transmembrane proteins from GPI-anchored proteins (both with membrane annotation). The generated 186 theoretical classes cover all the potential secretory proteins with or without signal peptide (see Figure S1). The information about the features was extracted for the complete yeast proteome (5882 proteins) from UniProt (see Materials & Methods). The resulting information was formatted to build the Protein Specific Information Matrix (PSIM) consisting of *m* rows and *n* columns, where *m* is the number of proteins and *n* is the number of features (Panel A). Formulation of the secretory pathway model was done based on a comprehensive literature and database survey (Panel A, see Table S2 for more details). The virtual secretory machinery algorithm assigns each input protein to a specific secretory class and generates corresponding specific reaction lists (Panel A, see Materials & Methods; Table S5). The graphical representation of the secretory class number 45 (panel C) is shown in order to illustrate how each secretory class is characterized by a set of PTMs modifications and transport steps.

In our aim to integrate all available mechanistic knowledge into a scaffold for the study of the protein secretory machinery we used a bottom-up systems biology approach, which is based on collecting, assembling and integrating all relevant information and data by a combination of a comprehensive literature survey and searches in different databases (Figure 1A).

**Figure 2:**
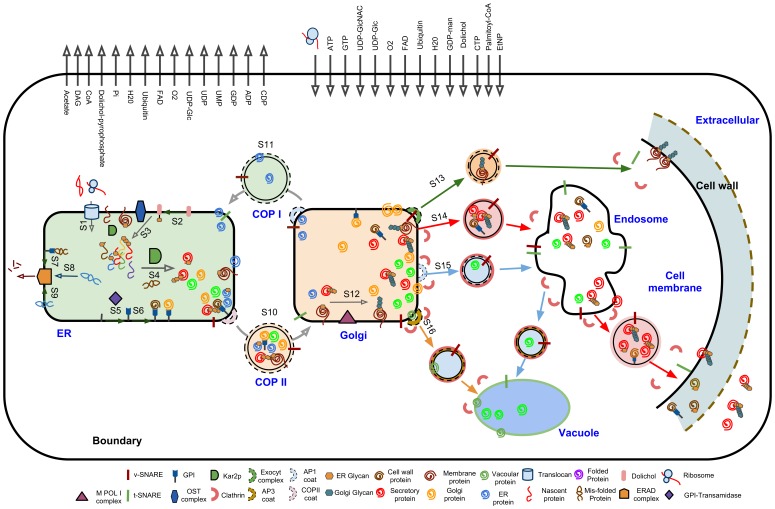
Schematic representation of ***Saccharomyces cerevisiae*** **secretory machinery model.** A schematic portray of the yeast secretory machinery including all the possible modification and transport steps. The model covers all the possible PTMs and transport routes of the yeast machinery. The machinery is divided into 16 subsystems (S1–S16). These subsystems are: S1: Translocation; S2: Dolichol pathway; S3: ER glycosylation; S4: Folding; S5: GPI biosynthesis; S6: GPI transfer; S7: ERADC; S8: ERADL; S9: ERADM; S10: COPII; S11: COPI; S12: Golgi processing; S13: LDSV (low density secretory vesicle); S14: HDSV (high density secretory vesicle); S15: CPY pathway; S16: ALP pathway. Each subsystem is shown with an arrow (For the full list of components of each subsystem and the associated template reactions see Table S1 and S2). The model has 8 compartments including endoplasmic reticulum (ER), Golgi, COPI, COPII, vacuole, endosome, membrane and extracellular (shown with vivid blue text beside them). The proteins located in the cell wall are considered to be extracellular proteins. The interaction of the model with the rest of the cell is based on the defined exchange reactions for the metabolic precursors, energy and electron carriers needed for the modification and transport processes in the machinery. The black rectangle around the machinery indicates the virtual system boundary which separates the secretory machinery from the rest of the cell and the exchange reactions are represented by arrows crossing this boundary.

The resulting reconstructed network includes 162 proteins and one RNA component (*SCR1*). These 163 components represent the core components of the protein secretory machinery that are directly involved in the translocation, folding, post-translational modifications and transport of the proteins as well as biosynthesis pathways leading to the precursors required for glycosylation and glycosylphosphatidylinositol (GPI) attachment (Figure 2;Table 1; and Table S1).

**Table 1:** The properties of the yeast secretory machinery model.

Yeast secretory model	Item	Databases	Number	Total
**y-PSIM**			5882× 7	5882
**Machinery component**	Protein		162	
	RNA		1	163
**Machinery reactions**	Template reactions		56	
	Complex formation		26	137
	Exchange reactions		25	
	Biosynthesis reactions		30	
**Network properties**	Input protein		1197	1197
	Protein specific reactions		11684 (for 552 proteins)	11684
	Component			
**Subsystems**	Number		16	16
	Compartment		8	8
**Knowledge source**	Publication		∼400	∼400
	Databases			3
		KEGG		
		UniProt		
		SGD		

To reduce the complexity, we divided the machinery into 16 subsystems (S1–S16) based on the function that each subsystem performs (Figure 2). In order to define the subsystems, we relied on the knowledge obtained from classical molecular biology experiments on specific proteins such as carboxypeptidase Y (CPY) [17], mating pheromone (alpha-factor) [18], H^+^-ATPase (Pma1p) [19] and alkaline phosphatase Phop8 (ALP) [20]. Although, the procedure of reconstruction provided us with a systematic repository of mechanistic information, it also allows to highlights the knowledge gaps. The 16 subsystems cover all the secretory machinery processes such as translocation, folding, sulfation, glycosylation and sorting while Most of the subsystems are located in the ER (S1–S9) (Figure 2).

The model contains 137 different reactions of which 56 are template reactions, 26 are complex formation reactions, 30 are biosynthesis reactions, and 25 are exchange reactions (Table S2). The template reactions are protein-specific and they formulate all the PTMs and sorting reactions. The complex formation reactions describe the formation of protein complexes that are involved in the template reactions. The *dolichol* and *GPI*-biosynthesis pathways, which provide the precursors for the glycosylation and the formation of *GPI*-anchored proteins, include the biosynthetic reactions. (Figure 1; Text S1; Table S2). A virtual system boundary was defined by formulating exchange reactions to separate the secretory machinery from other functional modules of the cell. These exchange reactions account for supply of co-factors and precursors needed for the modification, sorting and biosynthetic reactions (Figure 2; Text S1).

In the model reconstruction, we avoided lumping reactions in order to ensure proper gene-protein-reaction links for the individual steps. Furthermore, this allowed evaluating the role of individual steps, e.g. signal peptide recognition that has been shown to be the rate controlling step in translocation [21]. The reconstructed network condenses our current knowledge of the protein secretory system and it can be expanded and improved when new components or steps are identified.

### The PSIM (Protein Specific Information Matrix): A Knowledge Package for Modeling the Protein Secretory Machinery

Each secretory protein may contain in its sequence information for seven possible features: (1) the presence or absence of a signal peptide that indicates if the protein will be imported into the ER, (2) the number of *N*-linked and (3) *O*-linked glycosylation sites, (4) the number of disulfide bonds to be formed, (5) the presence or absence of anchoring with GPI (glycosylphosphatidylinositol), (6) the number of transmembrane spanning domains, and (7) the transport signal motif for the final localization (Figure 1B). Once these features have been established it is possible to determine which subsystems in the secretory machinery are required to processes each specific protein along the way to its functional destination (Figure 1C). The details and the assumptions made at this stage are given in the Text S1.

The required information for some of the selected features is available in databases such as *O-GlycBase* [22] which contains the *O*-linked glycosylation sites, or dbPTM, which integrates information about different post-translational modifications [23]. The information in these databases is not organism-specific and contains only proteins that have been studied experimentally. UniProt, as a high-quality source for protein information [24], contains information for all the mentioned features, experimentally or computationally derived and it has been used as our main preferred information source. We extracted all the information for the seven selected features for the whole yeast proteome (Table S7). This information was condensed into the **P**rotein **S**pecific **I**nformation **M**atrix (PSIM). Each row in the yeast PSIM (5882×7) represents a specific protein and each column represents one of the seven selected features. Therefore, each matrix cell contains information for a specific feature for a specific protein (Figure 1B). The possible combinations of the seven different features define theoretical 186 secretory classes, with each secretory class representing a unique combination of the seven different features (Figure 1B; Figure S1; see materials and methods and Text S1). The PSIM is organisim specific and extendable to contain more features for other PTMs and protein maturation steps specific to other organisms’ secretory machinery.

### Simulation of Yeast Secretory Machinery using the y-PSIM and Template Reaction List

Using the information condensed in template reaction list and secretory classes, we developed an algorithm (in Python programing language), which generates a protein specific reaction list for each protein (Figure 2B; Text S1). These reaction sets represent post-translational modifications and sorting processes that each protein undergoes through the machinery in order to reach its final functional state and destination.

After assigning each protein to one of the predicted secretory classes, it was found that the ER-Golgi secretory machinery potentially can process 1190 proteins. The PSIM of these proteins was used as input to the algorithms and the protein-specific reaction list for each of the proteins was generated (Table 1, for the complete genome-scale protein reaction list see Table S9).

**Figure 3:**
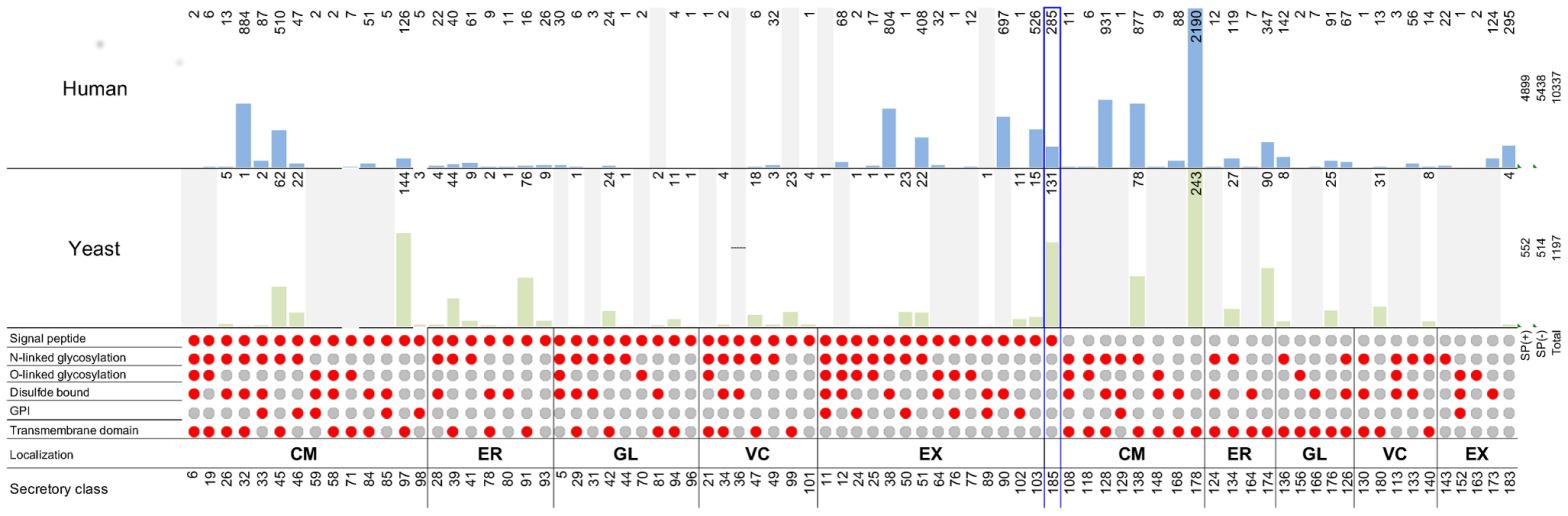
Comparative properties of the Yeast and Human secretory systems. The comparative distribution of the populated secretory classes in yeast and human are shown. Each secretory class is depicted by a column of red and gray spots that indicate if each feature is present or absent. The secretory classes are ordered based on their localization (shown with abbreviated text). Above the secretory classes the distribution bars for yeast (light green) and human (light blue) illustrate the number of proteins in each class. The protein numbers for each class are shown at the top of the bars. The empty classes are shown as grey bars and each class id’s can be found in the secretory class row at the bottom of the figure. The class 185, which includes proteins with signal peptide and unknown localization, is marked with blue rectangle.

Secretory classes can be divided into two main categories: The classes that have N-terminal signal peptide and the classes with signal sequence in their transmembrane domain, which are mostly plasma and endomembrane proteins. This classification is important as the proteins in each category differ in translocation mechanism, especially in the way they are targeted to the translocon complex [25] (see Text S1). From 1190 proteins, 683 of them are in the first category (SP+), 552 of them with known localization, and they fall into 34 out of the 104 secretory classes. The remaining 514 are in the second category (SP-) and they accommodated only in 9 secretory classes from 80 defined theoretical classes for this category (Figure 3).

It is noticeable that the SP+ secretory classes are more diverse but less populated than the SP- classes. Many of the 162 core components of the yeast secretory machinery are themselves processed by the secretory machinery, 68 of the core components belong to 13 different SP+ secretory classes and 65 belong to 5 SP- secretory classes. The remaining 30 components are cytoplasmic proteins mainly involved in vesicular transport processes (See Figure 3; Table S3 for more details).

Although the conventional secretory machinery is quite complex, recent investigation on the eukaryotic secretion systems has shown that there are alternative secretory pathways (called unconventional pathways), adding complexity to the secretion process [26]–[29]. For example, some of the yeast cell wall proteins have been confirmed to lack signal peptides (Nombela et al, 2006; Pardo et al, 1999) and in mammals the fibroblast growth factor 2 (FGF2) (that does not contain a signal peptide) uses an alternative pathway to reach the plasma membrane [30]. It still remains to be resolved how many of these 1190 are the main clients of the conventional secretory machinery which is the focus of this study. Therefore, we assumed for now they only use the conventional secretory machinery to be processed and transported to their functional station.

### Human PISM (h-PSIM) and Human Secretory Classes

One of the potential applications of the model is to be used as a scaffold for improving our understanding of the protein secretory machinery in other eukaryotic organisms such as humans. In order to illustrate this, we used the same approach to generate a PSIM for the human proteome (called h-PSIM, Table S8), which has dimensions 44540×8. The human secretory machinery is far more complex, and it is also tissue specific. However, it has been shown that the secretory machinery components are well conserved from yeast to human [31], which justifies using the yeast model as a scaffold. As expected, human cells use more SP+ secretory classes (46 out of 186) compared to yeast (34 out of 186). In human, SP+ secretory classes contain more proteins than in yeast. Figure 3 shows the detailed relative distribution of proteins in the different classes in human and yeast.

**Figure 4:**
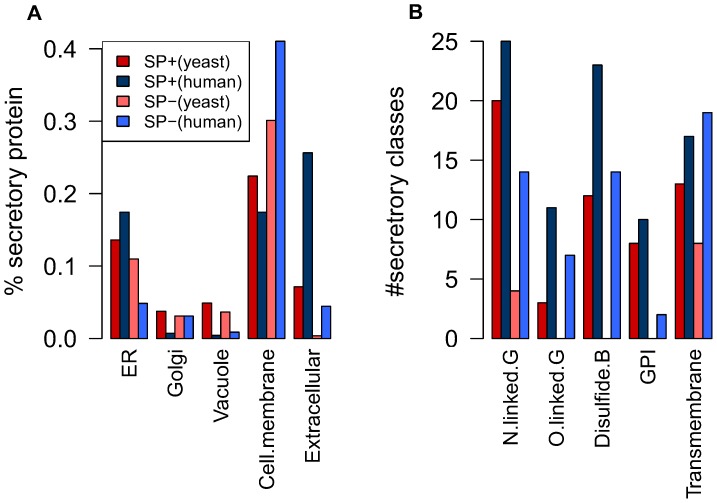
Comparison of secretory proteins distribution based on localization and secretory features information between yeast and human. (A) Comparative bar-plot indicates the distribution of the secretory proteins on different compartments. The percentage of secretory proteins(y axes) with different localization (x axes) is plotted for yeast and human. (B) Comparative bar-plot indicates the distributions of the secretory features (except signal peptide) on the secretory classes. The number of the classes that contain each of the features is plotted in the y axes.

In yeast and human, the fractions of the proteins which are in SP+ and SP- secretory classes are similar, For example in both human and yeast most of the plasma transmembrane proteins do not have signal peptide or almost all the extracellular proteins have signal peptide. However, this was not observed in the Golgi apparatus and the vacuole (or lysosome). (Figure 4A) [32].

Also, it is interesting that the fraction of the SP+ and SP- classes that are using different PTMs features are similar in yeast and human (Figure 4B).

The SP- secretory classes with transmembrane proteins which do not have signal peptides, they use signal sequences in their transmembrane domains to enter the ER. On the other hand, many of the plasma and endomembrane transmembrane proteins belong to SP+ classes.

### Functional Properties of the Secretory System in Yeast and Human Cells

The extension of the approach to explore the protein secretory machinery in human cells provides a systematic platform to investigate the distribution of secretory proteins in the different classes for both organisms (Figure 3).

Having defined the yeast and human SP+ and SP- secretory classes we performed a GO (gene onthology) enrichment analysis (see Materials and Methods), in order to evaluate biological functions of the proteins in the different secretory classes. Comparing GO enrichment for yeast proteins secreted by the SP- and SP- secretory classes (Table 2) we found that GO terms related to the cell wall organization and biogenesis show the most statistically significant (lowest p-value) enrichment in the SP- secretory classes (Table 2; Table S10). Yeast cells are surrounded by a rigid and thick (∼200-nm) but also dynamic wall structure made of glycans and mannoproteins, which plays a key role in keeping the cell shape and integrity, maintaining osmotic stability, enable flocculation and adherence [33]. The yeast cell wall comprises 15–30% of the cell dry weight and its main components are different glycans and secreted proteins [34], [35]. In addition, it is claimed that 20% of the yeast genome deals with cell wall biogenesis [36]. All this evidence is consistent with the enriched GO terms in the conventional secretory machinery being related to cell wall biogenesis.

**Table 2:** GO enrichment analysis of SP+ and SP- secretory classes in yeast and human.

Organism	Secretory Type	GOID	Term	Corrected p-value
**Yeast**	**SP+**	GO:0071554	cell wall organization or biogenesis	9.06E-50
		GO:0070882	cellular cell wall organization or biogenesis	7.47E-49
		GO:0007047	cellular cell wall organization	4.41E-40
		GO:0045229	external encapsulating structure organization	4.41E-40
		GO:0071555	cell wall organization	4.41E-40
		GO:0071852	fungal-type cell wall organization or biogenesis	1.17E-28
	**SP-**	GO:0006810	transport	2.61E-137
		GO:0051234	establishment of localization	2.64E-133
		GO:0051179	localization	1.91E-123
		GO:0055085	transmembrane transport	2.80E-85
		GO:0016192	vesicle-mediated transport	1.20E-56
		GO:0071702	organic substance transport	1.18E-41
**Human**	**SP+**	GO:0005102	receptor binding	7.06E-140
		GO:0005125	cytokine activity	1.64E-84
		GO:0005179	hormone activity	1.03E-58
		GO:0005539	glycosaminoglycan binding	4.74E-58
		GO:0001871	pattern binding	7.90E-57
		GO:0030247	polysaccharide binding	7.90E-57
	**SP-**	GO:0004930	G-protein coupled receptor activity	0
		GO:0004984	olfactory receptor activity	0
		GO:0004888	transmembrane signaling receptor activity	9.81E-243
		GO:0038023	signaling receptor activity	2.91E-209
		GO:0004872	receptor activity	1.73E-156
		GO:0004871	signal transducer activity	8.90E-152

Significant GO terms (p-values<0.001) is listed here (see Materials and Methods). For the full list of the GO terms and corresponding statistics refer to the Table S10–13.

GO enrichment analysis for the SP- secretory classes shows that these proteins mainly are involved in transport and localization processes such as transmembrane transport (ion transport), vesicle mediated transport dealing with protein localization (COPI, COPII, SNARE complex etc.) etc. (Table 2; Table S10–13).

We also performed GO enrichment analysis for the human SP+ and SP- secretory classes. The results for the SP+ secretory machinery in human cells show, in contrast to yeast, where all the proteins in this group are annotated, that there are 2,557 non-annotated proteins containing a signal peptide (about 50% of all potential secretory proteins). Focusing on the annotated proteins, some of the GOs that indicate a statistically significant enrichment are those related to receptor binding, cytokine activity, hormone activity etc. (Table 2; see Table S14 for details).

For proteins belonging to the human SP- secretory classes 3,003 proteins are not annotated (∼60%), whereas GO terms related to signalling are the most enriched among these proteins (Table 2; see Table S13 for details).

### Energy and Metabolic Demand Estimation of the Secretory Machinery

**Figure 5:**
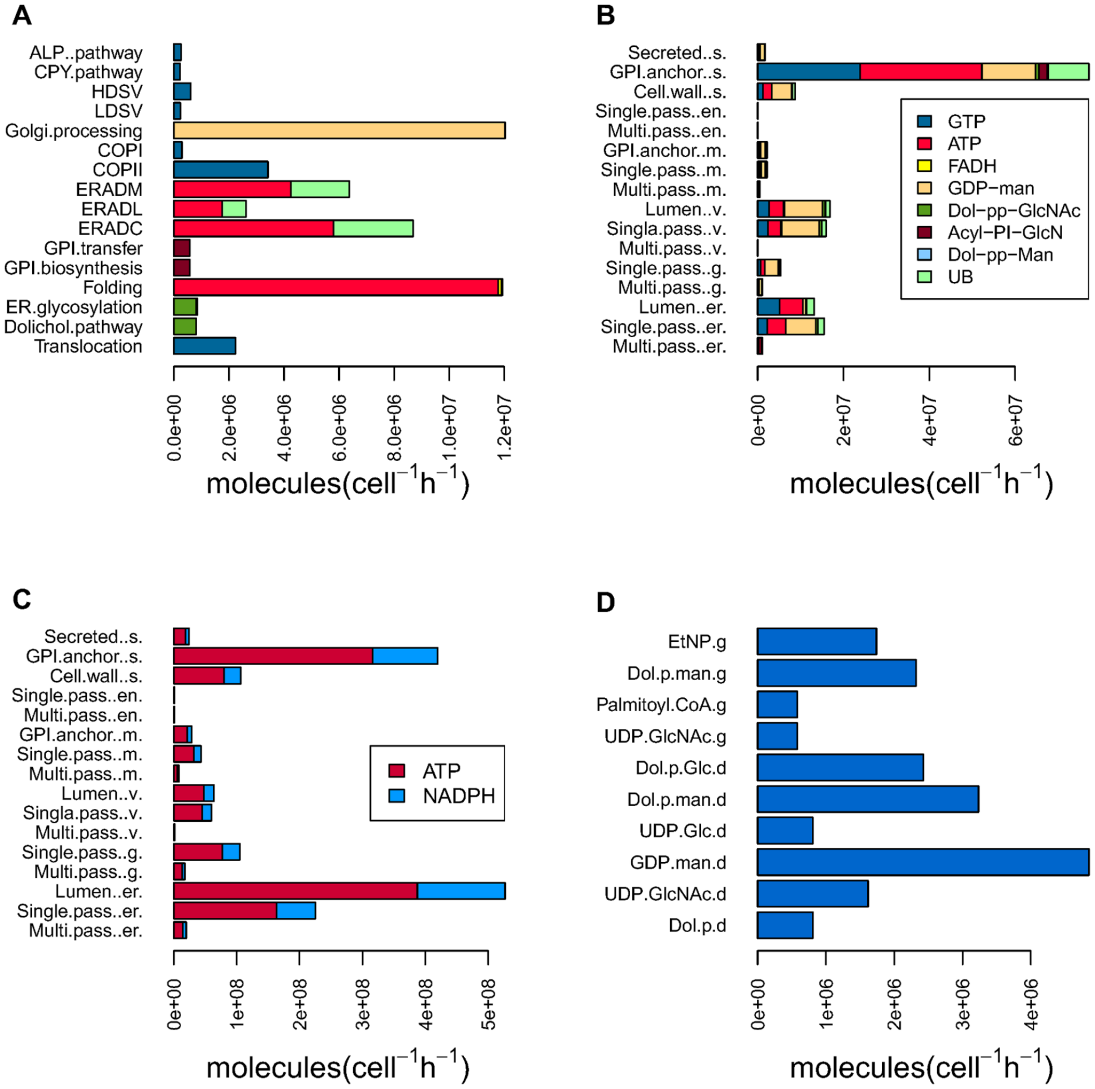
Estimation of the secretory machinery metabolic demands. (**A**) Energy cost and metabolic demand of each subsystem. Yeast steady-state protein abundance data [37] were integrated with the reconstructed network (a total 11684 of protein specific generated reactions) to estimate the different metabolic demands (molecules cell^−1^ h^−1^) for different subsystems. The horizontal bar-plot shows the calculated the metabolic precursors consumption (*x* axes) for each subsystem (*y* axes). UB (Ubiquitin) was considered to be metabolite as it is precursor for labelling mis-folded proteins in order to target them for degradation (see Materials & Methods). (**B**) The metabolic demands have been calculated for each compartment. In each compartment, the proteins have been divided into be single or multiple- pass transmembrane, GPI-anchored or luminal. Panel C shows the bar-plots of the protein synthesis costs (ATP and NADPH). Panel D shows the metabolic costs of the Dolichol and GPI biosynthesis pathways (the metabolic precursors name are indicated in the *y* axes ended by *d* or *g* representative for Dolichal or GPI biosysntheis).

The other impotent potential applications of the reconstructed genome-scale network for the secretory machinery is to estimate the usage of various co-factors (ATP and GTP) and metabolic precursors for glycosylation or sulfation such as GDP-man or FADH2. This allows linking the secretory machinery with the rest of the cellular metabolic processes. Using protein abundance data for yeast [37] we calculated the metabolic precursor costs for each of the proteins passing through the machinery (cell^−1^ h^−1^) (Figure 5A, Table S4). GTP usage accounts for the amount of the energy needed for the translocation and transportation through the machinery [38]–[40], and therefore proteins (or their corresponding secretory classes) with high GTP usage generally have more vesicular transport steps before the proteins reach their final localization. ATP is used for degradation and folding [41]–[43] and FADH2 [44]–[46] is used in connection with disulfide bond formation (see the Materials and Methods). The estimation of co-factor usage is based on the potential 11,591 protein specific reactions needed to process the 552 SP+ proteins. However, only 259 of these proteins have available abundance data. The reminding 291 proteins are likely to be either non-present or very low abundant and we therefore set their abundance arbitrary to one protein per cell. Hereby we could keep these secreted proteins in the model for annotation purposes but in our model they had a very minor contribution in estimation of the metabolic costs. Based on this we estimated the metabolite consumption as cell-1 h-1 for each subsystem (Figure 5A). We considered UB (Ubiquitin) as a metabolite as it is used as a precursor for labeling mis-folded proteins targeted for degradation. The Dolichol pathway uses precursors from lipid metabolism (dolichol synthesized from farnesyl-PP) [47], whereas the central carbon metabolism and nucleotide metabolism provide three different nucleotide-activated sugar donors for the dolichol pathway including: UDP-N-acetylglucosamine (UDP-GlcNAc) (provided by the Leloir pathway) [48], GDP-mannose (GDP-Man) [49] and UDP-glucose (UDP-Glc) [50]. The supply of all these metabolites has been reported to be flux controlling [51]. In order to estimate the demand for dolichol pathway metabolic precursors, we calculated the amount of core glycan that is needed for the glycosylation of all the predicted glycosylation sites in proteins that pass through the secretory machinery.

In addition, we calculated the metabolic costs of the dolichol and GPI biosynthesis pathways separately to give a better resolution of these two biosynthetic pathways that are connecting the secretory machinery to the metabolic network. Dol-p-man (dolichyl phosphate mannose) and UDP-GlcNAc (Uridine diphosphate-N-acetylglucosamine) are the two metabolites that connect these pathways (Figure 5D; Table S4). While we calculated the metabolic demands for each subsystem, we also explored the most abundant proteins passing through the secretory pathway (see Table S5), and it is interesting that the two most abundant proteins in the yeast cell are secretory proteins. Cwp2p (UniProt: P43497) is the most abundant protein in the cell and it is a very short GPI-anchored mannoprotein (90 aa) which is the major constituent of the cell wall (clustered in secretory class 102). The second most abundant protein is Pma1p (UniProt: P37367), which is a plasma membrane P2-type ATPase that pumps protons out of the cell (905 aa, clustered in the secretory class 178) (see Figure S3 for other proteins). It is interesting to note that Pma1p does not have a signal peptide and is potentially secreted via the alternative secretory pathway. Most of the other highly abundant proteins in the yeast cell are involved in metabolism; chromatin assembly and translation [37]. It is noticeable that among the machinery subsystems, ERAD and COPI subsystems both have a high average protein abundance regarding their involved components compare to the other subsystems (Figure S4).

We are aware that our model represents a simplification so it is important to note that our estimations of precursor requirements, are based on current knowledge on the yeast secretory machinery and accordingly they are uncertain for subsystems like folding or ERAD for which we do not have protein specific stoichiometry. Also in terms of glycosylation there may be uncertainties as not necessarily all glycosylation sites are being used all the time [52].

We also estimated the metabolic costs of processing the whole set of proteinspresent in some cellular compartments which are secretory machinery clients (Figure 5B). The results shows that secretory proteins connected to the cell wall with GPI-anchored chains are the most costly proteins in terms of folding, PTMs and transport steps. This is also in accordance with the GO enrichment analysis (Figure 5B). The ER and vacuole proteins are the second most costly group. Interestingly, the results show that single-pass membrane proteins have higher processing costs than the multi-pass proteins, and proteins targeted to the ER and the vacuole membranes have higher metabolic demands than proteins targeted to the cell membrane. This ration can change if we include the cost for SP- classes’ proteins to the calculation. We also calculated the synthesis cost (ATP and NADPH) of the secretory proteins, and this showed that the ER proteins (especially those located in the lumen) have the highest synthesis cost and GPI-anchored proteins localized in the cell wall have the second highest synthesis costs (Figure 5C). As for metabolic costs the single-pass transmembrane proteins have higher synthesis costs than the multiple-pass transmembrane proteins (Figure 5C). Both the ER and the cell wall have proteins with high abundance and many PTM features.

### Evaluation of Engineering Strategies for Improving the Secretory Machinery

**Figure 6:**
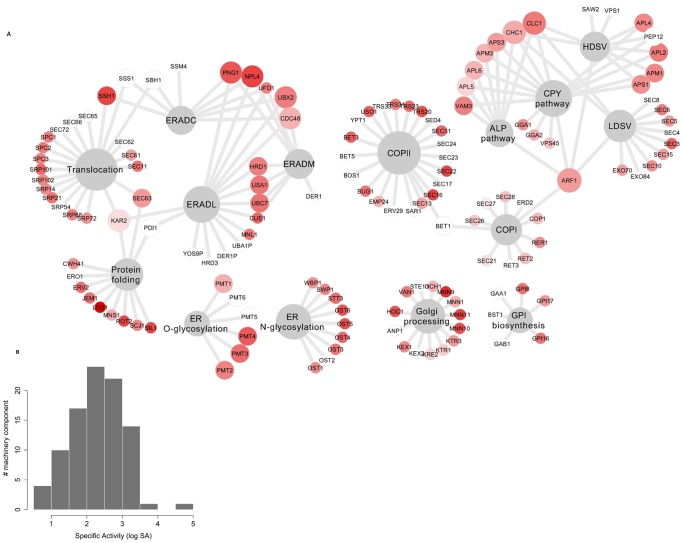
The specific activity (SA) network of the components of the yeast secretory machinery at exponential growth. The network representation of the machinery component SA (specific activity) in panel A shows how the components (circle nodes) are involved in one or various subsystems (diamond) processes. The graph is produced in Cytoscape [63] and the nodes color and size are weighed by the node degree and SA respectively (see Table S6 for Cytoscape input file).The logarithmic histogram (log10) of the SA (panel **B**) shows how the machinery components are distributed based on their specific activity.

Metabolic engineering of the secretory pathway is often based on altering the expression of some of the machinery components with the objective to increase secretion of a particular protein (often a heterologous) [53], [54]. Two key aspects to consider in this process are choosing the proper target(s) and optimizing the expression level. Although, many improvements have been done in this area, a systems biology approach may give a holistic picture of the secretion system and hereby suggests new targets for metabolic engineering [54], [55]. To evaluate the activity of the individual components of the secretory pathway we used the steady-state protein abundance data [37] and our protein-specific reaction list to estimate the activity of the functional components of the system. A specific activity (SA) measure for each component was defined as the number of its catalytic cycles per cell per hour, in steady-state (see Materials and Methods). The SA for each component is a function of its abundance and the amount of the proteins that it catalyzes in steady state per cell per hour (Figure S2). A logarithmic histogram of the SA for the different machinery components shows that the SA follows a normal distribution (µ = ∼2.2 and o' = ∼0.7) (Figure 6B). Accordingly, there are few proteins with high SA and evaluation of the proteins with highest specific activities shows that they are not limited to a specific subsystem (Table 3).

**Table 3:** The components with high specific activity (with log (SA)>3).

Model component	Protein abundance (cell ^−1^ h^−1^)	SA (log10)	Subsystem
**LHS1**	136.79	4.63	Protein folding
**MNN9**	1629.89	3.85	ERADC
**MNN11**	3475.70	3.47	Golgi processing
**SEC16**	357.97	3.44	COPII
**NPL4**	1054.72	3.43	ERADL
**SEC22**	395.62	3.40	COPII
**SSH1**	704.48	3.39	TC
**SIL1**	2420.02	3.38	Protein folding
**ROT2**	238.01	3.33	Protein folding

Figure 6A shows a graph representing the connectivity between the subsystems and components of the yeast secretory pathway with their SA activity mapped to the node color (components). Some of the components are involved in several subsystems (such as Kar2p) and they are expected to have a higher impact on the function of the machinery if their expression level gets modified. On the other hand, the overexpression of proteins with high SA (which process a high number of molecules per unit of time) is also expected to have a higher impact than overexpression of proteins with lower SA.

For example, in the protein folding subsystem the Lhs1p is the least abundant (∼139 molecules) component with the highest SA (∼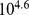) and Kar2p has a high abundance (∼336941 molecules) with low SA (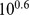 cell^−1^ h^−1^). Kar2p is the main chaperon in the ER [56]. Lhs1p and Sil1p (2420 molecules and with a high SA of 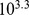 cell^−1^ h^−1^) are two NEFs (nucleotide exchange factors) which have ATPase activity and regulate the Kar2p ATP turnover [57]. Each time Kar2p performs a catalytic cycle, it needs the presence of Lhs1p and Sil1p to restart a new cycle. However the mentioned NEFs have high SA (much lower abundances than Kar2p) and it is therefore likely that their activity is a bottleneck for the activity of Kar2p. As the ER is crowded, over-expressing these proteins with low abundance and high SA could therefore be more effective than the overexpression of *KAR2*. There is some evidence in favor of the effect of the modulation of these chaperones in improving heterologous protein production [58]. On the other hand, it has been shown that over-expression of *KAR2* has not positive effect on the secretion level, while decreasing its expression shows negative effect [59].

In summary, for the production and secretion of a particular protein in yeast as a cell factory, the reconstructed model provides the three type of information including: the secretory class that targeted protein belongs which enables to have a list of mechanistic specific reactions with the catalyzing components, the estimation of the metabolic demands associated to maturation and sorting steps and the SA information about the natural capacity of the involved machinery component in corresponding processes. This information advances designing strategies to engineer the secretory machinery with the objective of high production rate.

### Conclusions

In this work, we applied, for the first time, a genome-scale modeling approach to study the complexity of the eukaryal protein secretion pathway. We used a bottom-up network reconstruction method. The model contains detailed mechanistic knowledge of the secretory machinery and can be used to integrate -*omics* data in order to achieve a better understanding of the eukaryal secretion system. Identifying secretory classes allowed grouping the secretory proteins based on their PTMs and sorting features. Furthermore, generating protein-specific reaction lists and combining these with yeast protein abundances enabled estimation of the metabolic demands of the secretory machinery in a protein-specific manner. Additionally, the SA (specific activities) of the machinery components were estimated which provides information about the natural capacity of the machinery components catalytic activity.

In a nutshell, the reconstruction approach and the ‘PSIM’ matrix provide a framework for (i) capturing the genome-scale mechanistic details of the secretory machinery; (ii) integrating and analysing high-throughput data for evaluation of the function of different parts of the machinery and hereby increasing our knowledge of systemic properties; (iii) offering a systems biology framework for engineering industrial and therapeutic protein secretion strategies; (iv) and finally for connecting the model to other cellular processes such as metabolism.

## Methods

### Data Acquisition

We used UniProtKB for retrieving yeast and human proteome information for the selected PTM features including signal peptide, *N*-linked glycosylation, *O*-linked glycosylation, disulfide bonds, transmembrane domain, *GPI*-anchoring, and localization. The signal peptide is a critical feature to determine if the protein is a secretory protein or not and according to some contradiction between the UniProt and SGD signal peptide information, we used the combination of signal peptide information between UniProt KB and SGD (Text S1). All feature extraction steps were performed automatically using the Python programming languages (www.python.org). Uniprot, SGD, and KEGG databases were used throughout the reconstruction approach in iterative manner.

### Reconstruction Process

The network reconstruction process of the *S. cerevesiae* secretory machinery consisted of four steps. First, based on a comprehensive literature survey (research and review papers and book chapters) on the yeast secretory pathways, the functional subsystems constituting the secretory machinery were defined. The resulting list of components was used as a starting point from which more components and corresponding publications (Table S1) were added by doing a systematic search in the Saccharomyces Genome Database (SGD) [60]. In a second step, each of the identified processes was formulated as a pseudo-chemical reaction with as detailed mechanistic knowledge as possible (Table S2). The resulting reactions were classified as template reactions, complex formation reactions and biosynthetic reactions (providing GPI and glycan donors) (Table 1; Table S2; Text S1). The machinery is connected to the rest of the cell by defined exchange reactions providing the energy and the metabolic precursors needed for the biosynthetic reactions of the model (Text S1). In a third step, with the aim of generating protein-specific reaction lists, we defined the secretory classes based on the combinatorial space of secretory protein modification and sorting features. The features defining our combinatorial space are: *signal peptide for the ER (present or absent)*, *N-linked glycosylation site (present or absent), O-linked glycosylation site (present or absent)*, *disulfide bound (present or absent)*, *GPI-anchored (yes or no)*, *transmembrane domain (present or absent)* and *localization (five possible final destinations)* (Text S1; Figure 2). Each secretory class corresponds to one particular combination of values for the mentioned features, for example: signal peptide (+), disulfide(-), N-linked glycosylation(+), O-linked glycosylation(-), transmembrane (+), localization(cell membrane) (“+”or “-” is indicating the presence or absence of the feature). After mapping the yeast proteome SGD IDs to the UniProt database, the selected feature information was obtained by parsing each UniProtKB protein information file using a python script. Based on the retrieved information, the protein specific information matrix (PSIM) was built; in which each row corresponds to one specific protein and each column provide the information for a specific selected feature such as signal peptide etc. In order to define the secretory classes only the values ‘+’and ‘-’ are used, but the PSIM matrix contains quantitative information, e.g. the actual number of predicted glycosylation sites for each protein. With the PSIM matrix, it is possible to define a protein specific reaction list for each protein. In a fourth step, a virtual secretory machinery algorithm coded in Python to simulate the secretory machinery defines the stoichiometry of the related reactions from the template reaction list. As an output, the protein specific reaction list was generated for the yeast 550 secretory machinery proteins (Table S9).

### GO Enrichment Analysis

For the GO enrichment analysis of the secretory classes (in both yeast and human), the GO::TermFinder [61] was used to find the most related GO terms for each class. The default parameters have been used in the search (cell processes ontology aspect and *p-value* of 0.01) and the top 10 GO terms were selected to represent the functional role of each secretory class (Table 2; Table S110–14).

### Estimation of Machinery Metabolic Cost in Steady-state Integrating Proteomics Data

The steady-state protein abundance data of *S. cervesiae* [37] were used for the estimation of the metabolic and energy costs of the secretory machinery. For this, we first need to know the processing rate of each machinery protein product 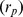 in steady-state, which is given by equation 1, where 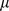 is the specific growth rate and 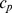 is the steady state concentration of each protein.
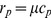
(1)


The rate of each of the machinery reactions can be calculated from equation 2 where, 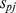 is the stoichiometry of specific reaction of the machinery (0 or 1) involved in the production of the specific protein *p* and 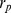 is the protein production rate for this specific protein as mentioned.
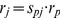
(2)


Finally, the consumption rate of the metabolites of interest in steady-state is calculated from equation 3 where, 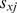 is the stoichiometry of the corresponding metabolite *x* (such as ATP, GTP, GDP-man etc.) in reaction *j* and 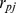 stands for the reaction rate for a specific protein *p*.
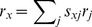
(3)


We calculated the metabolic costs for all the template reactions and for each metabolite and plotted them based on the machinery subsystems (Figure 5A; Table S4). For better resolution the same kind of calculation was used to estimate the *Dolichol* and *GPI* biosynthesis metabolic cost to produce the needed precursors for ER glycosylation and GPI transfer in steady-state (Figure 5D).

In order to calculate the synthesis costs for each of the yeast proteins, we summed up the amino acid biosynthesis energy cost with its translational machinery polymerization cost. The cost for each protein 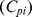 is calculated from equation 4 where, 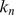 is the number of each of the twenty amino acids in the protein, 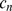 is the cost of the corresponding amino acid biosynthesis and the second expression is the translational energy cost of the whole sequence. The amino acid biosynthesis costs are taken from [62] and the 4 ATP equivalents are necessary for the formation of each peptidic bond (charging of tRNA: 2 ATPs; binding of tRNA to Ribosome: 1 GTP; elongation: 1 GTP).
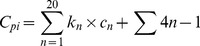
(4)


### Estimation of Machinery Component Activity in Steady-state

The specific activity (*SA*) for each machinery component is defined to be the number of its catalytic cycles in 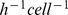
in steady-state. The
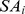
is the specific activity of the 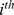 element of the machinery which can be calculated from equation 5, where 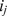 is the reaction *j* catalytic rate for production of 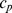
(calculated from equation 2), 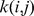
is the stoichiometry of the 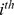
component in reaction *j*, and 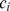 is the concentration of the 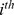 component itself in steady-state.
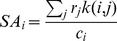
(5)


The resulting *SA’s* for each of the machinery components were plotted using Matlab (The MathWroks, Inc, Natick, MA) as a *histogram* function to cluster the machinery components with different ranges of their SA (Figure 4D; Table S4 and S6).

## Supporting Information

Figure S1 **All of the defined secretory classes for yeast secretory machinery.** The 186 defined secretory classes (starts from class 0 to class 185) with their specific feature combinations. The red spot shows the existence of a feature and gray spot indicates the absence. The first 104 class are the classes with signal peptide and the remaining 82 are without signal peptide. The class ids are depicted in the secretory class column. Features description is given at top of each feature column.
(TIF)

Figure S2 **The correlation of the main component of the secretory machinery specific activity(SA) and protein abundance**.The yy-plots for the SA(log _10_)(cell^−1^ h^−1^) and corresponding protein abundance(molecules cell^−1^) of each of the subsystems is shown. The subsystem names are located above each plot.
(TIF)

Figure S3 **The most metabolic demanded proteins of the secretory machinery.** For each of the metabolic precursors (shown at the bottom of each plot) the top 5 proteins are plotted. For the annotation of these proteins see the Table S2. The bottom plot shows the abundance distribution of the highly demanded proteins.
(TIF)

Figure S4 **Average abundance of the yeast secretory machinery subsystems component.**
(TIF)

Table S1 **The components of the core protein machinery.** The components of the machinery which are used as the core model components are provided in this table with the corresponded description.
(XLSX)

Table S2 **Template reactions list for Saccharomyces cerevisiae secretory machinery model.** This table provide a detailed description of the model template reaction list with the components and corresponding reference for each specific template reaction. These reactions are used as input for the algorithm.
(DOCX)

Table S3 **Yeast and human secretory classes**. This table provides the detailedinformation about the yeast and human populated secretory classes with the SGD and UniProt ID for members for each class.
(XLSX)

Table S4 **Subsystem level metabolic demand estimation for yeast secretory proteins.** The various metabolic precursors’ estimation is provided in this class based on each subsystem consumption in genome scale in cell^−1^ hour^−1^ in steady state.
(XLSX)

Table S5 **SA of the yeast machinery component with protein abundance data.** The estimated specific activity for each component which have the steady state protein abundance data.
(XLSX)

Table S6 **Cytoscape input file for the machinery component with estimated specific activity.**
(XLSX)

Table S7 ***Saccharomyces cerevisie*** **PSIM.** The yeast proteome information for post translational modification and localization information is shown in Table S7 which used as input for the algorithm.
(XLSX)

Table S8 **Human PSIM.** The human proteome information for post translational modification and localization information is shown in Table S10.
(XLSX)

Table S9 **Genome-scale protein specific reaction list for 550 yeast potential secretory proteins.** This table provide the entire reaction list for the highly potential yeast secretory machinery clients in protein specific manner.
(XLSX)

Table S10 **Yeast SP+ secretory proteins GO enrichment.**
(XLSX)

Table S11 **Yeast SP- secretory proteins GO enrichment analysis.**
(XLSX)

Table S12 **Human SP+ secretory proteins GO enrichment analysis.**
(XLSX)

Table S13 **Human SP- secretory proteins GO enrichment analysis.**
(XLSX)

Table S14 **The descendent metabolic cost for each of the secretory client’s production in steady state cell-1 hour-1.**
(XLSX)

Text S1 **The reconstruction approach.** The reconstruction approach and assumption of the model is described in details in Text S1.
(DOCX)
