# Supplementary figures and images for: Genome-Scale Modeling of the Protein Secretory Machinery in Yeast
Source: PLoS One. 2013 May 7;8(5):e63284. doi: 10.1371/journal.pone.0063284 (PMC3646752; doi:10.1371/journal.pone.0063284)

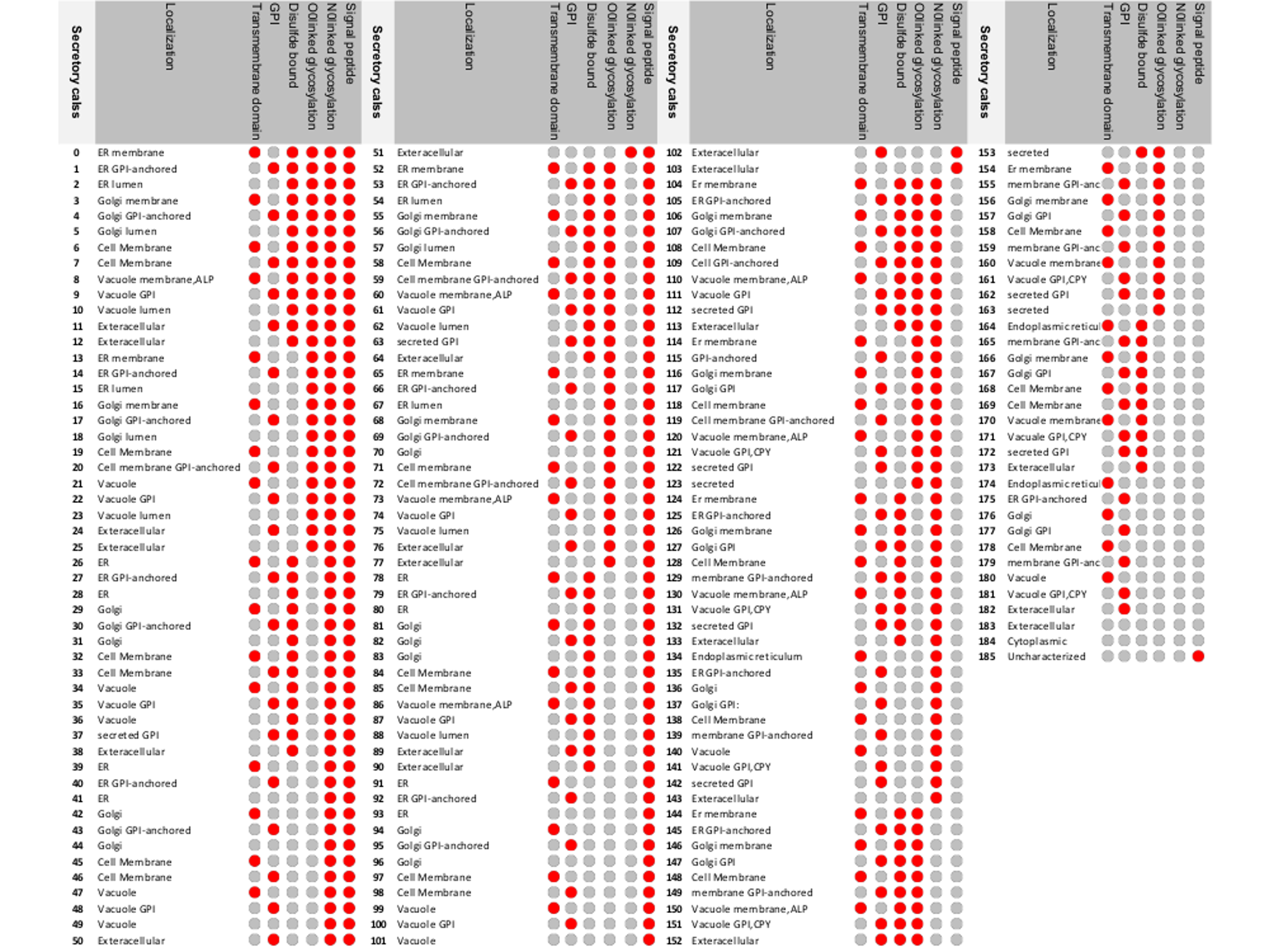

Supplement: Figure S1 — All of the defined secretory classes for yeast secretory machinery. The 186 defined secretory classes (starts from class 0 to class 185) with their specific feature combinations. The red spot shows the existence of a feature and gray spot indicates the absence. The first 104 class are the classes with signal peptide and the remaining 82 are without signal peptide. The class ids are depicted in the secretory class column. Features description is given at top of each feature column. (TIF) [file pone.0063284.s001.tif]

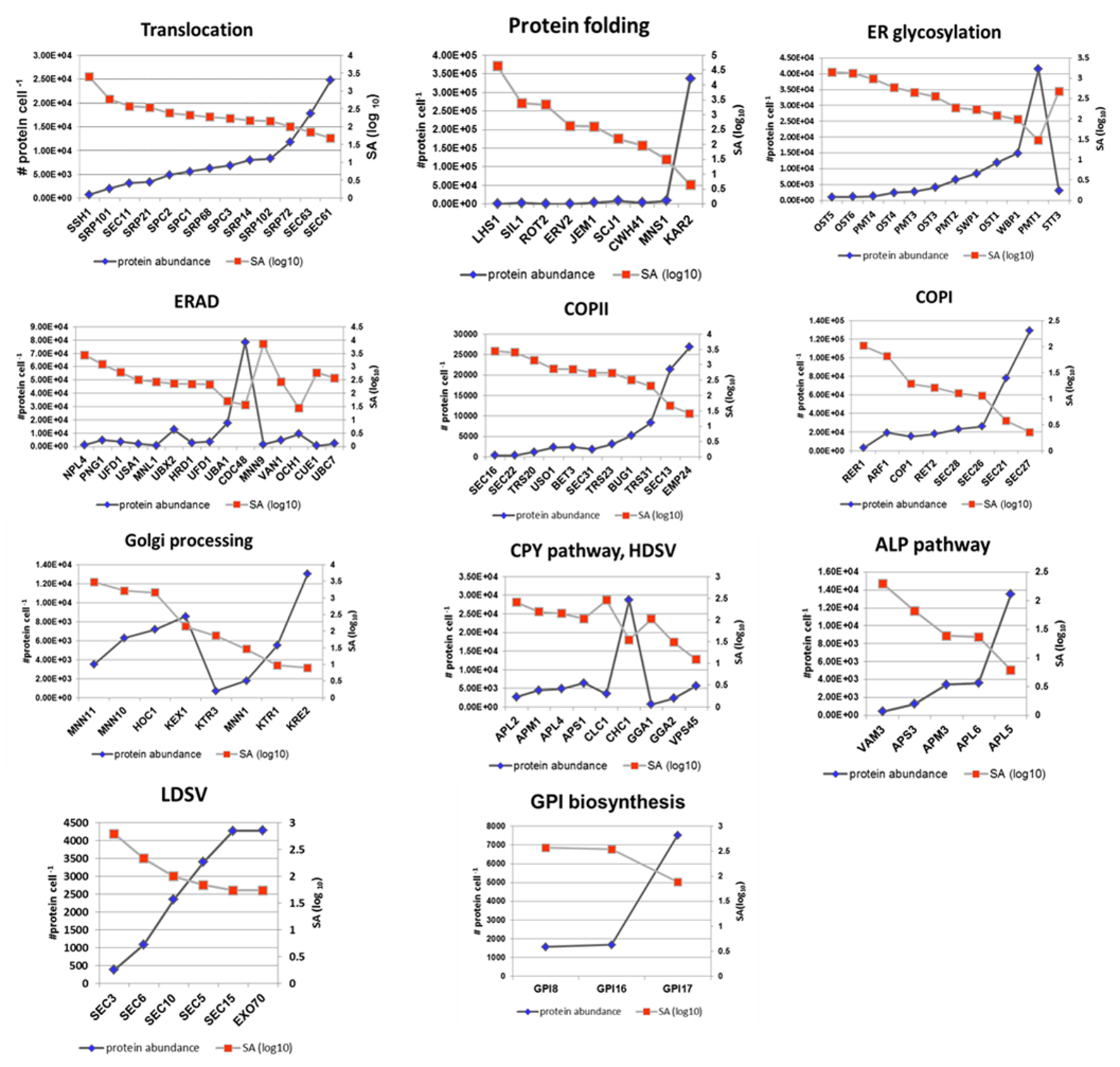

Supplement: Figure S2 — The correlation of the main component of the secretory machinery specific activity(SA) and protein abundance.The yy-plots for the SA(log 10)(cell−1 h−1) and corresponding protein abundance(molecules cell−1) of each of the subsystems is shown. The subsystem names are located above each plot. (TIF) [file pone.0063284.s002.tif]

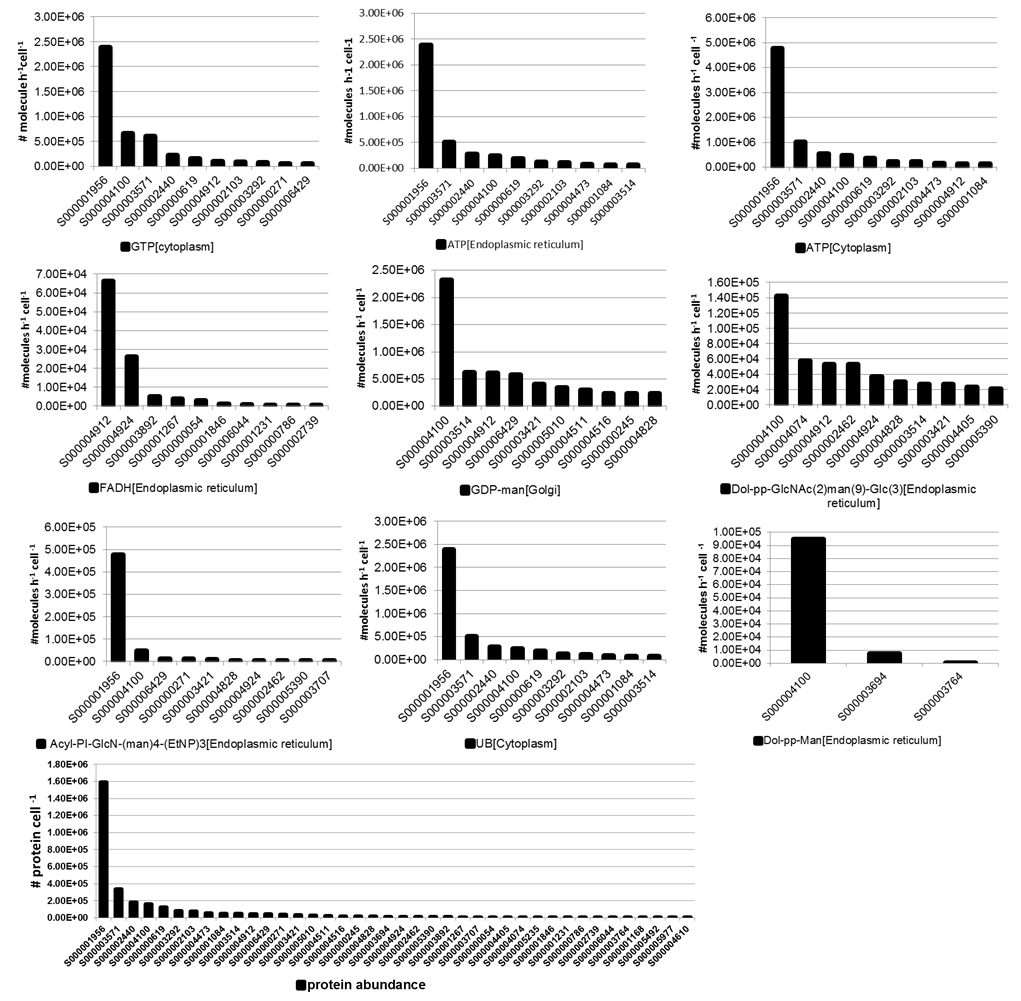

Supplement: Figure S3 — The most metabolic demanded proteins of the secretory machinery. For each of the metabolic precursors (shown at the bottom of each plot) the top 5 proteins are plotted. For the annotation of these proteins see the Table S2. The bottom plot shows the abundance distribution of the highly demanded proteins. (TIF) [file pone.0063284.s003.tif]

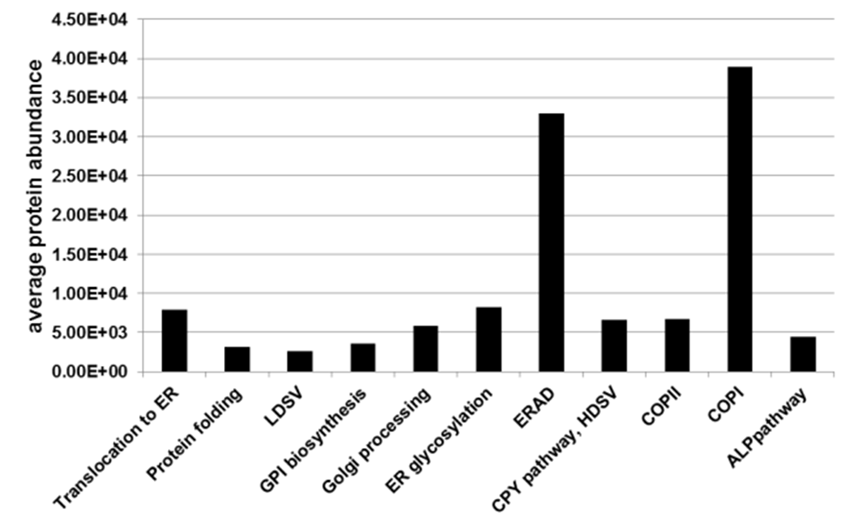

Supplement: Figure S4 — Average abundance of the yeast secretory machinery subsystems component. (TIF) [file pone.0063284.s004.tif]
