# Supplementary material for: Genome-Scale Modeling of the Protein Secretory Machinery in Yeast
Source: PLoS One. 2013 May 7;8(5):e63284. doi: 10.1371/journal.pone.0063284 (PMC3646752; doi:10.1371/journal.pone.0063284)
Supplement: Table S2 — Template reactions list for Saccharomyces cerevisiae secretory machinery model. This table provide a detailed description of the model template reaction list with the components and corresponding reference for each specific template reaction. These reactions are used as input for the algorithm. (DOCX) [file pone.0063284.s006.docx]

**Table S2. Template reactions list for *Saccharomyces cerevisiae* secretory machinery model.**

| **NO** | **Reaction Abbreviation** | **Reaction Name** | **Reaction Template** | **Directionality** | **Subsystem** | **Components** | **Comments** | **References** |
| --- | --- | --- | --- | --- | --- | --- | --- | --- |
| 1 | SRPC | SRP complex formation | Srp14p[Cytoplasm] + Srp21p[Cytoplasm] + Srp68p[Cytoplasm] + Sec65p[Cytoplasm] + Srp72p[Cytoplasm] + scR1[Cytoplasm] + Srp54p[Cytoplasm] <=> **SRP**[Cytoplasm] | reversible | translocation from cytosol to ER | [***Srp14p***](http://www.yeastgenome.org/cgi-bin/locus.fpl?locus=srp14)***,***[***Srp21p***](http://www.yeastgenome.org/cgi-bin/locus.fpl?locus=srp21)***,*** [***Srp68p***](http://www.yeastgenome.org/cgi-bin/locus.fpl?locus=srp68)***,*** [***Srp72p***](http://www.yeastgenome.org/cgi-bin/locus.fpl?locus=srp72)***,*** [***Sec65p***](http://www.yeastgenome.org/cgi-bin/locus.fpl?locus=sec65)***,***[***ScR1***](http://www.yeastgenome.org/cgi-bin/locus.fpl?locus=scr1)***,*** [***Srp54p***](http://www.yeastgenome.org/cgi-bin/locus.fpl?locus=srp54) | SRP is a cytosolic ribonucleoprotein which targets the SRP-dependent proteins to the ER membrane .Core components for SRP include: *SRP14p*, *SRP21p*, *SRP68p*, *SRP72p*,*SEC65*, and *SRP54*, and the RNA (termed scR1 and encoded by *SCR1*). Proteins and RNA assemble into a core complex in the nucleus (except Srp54p). It is use the NES pathway to export into the cytoplasm to bind to the Srp54p which is unique in yeast.  For simplicity we assumed that the SRP complex formation happens in cytoplasm (supplementary text S1). | **[**[**1**](#_ENREF_1)**]** |
| 2 | SRC | SRC complex formation | Srp101p[Cytoplasm] + Srp102p[Cytoplasm] <=> **SRC**[Cytoplasm] | Reversible | Co-translocation from cytosol to ER | [***Srp101p***](http://www.yeastgenome.org/cgi-bin/locus.fpl?locus=srp101)***,*** [***Srp102p***](http://www.yeastgenome.org/cgi-bin/locus.fpl?locus=srp102) | SRPC interacts with the signal peptide of the nascent proteins (to form RNC complex) then by interaction between SRP and a signal receptor complex (SR), encoded by SRP101 and SRP102 it attaches to the ER membrane. Finally, the RNC is transferred to the translocon, a protein-conducting membrane channel, and SRP and the SR dissociate. GTP bind to both SRP (via the Srp54p subunit) and the SR is critical for their interaction. | **[**[**1**](#_ENREF_1)**,** [**2**](#_ENREF_2)**]** |
| 3 | SEC61C1 | SEC61C1 complex formation | Sec61p[Endoplasmic Reticulum] + Sbh1p[Endoplasmic Reticulum] + Sss1p[Endoplasmic Reticulum] <=> **SEC61C1**[Endoplasmic Reticulum] | Reversible | Co-translocation translocation | [***Sec61p***](http://www.yeastgenome.org/cgi-bin/locus.fpl?locus=sec61)***,*** [***Sbh1p***](http://www.yeastgenome.org/cgi-bin/locus.fpl?locus=sbh1) [***Sss1p***](http://www.yeastgenome.org/cgi-bin/locus.fpl?locus=sss1) | Sec61 complex or translocon comprised from 3 subunits including *Sec61p* (as major subunit), *Sbh1p* and *Sss1p*. SEC61C1 complex in yeast forms a channel for SRP-dependent protein import and retrograde transport of misfolded proteins out of the ER | **[**[**3**](#_ENREF_3)**,** [**4**](#_ENREF_4)**]** |
| 4 | SSH1C | **SSH1C** complex formation | Sbh1p[Endoplasmic Reticulum] + Sss1p[Endoplasmic Reticulum] + Ssh1p[Endoplasmic Reticulum] <= > **SSH1C**[Endoplasmic Reticulum] | Reversible | Post-translational translocation | [***Sbh1p***](http://www.yeastgenome.org/cgi-bin/locus.fpl?locus=sbh1)***,*** [***Sss1p***](http://www.yeastgenome.org/cgi-bin/locus.fpl?locus=sss1)***,*** [***Ssh1p***](http://www.yeastgenome.org/cgi-bin/locus.fpl?locus=ssh1) | *S. cerevisiae* contains a second Sec61-like complex involved in co-translational translocation called the Ssh1p complex | **[**[**5**](#_ENREF_5)**],[**[**4**](#_ENREF_4)**]** |
| 5 | SPC | SPC complex formation | Sec11p[Endoplasmic Reticulum] + Spc1p[Endoplasmic Reticulum] + Spc2p[Endoplasmic Reticulum] + Spc3p[Endoplasmic Reticulum] <=> **SPC**[Endoplasmic Reticulum] | Reversible | translocation from cytosol to ER | [***Sec11p***](http://www.yeastgenome.org/cgi-bin/locus.fpl?locus=sec11)***,*** [***Spc1p***](http://www.yeastgenome.org/cgi-bin/locus.fpl?locus=spc1) [***Spc2p***](http://www.yeastgenome.org/cgi-bin/locus.fpl?locus=spc2)***,***[***Spc3p***](http://www.yeastgenome.org/cgi-bin/locus.fpl?locus=spc3) | The yeast SPC (signal peptidase complex) comprises four proteins, Spc1p Spc2p, Spc3p, and Sec11p. SEC11 is essential for viability and for signal peptidase activity. | **[**[**2**](#_ENREF_2)**,** [**6**](#_ENREF_6)**]** |
| 6 | SEC63C | SEC63C complex formation | Sec71p[Endoplasmic Reticulum] + sec62p[Endoplasmic Reticulum] + sec63p[Endoplasmic Reticulum] + sec72p[Endoplasmic Reticulum] <=> **SEC63C**[Endoplasmic Reticulum] | Reversible | Post-translational translocation | [***Sec63p***](http://www.yeastgenome.org/cgi-bin/locus.fpl?locus=sec63)***,***[***Sec62p***](http://www.yeastgenome.org/cgi-bin/locus.fpl?locus=sec62)***,*** [***Sec66p***](http://www.yeastgenome.org/cgi-bin/locus.fpl?locus=sec66)***,*** [***Sec72p***](http://www.yeastgenome.org/cgi-bin/locus.fpl?locus=sec72) | Sec63 complex (Sec63p, Sec62p, Sec66p and Sec72p); with Sec61 complex, Kar2p/BiP and Lhs1p forms a channel competent for SRP-dependent and post-translational SRP-independent protein targeting and import into the ER | **[**[**7**](#_ENREF_7)**,** [**8**](#_ENREF_8)**]** |
| 7 | OSTC | OSTC complex formation | Ost1p[Endoplasmic Reticulum] + Ost2p[Endoplasmic Reticulum] + Ost3p[Endoplasmic Reticulum] + Ost4p[Endoplasmic Reticulum] + Ost5p[Endoplasmic Reticulum] + Ost6p[Endoplasmic Reticulum] + Stt3p[Endoplasmic Reticulum] + Swp1p[Endoplasmic Reticulum] + Wbp1p[Endoplasmic Reticulum] <= > **OSTC**[Endoplasmic Reticulum] | Reversible | **Protein N-glycosylation** | [***Ost1p***](http://www.yeastgenome.org/cgi-bin/locus.fpl?locus=ost1)***,*** [***Ost2p***](http://www.yeastgenome.org/cgi-bin/locus.fpl?locus=ost2)***,*** [***Ost3p***](http://www.yeastgenome.org/cgi-bin/locus.fpl?locus=ost3)***,*** [***Ost4p***](http://www.yeastgenome.org/cgi-bin/locus.fpl?locus=ost4)***,*** [***Ost5p***](http://www.yeastgenome.org/cgi-bin/locus.fpl?locus=ost5)***,*** [***Ost6p***](http://www.yeastgenome.org/cgi-bin/locus.fpl?locus=ost6)***,*** [***Stt3p***](http://www.yeastgenome.org/cgi-bin/locus.fpl?locus=stt3)***,*** [***Swp1p***](http://www.yeastgenome.org/cgi-bin/locus.fpl?locus=swp1)***,*** [***Wbp1p***](http://www.yeastgenome.org/cgi-bin/locus.fpl?locus=wbp1) | The OSTC complex contains nine protein subunits: Ost1p, Ost2p, Ost3p, Ost4p, Ost5p, Ost6p, Stt3p, Swp1p, and Wbp1p, all of which are integral membrane proteins of the ER. The OSTC interacts with the Sec61p pore complex involved in protein import into the ER.OSTC is responsible for N-linked glycosylation of the proteins. | **[**[**9**](#_ENREF_9)**]** |
| 8 | HRD1-HRD3C | HRD1-HRD3C  Complex formation | Hrd3p[Endoplasmic Reticulum] + Hrd1p[Endoplasmic Reticulum] + Usa1p[Endoplasmic Reticulum] + Der1p[Endoplasmic Reticulum] <=> **HRD1-HRD3C**  [Endoplasmic Reticulum] | Reversible | ERADL | [***Hrd3p***](http://www.yeastgenome.org/cgi-bin/locus.fpl?locus=hrd3)***,*** [***Hrd1p***](http://www.yeastgenome.org/cgi-bin/locus.fpl?locus=hrd1)***,*** [***Usa1p***](http://www.yeastgenome.org/cgi-bin/locus.fpl?locus=usa1)***,*** [***Der1p***](http://www.yeastgenome.org/cgi-bin/locus.fpl?locus=der1) | Hrd1-Hrd3C is a core membrane complex,  Consisting of the ubiquitin ligase Hrd1p and its cofactors Hrd3p, Der1p, and Usa1p. These components form a near stoichiometric complex that is more loosely associated with the luminal Yos9p and the cytosolic Cdc48  ATPase complex, whose membrane recruitment is facilitated by Ubx2p and plays a central role in ER-associated protein degradation (ERAD). | [[10](#_ENREF_10)] |
| 9 | AAAC | AAAC complex formation | Ubx2p[Endoplasmic Reticulum]+ Cdc48p[Cytoplasm] + Ufd1p[Cytoplasm] + Npl4p[Cytoplasm] <=> AAAC[Cytoplasm] | Reversible | ERADL | [***Ubx2p***](http://www.yeastgenome.org/cgi-bin/locus.fpl?locus=ubx2)***,*** [***Cdc48p***](http://www.yeastgenome.org/cgi-bin/locus.fpl?locus=cdc48)***,*** [***Ufd1p***](http://www.yeastgenome.org/cgi-bin/locus.fpl?locus=hfd1)***,*** [***Npl4p***](http://www.yeastgenome.org/cgi-bin/locus.fpl?locus=npl4) | AAAC involves in recognition of polyubiquitinated proteins and their presentation to the 26S proteasome for degradation; involved in transporting proteins from the ER to the cytosol. | [[10](#_ENREF_10), [11](#_ENREF_11)] |
| 10 | Sec23-Sec24C | Sec23-sec 24C  complex formation | Sec23p[COPII] + sec 24p[COPII] <=> Sec23-Sec 24C[COPII] | Reversible | COPII | [***Sec23p***](http://www.yeastgenome.org/cgi-bin/locus.fpl?locus=sec23)***,***[***Sec 24p***](http://www.yeastgenome.org/cgi-bin/locus.fpl?locus=sec24) | Sec23p-Sec24pC heterodimer of the COPII vesicle coat, required for cargo selection during vesicle formation in ER to Golgi transport. Sar1p GTPase activity stimulated by Sec23p and Sec24 has cargo selection role. | [[12-14](#_ENREF_12)] |
| 11 | Sar1-GTPase-cycle | Sar 1 GDP GTP exchange | Sec12p[Endoplasmic Reticulum] + sar1p-GDP[Cytoplasm] + GTP[Cytoplasm] => Sar1p-GTP[Endoplasmic Reticulum] + GDP[Endoplasmic Reticulum] + sec12p[Endoplasmic Reticulum] | irreversible | COPII | [***Sec12p***](sec12)***,*** [***Sar1p***](http://www.yeastgenome.org/cgi-bin/locus.fpl?locus=sar1) | Assembly of the COPII coat is initiated through activation of the small Ras-like GTPase Sar1. Sec12 is an ER-bound transmembrane GEF (guanine exchange factor) for Sar1 and it is an ER membrane protein which activates Sar1p by exchanging GDP for GTP. | [[15](#_ENREF_15), [16](#_ENREF_16)] |
| 12 | TRAPPIC | TRAPPI complex formation | 2 Bet3p[Cytoplasm] + Bet5p[Cytoplasm] + Trs20p[Cytoplasm] + Trs23p[Cytoplasm] + Trs31p[Cytoplasm]+ Trs33p[Cytoplasm] <=> TRAPPIC[COPII] | Irreversible | COPII[[17](#_ENREF_17)] | [***Bet3p***](http://www.yeastgenome.org/cgi-bin/locus.fpl?locus=bet3)***,***[***Bet5p***](http://www.yeastgenome.org/cgi-bin/locus.fpl?locus=bet5)***,***[***Trs20p***](http://www.yeastgenome.org/cgi-bin/locus.fpl?locus=trs20)***,***[***Trs23p***](http://www.yeastgenome.org/cgi-bin/locus.fpl?locus=trs23)***,***[***Trs31p***](http://www.yeastgenome.org/cgi-bin/locus.fpl?locus=trs31)[***Trs33p***](http://www.yeastgenome.org/cgi-bin/locus.fpl?locus=trs33) | In yeast, TRAPPI is a complex comprises seven small subunits (two  copies of Bet3p and one copy each of Bet5p, Trs20p, Trs23p,  Trs31p, and Trs33p) and mediates the tethering of endoplasmic reticulum (ER)-derived COPII-coated vesicles at the cis-Golgi. Except for Trs33p and Trs85p, all of the TRAPPI subunits are required for cell viability | [[18](#_ENREF_18), [19](#_ENREF_19)] |
| 13 | **AP1C** | AP1 coat formation and dissaciation | Apl4p[Cytoplasm] + Apl2p[Cytoplasm] +  Apm1p[Cytoplasm] + Aps1p[Cytoplasm] <=> **AP1C**[Cytoplasm] | Irreversible | CPY pathway, HDSV | [***Apl4p***](http://www.yeastgenome.org/cgi-bin/locus.fpl?locus=alp4)***,*** [***Apl2p***](http://www.yeastgenome.org/cgi-bin/locus.fpl?locus=alp2)  [***Apm1p***](http://www.yeastgenome.org/cgi-bin/locus.fpl?locus=apm1)***,***  [***Aps1p***](http://www.yeastgenome.org/cgi-bin/locus.fpl?locus=aps1) | The roles of the yeast AP-1 and AP-2 complexes (not shown) have not been firmly established. | [[20](#_ENREF_20)] |
| 14 | **AP2C** | AP2 coat formation and dissaciation | Apl3p[Cytoplasm] + Apl1p[Cytoplasm] + Apm4p[Cytoplasm] + Apsp2[Cytoplasm] <=> **AP2C**[Cytoplasm] | irreversible |  | [***Apl3p***](http://www.yeastgenome.org/cgi-bin/locus.fpl?locus=apl3)***,***  [***Apl1p***](http://www.yeastgenome.org/cgi-bin/locus.fpl?locus=apl1) ***,***[***Apm4p***](http://www.yeastgenome.org/cgi-bin/locus.fpl?locus=apm4) ***,*** [***Aps2p***](http://www.yeastgenome.org/cgi-bin/locus.fpl?locus=aps2) | The roles of the yeast AP-1 and AP-2 complexes (not shown) have not been firmly established. | [[20](#_ENREF_20)] |
| 15 | AP3C | AP3 coat formation and dissaciation | Apl6p[Cytoplasm] + Aps3p[Cytoplasm] + Apm3p[Cytoplasm] + Apl5p[Cytoplasm] + Vam3p[Vacuole] <=> **AP3C**[Cytoplasm] | Reversible | ALP pathway(AP-3 complex),  CPY Pathway | [***Apl6p***](http://www.yeastgenome.org/cgi-bin/locus.fpl?locus=apl6)***,*** [***Aps3p***](http://www.yeastgenome.org/cgi-bin/locus.fpl?locus=aps3)***,*** [***Apm3p***](http://www.yeastgenome.org/cgi-bin/locus.fpl?locus=apm3)***,***[***Apl5p***](http://www.yeastgenome.org/cgi-bin/locus.fpl?locus=apl5)***,***[***Vam3p***](http://www.yeastgenome.org/cgi-bin/locus.fpl?locus=vam3) | The AP-3 adaptor complex is essential for cargo-selective transport (alkaline phosphatase) to the yeast vacuole (ALP pathway) rather than identified AP-1 and AP-2 complex which mediate protein sorting at the trans-Golgi network and plasma membrane. | [[21](#_ENREF_21), [22](#_ENREF_22)] |
| 16 | clathrinC | Clathrin Complex formation | 2 Chc1p[Cytoplasm] + Clc1p[Cytoplasm] <=> clathrinC[Cytoplasm] | Reversible | CPY Pathway, HDSV, LDSV | [***Chc1p***](http://www.yeastgenome.org/cgi-bin/locus.fpl?locus=chc1)***,***[***Clc1p***](http://www.yeastgenome.org/cgi-bin/locus.fpl?locus=clc1) | Two Chc1p (heavy chain) form the Clathrin triskelion structural which is the major coat protein involved in intracellular protein transport and endocytosis. The Clc1p (the light chain) have a regulatory role. | [[23](#_ENREF_23), [24](#_ENREF_24)] |
| 17 | Sec13-Sec31C | Sec13-sec31C complex formation | Sec13p[COPII] + sec31p[COPII] <=> Sec13-sec31C[COPII] | reversible | COPII | [***Sec13p***](http://www.yeastgenome.org/cgi-bin/locus.fpl?locus=sec13)***,***[***Sec31p***](http://www.yeastgenome.org/cgi-bin/locus.fpl?locus=Sec31) | This complex is a subunits of the big complex called COPII vesicle coat complex needed for selection of the cargo and COPII vesicles to transport cargo from ER to Golgi. | [[25-27](#_ENREF_25)] |
| 18 | Arf1p-GTP | Arf1p-GTP formation | Arf1p-GDP[Cytoplasm] + Gea1p[Cytoplasm] + Gea2p[Cytoplasm] + 2 GTP[Cytoplasm] => Arf1p-GTP[Cytoplasm] + 2 GDP[Cytoplasm] + Gea1p[Cytoplasm] + Gea2p[Cytoplasm] | Irreversible | COPI | [***Arf1p***](http://www.yeastgenome.org/cgi-bin/locus.fpl?locus=Arf1)***,***[***Gea1p***](http://www.yeastgenome.org/cgi-bin/locus.fpl?locus=Gea1)***,***[***Gea2p***](http://www.yeastgenome.org/cgi-bin/locus.fpl?locus=Gea2) | Gea1p and Geap2p act as Arf1p ( ADP-ribosylation factor ) GEF( guanine nucleotide exchange factor ) and bound to the Golgi membrane and recruits the Arf1-GDP to bind and they convert it to the Arf1p-GTP which has essential role in COPI vesicle formation. | [[27-29](#_ENREF_27)] |
| 19 | COPIC | COPI coatomer | Cop1p[COPI] + Sec26p[COPI] + Sec27p[COPI] + Sec21p[COPI] + Ret2p[COPI] + Sec28p[COPI] + Ret3p[COPI] <=> COPIC[COPI] | reversible | COPI | [***Cop1p***](http://www.yeastgenome.org/cgi-bin/locus.fpl?locus=cop1)***,*** [***Sec26p***](http://www.yeastgenome.org/cgi-bin/locus.fpl?locus=sec26)***,*** [***Sec27p***](http://www.yeastgenome.org/cgi-bin/locus.fpl?locus=sec27)***,*** [***Sec21p***](http://www.yeastgenome.org/cgi-bin/locus.fpl?locus=sec21)***,*** [***Ret2p***](http://www.yeastgenome.org/cgi-bin/locus.fpl?locus=ret2)***,***[***Sec28p***](http://www.yeastgenome.org/cgi-bin/locus.fpl?locus=sec28)***,***[***Ret3p***](http://www.yeastgenome.org/cgi-bin/locus.fpl?locus=ret3) | COPI coatomer is a complex of seven protein which they assembled tighter and provide a coat structure for the COPI vesicles .It has been shown that Ret2p has role in cargo detection. COPI vesicles have a vital role in maintenance of the ER function and morphology. | [[30-35](#_ENREF_30)] |
| 20 | CPYIC | CPYIC complex formation | Pep12p[endosome] + Vps45p[endosome] <=> CPYIC[endosome] | reversible | CPY pathway | [***Pep12p***](http://www.yeastgenome.org/cgi-bin/locus.fpl?locus=pep12)***,*** [***Vps45p***](http://www.yeastgenome.org/cgi-bin/locus.fpl?locus=vps45) | Golgi-to-endosome transport along the carboxypeptidase Y (CPY) pathway requires Pep12p  (t-SNARE) and Vps45p (Sec1p homologue) | [[36-39](#_ENREF_36)] |
| 21 | CPYIIC | CPYIIC complex formation | Vps4p[endosome] + Vps27p[endosome] <=> CPYIIC[endosome] | reversible | CPY pathway | [***Vps4p***](http://www.yeastgenome.org/cgi-bin/locus.fpl?locus=vps4) ***,***[***Vps27p***](http://www.yeastgenome.org/cgi-bin/locus.fpl?locus=vps27) | endosome-to-vacuole transport along the CPY pathway requires Vps4p (AAA-type ATPase) and Vps27p(RING-finger protein) | [[40](#_ENREF_40), [41](#_ENREF_41)] |
| 22 | EXOC | LDSV exosytose | Sec3p[Cytoplasm] + Sec5p[Cytoplasm] + Sec6p[Cytoplasm] + Sec8p[Cytoplasm] + Sec10p[Cytoplasm] + Sec15p[Cytoplasm] + Exo70p[Cytoplasm] + Exo84p[Cytoplasm] + sec4p[Cytoplasm] <=> EXOC[Cytoplasm] | reversible | LDSV pathway | [***Sec3p***](http://www.yeastgenome.org/cgi-bin/locus.fpl?locus=sec3)***,*** [***Sec5p***](http://www.yeastgenome.org/cgi-bin/locus.fpl?locus=sec5)***,*** [***Sec6p***](http://www.yeastgenome.org/cgi-bin/locus.fpl?locus=sec6)***,*** [***Sec8p***](http://www.yeastgenome.org/cgi-bin/locus.fpl?locus=sec8)***,*** [***Sec10p***](http://www.yeastgenome.org/cgi-bin/locus.fpl?locus=sec10)***,*** [***Sec15p***](http://www.yeastgenome.org/cgi-bin/locus.fpl?locus=sec15)***,*** [***Exo70p***](http://www.yeastgenome.org/cgi-bin/locus.fpl?locus=exo70)***,*** [***Exo84p***](http://www.yeastgenome.org/cgi-bin/locus.fpl?locus=exo84)***,***[***Sec4p***](http://www.yeastgenome.org/cgi-bin/locus.fpl?locus=Sec4) | The Exocyst complex involve in targeting of the post-golgi vesicles to the exocytosis pathway. | [[42-45](#_ENREF_42)] |
| 23 | XXX-TC1 | signal peptide recognition | XXX[Cytoplasm] + **SRPC**[Cytoplasm] => XXX-**SRPC**[Cytoplasm] | irreversible | translocation from cytosol to ER (SRP-dependent) | ***SRPC(***[***Srp14p***](http://www.yeastgenome.org/cgi-bin/locus.fpl?locus=srp14)***,***[***Srp21p***](http://www.yeastgenome.org/cgi-bin/locus.fpl?locus=srp21)***,***[***Srp54p***](http://www.yeastgenome.org/cgi-bin/locus.fpl?locus=srp54)***,*** [***Srp65p***](http://www.yeastgenome.org/cgi-bin/locus.fpl?locus=Srp65)***,***[***Srp68p***](http://www.yeastgenome.org/cgi-bin/locus.fpl?locus=Srp68)***,***[***Srp72p***](http://www.yeastgenome.org/cgi-bin/locus.fpl?locus=Srp72)***,*** [***ScR1***](http://www.yeastgenome.org/cgi-bin/locus.fpl?locus=SCR1) ***RNA)*** | The SRPC (a ribonucleoprotein) is responsible to detect and bind to the signal peptide and target the protein to ER membrane. The formation of the SRPC and detecting of the SP is a sequential and complex stochastic process which starts in nucleus (by formation of core complex including the RNA subunit) and complete in cytoplasm by detecting the ribosome nascent chain complex (RNC) through the signal peptide. | [[46-48](#_ENREF_46)] |
| 24 | XXX-TC2 | ER receptor biding to XXX-SRPC | XXX-SRPC[Cytoplasm] + **SRC**[Endoplasmic Reticulum] => XXX-SRPC-SRC[Cytoplasm] | Irreversible | translocation from cytosol to ER (SRP-dependent) | ***SRPC(***[***Srp14p***](http://www.yeastgenome.org/cgi-bin/locus.fpl?locus=srp14)***,***[***Srp21p***](srp21)***,***[***Srp54p***](http://www.yeastgenome.org/cgi-bin/locus.fpl?locus=srp54)***,*** [***Srp65p***](http://www.yeastgenome.org/cgi-bin/locus.fpl?locus=srp65) [***Srp68p***](http://www.yeastgenome.org/cgi-bin/locus.fpl?locus=srp68)***,***[***scR1***](http://www.yeastgenome.org/cgi-bin/locus.fpl?locus=scr1)***RNA)***  ***SRC(***[***Srp101p***](http://www.yeastgenome.org/cgi-bin/locus.fpl?locus=srp101)***,*** [***Srp102p***](http://www.yeastgenome.org/cgi-bin/locus.fpl?locus=srp102)***)*** | The detected RNC (ribosome nascent chain) by SRPC binds to the to the SRP receptor (SR) on ER. | [[46-48](#_ENREF_46)] |
| 25 | XXX-TC3 | Biding of XXX-SRPC-SRC to the translocator(SEC61C) | XXX-SRPC-SRC[Cytoplasm] + 2 GTP[Cytoplasm] + **SEC61C**[Endoplasmic Reticulum] + **SSH1C**[Endoplasmic Reticulum] => XXX- SRPC-SRC-SEC61C[Cytoplasm] | Irreversible | translocation from cytosol to ER (SRP-dependent) | ***SRPC(***[***Srp14p***](http://www.yeastgenome.org/cgi-bin/locus.fpl?locus=srp14)***,***[***Srp21p***](http://www.yeastgenome.org/cgi-bin/locus.fpl?locus=srp21)***,***[***Srp54p***](http://www.yeastgenome.org/cgi-bin/locus.fpl?locus=srp54)***,*** [***Srp65p***](http://www.yeastgenome.org/cgi-bin/locus.fpl?locus=srp65)***,*** [***Srp68p***](http://www.yeastgenome.org/cgi-bin/locus.fpl?locus=Srp68)***,*** [***scR1***](http://www.yeastgenome.org/cgi-bin/locus.fpl?locus=scr1) ***RNA)***  ***SRC(***[***Srp101p***](http://www.yeastgenome.org/cgi-bin/locus.fpl?locus=srp101)***,***[***Srp102p***](http://www.yeastgenome.org/cgi-bin/locus.fpl?locus=srp102)***)***  ***SEC61C(***[***Sec61p***](http://www.yeastgenome.org/cgi-bin/locus.fpl?locus=sec61)***,***[***Sbh1p***](http://www.yeastgenome.org/cgi-bin/locus.fpl?locus=sbh1)***,***[***Sss1p***](sss1)***)Alternative SEC61C(***[***Sbh1p***](http://www.yeastgenome.org/cgi-bin/locus.fpl?locus=sbh1)***,***[***Sss1p***](http://www.yeastgenome.org/cgi-bin/locus.fpl?locus=sss1)***,***[***Ssh1p***](ssh1)***)*** | The Srp54p (subunits of SRP) and SR alpha (subunit of the SR) use a tightly coupled GTPase cycle to facilitates the signal sequence-dependent attachment of ribosomes to the SEC61C. This Cooperative binding stabilizes the SRP-SR complex and initiates the handing of signal sequence from Srp54p to Sec61 alpha. It has been shown that conformational change of the both SR alpha and Srp54p in binding to the GTP has distinct roles and SR alpha perform a predominant role in complex stabilization. | [[49-52](#_ENREF_49)] |
| 26 | XXX-TC4 | SRCPC and SRC dissociation | XXX-SRPC-SRC-SEC61C[Cytoplasm] **=>**  XXX-SEC61C[Cytoplasm] + Srp14p[Cytoplasm] + Srp21p[Cytoplasm] + Srp68p[Cytoplasm] + Sec65p[Cytoplasm] + Srp72p[Cytoplasm] + scR1[Cytoplasm] + Srp54p[Cytoplasm] + Srp51p[Cytoplasm] +  Srp52p[Cytoplasm] **+** 2GDP[Cytoplasm] + 2 pi[Cytoplasm] | Irreversible | translocation from cytosol to ER (SRP-dependent) | ***SRPC(***[***Srp14p***](http://www.yeastgenome.org/cgi-bin/locus.fpl?locus=srp14)***,***[***Srp21p***](http://www.yeastgenome.org/cgi-bin/locus.fpl?locus=srp21)***,***[***Srp54p***](http://www.yeastgenome.org/cgi-bin/locus.fpl?locus=srp54)***,*** [***Srp65p***](http://www.yeastgenome.org/cgi-bin/locus.fpl?locus=srp65)***,*** [***Srp68p***](http://www.yeastgenome.org/cgi-bin/locus.fpl?locus=Srp68)***,*** [***scR1***](http://www.yeastgenome.org/cgi-bin/locus.fpl?locus=scr1) ***RNA)***  ***SRC(***[***Srp101p***](http://www.yeastgenome.org/cgi-bin/locus.fpl?locus=srp101)***,***[***Srp102p***](http://www.yeastgenome.org/cgi-bin/locus.fpl?locus=srp102)***)*** | The dissociation of the SRP-SR complex cannot occur before the hydrolysis of bound GTP by both Srp54p and SR alpha.. | [[51](#_ENREF_51), [53](#_ENREF_53)] |
| 27 | XXX-TC5 | Signal peptidase | XXX-SEC61C[Cytoplasm] + **SPC**[Endoplasmic Reticulum] => XXX[Endoplasmic Reticulum] + Sec61p[Endoplasmic Reticulum] + Sbh1p[Endoplasmic Reticulum] + Sss1p[Endoplasmic Reticulum] + Sec11p[Endoplasmic Reticulum] + Spc1p[Endoplasmic Reticulum] + Spc2p[Endoplasmic Reticulum] + Spc3p[Endoplasmic Reticulum] + XXX**sp**[Cytoplasm] | Irreversible | translocation from cytosol to ER (SRP-dependent) | ***SEC61C(***[***Sec61p***](http://www.yeastgenome.org/cgi-bin/locus.fpl?locus=sec61)***,***[***Sbh1p***](http://www.yeastgenome.org/cgi-bin/locus.fpl?locus=sbh1)***,***[***Sss1p***](http://www.yeastgenome.org/cgi-bin/locus.fpl?locus=sss1)***)***  ***SPC(***[***Sec11p***](http://www.yeastgenome.org/cgi-bin/locus.fpl?locus=sec11)***,*** [***Spc1p***](http://www.yeastgenome.org/cgi-bin/locus.fpl?locus=spc1)***,*** [***Spc2p***](http://www.yeastgenome.org/cgi-bin/locus.fpl?locus=spc2)***,***[***Spc3p***](http://www.yeastgenome.org/cgi-bin/locus.fpl?locus=spc3)***)***  ***SSH1C (*** [***Sbh1p***](http://www.yeastgenome.org/cgi-bin/locus.fpl?locus=sbh1)***,*** [***Sss1p***](http://www.yeastgenome.org/cgi-bin/locus.fpl?locus=sss1)***,*** [***Ssh1p***](http://www.yeastgenome.org/cgi-bin/locus.fpl?locus=ssh1)***)*** | SPC (Signal peptidase complex) include the four subunits: Spc1p; Spc2p; Spc3p; Sec11p and is responsible for catalyzing the cleavage of signal peptides of proteins subjected to the secretory pathway. | [[2](#_ENREF_2)]; [[54-56](#_ENREF_54)] |
| 28 | SP-degradation | Signal peptide degradation | sp[Cytoplasm] => ac[Cytoplasm] | Irreversible | translocation from cytosol to ER |  | degradation of the signal peptide  **note:**(This reaction did not included in the model analysis and ignored due to its minor role) |  |
| 29 | XXX-PST1 | Post-translational translocation | XXX[Cytoplasm] + **SEC63C**[Endoplasmic Reticulum] + 5 Kar2p[Endoplasmic Reticulum] **+** 5 ATP[Endoplasmic Reticulum] + 5 Lhs1p[Endoplasmic Reticulum] <=> XXX-SEC63C-SEC61C-Kar2p-ATPcplx[Cytoplasm] | reversible | translocation from cytosol to ER (Post-translational translocation) | ***SEC63C (***[***Sec66p***](http://www.yeastgenome.org/cgi-bin/locus.fpl?locus=sec66)***,***[***Sec62p***](http://www.yeastgenome.org/cgi-bin/locus.fpl?locus=sec62)***,***[***Sec63p***](http://www.yeastgenome.org/cgi-bin/locus.fpl?locus=sec63)***,***[***Sec72p***](http://www.yeastgenome.org/cgi-bin/locus.fpl?locus=sec72) ***)SEC61C:(***[***Sec61***](http://www.yeastgenome.org/cgi-bin/locus.fpl?locus=sec61)***p,***[***Sbh1p***](http://www.yeastgenome.org/cgi-bin/locus.fpl?locus=sbh1)***,***[***Sss1p***](sss1)***)*** | SEC63C (Sec63p; Sec62p; Sec66p and Sec72p) with SEC61C, Kar2p/BiP and Lhs1p form two channel competent for SRP-dependent and post-translational SRP-independent protein targeting and import into the ER.  ATP and Kar2p are required for the insertion of precursor polypeptides into the Sec61C as well as for the completion of translocation into  the ER. Lhs1p only participates in post-translational import. | [[46](#_ENREF_46)]  [[57-59](#_ENREF_57)] |
| 30 | XXX-PSTII | Post-translational translocation | XXX-SEC63C-SEC61C-Kar2p-ATPcplx[Cytoplasm] => XXX[Endoplasmic Reticulum] + SSH1C[Endoplasmic Reticulum] + Sec61p[Endoplasmic Reticulum] + Sbh1p[Endoplasmic Reticulum] + Sss1p[Endoplasmic Reticulum] + 5 Kar2p[Endoplasmic Reticulum] +5 ADP[Endoplasmic Reticulum] + 5 Lhs1p[Endoplasmic Reticulum] | reversible | translocation from cytosol to ER (Post-translational translocation) | ***SEC63C(***[***Sec63p***](http://www.yeastgenome.org/cgi-bin/locus.fpl?locus=sec63)***,***[***Sec62p***](http://www.yeastgenome.org/cgi-bin/locus.fpl?locus=sec62)***,*** [***Sec66p***](http://www.yeastgenome.org/cgi-bin/locus.fpl?locus=sec66)***,*** [***Sec72p***](http://www.yeastgenome.org/cgi-bin/locus.fpl?locus=sec72) ***)SEC61C(***[***Sec61***](http://www.yeastgenome.org/cgi-bin/locus.fpl?locus=sec61)***p,***[***Sbh1p***](http://www.yeastgenome.org/cgi-bin/locus.fpl?locus=sbh1)***,***[***Sss1p***](http://www.yeastgenome.org/cgi-bin/locus.fpl?locus=sss1)***)*** | The SEC61C can interact  with the SEC63C which are involved in the BiP(Kar2p)-mediated  Post-translational translocation of preproteins across the ER membrane. |  |
| 31 | DOL-P-formation | Monophospho dolichol synthesis | Dol[Cytoplasm] + **CTP**[Cytoplasm] => Dol-p[Cytoplasm] + **CDP**[Cytoplasm] | Irreversible | synthesis of lipid linked precursor oligosaccharide | [***Sec59p***](http://www.yeastgenome.org/cgi-bin/locus.fpl?locus=sec59) | Dolichol kinase catalyzes the last step in dolichol monophosphate (Dol-P) biosynthesis by phosphorylation of the Carrier lipid Dol (polyisoprenoid) consuming one CTP. This enzyme is essential for viability and for normal rates of lipid intermediate synthesis, protein *N*-glycosylation,*O*-glycosylation and GPI anchor synthesis. | [[60-62](#_ENREF_60)] |
| 32 | Dol-PP-GlcNAcI-formation | Dol-PP-GlcNAc formation I | Dol-p[Cytoplasm] + **UDP-GlcNAc**[Cytoplasm] => Dol-pp-GlcNAc[Cytoplasm] + **UMP**[Cytoplasm] | Irreversible | synthesis of lipid linked precursor oligosaccharide | [***Alg7p***](http://www.yeastgenome.org/cgi-bin/locus.fpl?locus=alg7) | The biosynthesis of the oligosaccharide donor begins on the cytoplasmic face of the ER with Alg7; an N-acetylglucosamine-phosphate transferase that elaborates Dol-P to Dol-PP-GlcNAc. | [[63-65](#_ENREF_63)] |
| 33 | Dol-PP-GlcNAcII-formation | Dol-PP-GlcNAc formation II | Dol-pp-GlcNAc[Cytoplasm] + **UDP-GlcNAc**[Cytoplasm] => Dol-pp-GlcNAc(2)[Cytoplasm] + **UMP**[Cytoplasm] | Irreversible | synthesis of lipid linked precursor oligosaccharide | [***Alg13p***](http://www.yeastgenome.org/cgi-bin/locus.fpl?locus=alg13)***/14p*** | The addition of the second α1;4-GlcNAc residue is catalyzed by a hetero-oligomeric protein classified as Alg13/14. | [[66-68](#_ENREF_66)] |
| 34 | Dol-pp-GlcNAc-man-formationI | Dol-pp-GlcNAc(2) manosylation I | Dol-pp-GlcNAc(2)[Cytoplasm] + GDP-man[Cytoplasm] => Dol-pp-GlcNAc(2) -man[Cytoplasm] + GDP[Cytoplasm] | Irreversible | synthesis of lipid linked precursor oligosaccharide | [***Alg1p***](http://www.yeastgenome.org/cgi-bin/locus.fpl?locus=alg1) | Alg1p (beta 1;4 mannosyltransferase); catalyzes the addition of the first mannose moiety to the growing lipid-linked oligosaccharide (LLO).This reaction occurs on the cytosolic side of the endoplasmic reticulum. This enzyme is shown to form complex with Alg2p and Alg11p separately. The mutated strains are not able to add mannose to Dol-PP-GlcNAc2, but they can convert Man1-Dol-PP-GlcNAc2 to Man5-Dol-PP-GlcNAc2. | [[69-72](#_ENREF_69)] |
| 35 | Dol-pp- GlcNAc(2) -man-formationII | Dol-pp- GlcNAc(2) manosylation II | Dol-pp-GlcNAc(2) -man[Cytoplasm] + GDP-man[Cytoplasm] => Dol-pp-(GlcNAc)2(man)2[Cytoplasm] + UDP[Cytoplasm] | Irreversible | synthesis of lipid linked precursor oligosaccharide | [***Alg2p***](http://www.yeastgenome.org/cgi-bin/locus.fpl?locus=alg2) | Alg2p (mannosyltransferase) catalyzes the addition of both the second (via an alpha-1;3 linkage) and third ( via an alpha-1;6 linkage ) mannose moieties to the growing oligosaccharide chain during LLO biosynthesis. The mutants show accumulation of oligosaccharide chains (with one or two mannose residues). Alg2p form complexes with Alg1p. | [[73-75](#_ENREF_73)] |
| 36 | Dol-pp- GlcNAc(2) -man-formationIII | Dol-pp- GlcNAc(2) manosylation III | Dol-pp-(GlcNAc)2(man)2[Cytoplasm] + GDP-man[Cytoplasm] => Dol-pp-(GlcNAc)2(man)3[Cytoplasm] + GDP[Cytoplasm] | Irreversible | synthesis of lipid linked precursor oligosaccharide | [***Alg2p***](http://www.yeastgenome.org/cgi-bin/locus.fpl?locus=alg3) |  |  |
| 37 | Dol-pp- GlcNAc(2) -man-formationIV | Dol-pp- GlcNAc(2) manosylation IV | Dol-pp-(GlcNAc)2(man)3[Cytoplasm] + GDP-man[Cytoplasm] => Dol-pp-(GlcNAc)2(man)4[Cytoplasm] + GDP[Cytoplasm] | Irreversible | synthesis of lipid linked precursor oligosaccharide | [***Alg11p***](http://www.yeastgenome.org/cgi-bin/locus.fpl?locus=alg11) | Alg11p is responsible to add the fourth and fifth mannose to LLO on cytoplasmic side of the ER. It forms complexes together with Alg2p | [[76](#_ENREF_76), [77](#_ENREF_77)] |
| 38 | Dol-pp- GlcNAc(2) -man-formationV | Dol-pp- GlcNAc(2) manosylation V | Dol-pp-(GlcNAc)2(man)4[Cytoplasm] + GDP-man[Cytoplasm] => Dol-pp-(GlcNAc)2(man)5[Cytoplasm] + GDP[Cytoplasm] | Irreversible | synthesis of lipid linked precursor oligosaccharide | ***Alg11p*** |  |  |
| 39 | Dol-pp- GlcNAc(2) -man(5)-filliping | Dol-pp- GlcNAc(2) -man(5) filliping to the ER | Dol-pp-(GlcNAc)2(man)5[Cytoplasm] => Dol-pp-(GlcNAc)2(man)5[Endoplasmic Reticulum] | Irreversible | synthesis of lipid linked precursor oligosaccharide | [***Rft1p***](http://www.yeastgenome.org/cgi-bin/locus.fpl?locus=rft1) | The completely assembled heptasaccharide is flipped to the lumenal face of the ER membrane.by Rft1p, a transmembrane ATP-independent and bi-directional flippase. | [[78](#_ENREF_78)] |
| 40 | Dol-p-er-manosylation | ER manosylation of Dol-p | Dol-p[Cytoplasm] + **GDP-man**[Cytoplasm] => Dol-p-man[Cytoplasm] + **GDP**[Cytoplasm] | Irreversible | synthesis of lipid linked precursor oligosaccharide | [***Dpm1p***](http://www.yeastgenome.org/cgi-bin/locus.fpl?locus=dpm1) | Dpm1p (dolichyl phosphate mannose synthase, Dol-P-Man) (EC 2.4.1.83) adds a mannose moiety to dolichyl phosphate on the cytosolic side of the ER. Dol-P-Man flips into the lumen of the ER; where it serves as a source of mannose for three types of protein modifications: *N*-linked glycosylation; *O*-linked glycosylation; and synthesis of glycosyl phosphatidylinositol (GPI) anchors. Deletion of DPM1 is lethal. | [[79](#_ENREF_79), [80](#_ENREF_80)] |
| 41 | Dol-p-er-glycosylation | ER glycosylation of Dol-p | Dol-p[Cytoplasm] + **UDP-Clc**[Cytoplasm] => Dol-p-Glc[Cytoplasm] + **UDP**[Cytoplasm] | Irreversible | synthesis of lipid linked precursor oligosaccharide | [***Alg5p***](http://www.yeastgenome.org/cgi-bin/locus.fpl?locus=alg5) | Alg5p, a transmembrane dolichyl-phosphate beta-glucosyltransferase , adds glucose to dolichyl-phosphate (Dol-P) on the cytoplasmic side of the ER. By flipping Dol-P glucose enters into the lumen of the ER; where it serves as a source of glucose for growing lipid-linked oligosaccharides (LLO's). Mutation in ALG5 result in accumulation of LLO's (Man9) with no apparent growth defect. | [[81](#_ENREF_81)] |
| 42 | Dol-pp-GlcNAc(2)man(5)-er-manI | Dol-pp-GlcNAc(2)man(5) ER manosylation I | Dol-pp-GlcNAc(2)man(5)[Endoplasmic Reticulum] + **Dol-p-man**[Endoplasmic Reticulum] => Dol-pp-GlcNAc(2)man(6)[Endoplasmic Reticulum] + **Dol-p**[Endoplasmic Reticulum] | Irreversible | synthesis of lipid linked precursor oligosaccharide | [***Alg3p***](http://www.yeastgenome.org/cgi-bin/locus.fpl?locus=alg3) | Alg3p(alpha 1;3 mannosyltransferase) that adds the the sixth mannose moiety to the growing lipid-linked oligosaccharide (LLO),the first sugar added in the lumen of the ER.. Disruption of ALG3 causes accumulation of lipid-linked oligosaccharides with five mannose residues. | [[82](#_ENREF_82)] |
| 43 | Dol-pp-GlcNAc(2)man(5)-er-manII | Dol-pp-GlcNAc(2)man(5) ER manosylation II | Dol-pp-GlcNAc(2)man(6)[Endoplasmic Reticulum] + **Dol-p-man**[Endoplasmic Reticulum] => Dol-pp-GlcNAc(2)man(7)[Endoplasmic Reticulum] + **Dol-p**[Endoplasmic Reticulum] | Irreversible | synthesis of lipid linked precursor oligosaccharide | [***Alg9p***](http://www.yeastgenome.org/cgi-bin/locus.fpl?locus=alg9) | Alg9p (alpha 1;2 mannosyltransferase) catalyzes two steps: the addition of the seventh and ninth mannose moieties to the growing oligosaccharide in the lumen of the ER.. Disruption of ALG9 causes accumulation of lipid-linked oligosaccharides with six mannose residues and hypoglycosylation of secreted proteins. | [[83](#_ENREF_83)] |
| 44 | Dol-pp-GlcNAc(2)man(5)-er-manIII | Dol-pp-GlcNAc(2)man(5) ER manosylation III | Dol-pp-GlcNAc(2)man(7)[Endoplasmic Reticulum] + **Dol-p-man**[Endoplasmic Reticulum] => Dol-pp-GlcNAc(2)man(8)[Endoplasmic Reticulum] + **Dol-p**[Endoplasmic Reticulum] | Irreversible | synthesis of lipid linked precursor oligosaccharide | [***Alg12p***](http://www.yeastgenome.org/cgi-bin/locus.fpl?locus=alg12) | Alg12p (Alpha-1,6-mannosyltransferase) is an ER enzyme which adds the eighth mannose moieties .Mutation in ALG12 result in the accumulation of the lipid-linked oligosaccharides (LLO's) with seven mannose moieties. | [[84](#_ENREF_84)] |
| 45 | Dol-pp-GlcNAc(2)man(5)-er-manI | Dol-pp-GlcNAc(2)man(5) ER manosylation IV | Dol-pp-GlcNAc(2)man(8)[Endoplasmic Reticulum] + **Dol-p-man**[Endoplasmic Reticulum] => Dol-pp-GlcNAc(2)man(9)[Endoplasmic Reticulum] + **Dol-p**[Endoplasmic Reticulum] | Irreversible | synthesis of lipid linked precursor oligosaccharide | [***Alg9p***](http://www.yeastgenome.org/cgi-bin/locus.fpl?locus=alg9) | Alg9p (alpha 1,2 mannosyltransferase) shown to have a bi-functional nature. It catalyzes the addition of both the seventh and the ninth mannose residues (both α1;2-mannosyl linkages). | [[83](#_ENREF_83)] |
| 46 | Dol-pp-GlcNAc(2)man(9) - glcI | Dol-pp-GlcNAc(2)man(9) glycosylation I | Dol-pp-GlcNAc(2)man(9)[Endoplasmic Reticulum] + **Dol-p-Glc**[Endoplasmic Reticulum] => Dol-pp-GlcNAc(2)man(9)-Glc[Endoplasmic Reticulum] + **Dol-p**[Endoplasmic Reticulum] |  | synthesis of lipid linked precursor oligosaccharide | [***Alg6p***](http://www.yeastgenome.org/cgi-bin/locus.fpl?locus=alg6) | Alg6p( alpha 1;3 glucosyltransferase; catalyzes the addition of the first glucose to the growing lipid-linked oligosaccharide (LLO) in the lumen of the endoplasmic reticulum. | [[85](#_ENREF_85)] |
| 47 | Dol-pp-GlcNAc(2)man(9) - glcII | Dol-pp-GlcNAc(2)man(9) glycosylation II | Dol-pp-GlcNAc(2)man(9)-Glc[Endoplasmic Reticulum] + **Dol-p-Glc**[Endoplasmic Reticulum] => Dol-pp-GlcNAc(2)man(9)-Glc(2)[Endoplasmic Reticulum] + **Dol-p**[Endoplasmic Reticulum] | Irreversible | synthesis of lipid linked precursor oligosaccharide | [***Alg8p***](http://www.yeastgenome.org/cgi-bin/locus.fpl?locus=alg8) | Alg8p (alpha 1;3 glucosyltransferase ) an ER-membrane-bound adds of the second of three glucose moieties to growing LLO's in the lumen of ER. Mutants lacking ALG8 produce truncated LLO's with only one glucose residue that are able to be transferred to proteins with reduced efficiency. | [[86](#_ENREF_86)] |
| 48 | Dol-pp-GlcNAc(2)man(9) - glcIII | Dol-pp-GlcNAc(2)man(9) glycosylation III | Dol-pp-GlcNAc(2)man(9)-Glc(2)[Endoplasmic Reticulum] + **Dol-p-Glc**[Endoplasmic Reticulum] => Dol-pp-GlcNAc(2)man(9)-Glc(3)[Endoplasmic Reticulum] + **Dol-p**[Endoplasmic Reticulum] | Irreversible | synthesis of lipid linked precursor oligosaccharide | [***Die2p***](http://www.yeastgenome.org/cgi-bin/locus.fpl?locus=die2) | Die2p (alpha-1;2 glucosyltransferase) catalyzes the addition of the third(The final sugar to add to LLO). | [[87](#_ENREF_87)] |
| 49 | GPIS1C |  | Gpi1p[Cytoplasm] + Gpi2p[Cytoplasm] + Spt14p[Cytoplasm] + Gpi15p[Cytoplasm] + Gpi19p[Cytoplasm] + Eri1p[Cytoplasm] <=> GPIS1C[Cytoplasm] | reversible | GPI biosynthesis | [***Gpi1p***](http://www.yeastgenome.org/cgi-bin/locus.fpl?locus=gpi1)***,*** [***Gpi2p***](http://www.yeastgenome.org/cgi-bin/locus.fpl?locus=gpi2)***,*** [***Spt14p***](http://www.yeastgenome.org/cgi-bin/locus.fpl?locus=spt14)***,***[***Gpi15p***](http://www.yeastgenome.org/cgi-bin/locus.fpl?locus=gpi15)***,***[***Gpi19p***](http://www.yeastgenome.org/cgi-bin/locus.fpl?locus=gpi19)***,***[***Eri1p***](http://www.yeastgenome.org/cgi-bin/locus.fpl?locus=eri1) | The complex enzyme N-Acetyle-Glucoseamine-transferase comprised of 6 ER integrated membrane proteins. | [[88](#_ENREF_88), [89](#_ENREF_89)] |
| 50 | GPIB1 |  | PI[Cytoplasm] + UDP-GlcNAc[Cytoplasm] => PI-GlcNAc[Cytoplasm] + UDP[Cytoplasm] + Gpi1p[Cytoplasm] + Gpi2p[Cytoplasm] + Spt14p[Cytoplasm] + Gpi15p[Cytoplasm] + Gpi19p[Cytoplasm] + Eri1p[Cytoplasm] | Irreversible | GPI biosynthesis | [***Gpi1p***](http://www.yeastgenome.org/cgi-bin/locus.fpl?locus=gpi1)***,*** [***Gpi2p***](http://www.yeastgenome.org/cgi-bin/locus.fpl?locus=gpi2)***,*** [***Spt14p***](http://www.yeastgenome.org/cgi-bin/locus.fpl?locus=spt14)***,***[***Gpi15p***](http://www.yeastgenome.org/cgi-bin/locus.fpl?locus=gpi15)***,***[***Gpi19p***](http://www.yeastgenome.org/cgi-bin/locus.fpl?locus=gpi19)***,***[***Eri1p***](http://www.yeastgenome.org/cgi-bin/locus.fpl?locus=eri1) | N-Acetyle-Glucoseamine-transferase transfers GlcNAc consuming UDP-GlcNAc( donor) to the PI(phosphatidyle inositol) as the first step of the GPI biosynthesis. This reaction occurs in the cytoplasmic side of the ER. | [[88](#_ENREF_88), [89](#_ENREF_89)] |
| 51 | GPIB2 |  | PI-GlcNAc[Cytoplasm] => PI-GlcN[Cytoplasm] + Acetate[Cytoplasm] | Irreversible | GPI biosynthesis | [***Gpi12p***](http://www.yeastgenome.org/cgi-bin/locus.fpl?locus=gpi12) | Gpi12p de-*N*-acetylated the Glc-NAc-PI in the first step. | [[89](#_ENREF_89), [90](#_ENREF_90)] |
| 52 | GPIB3 |  | PI-GlcN[Cytoplasm]=> PI-GlcN[Endoplasmic Reticulum] | Irreversible | GPI biosynthesis |  | In second step Glc-N is flipped to the ER side for passing through future reaction |  |
| 53 | GPIB4 |  | PI-GlcN[Endoplasmic Reticulum] + Palmitoyl-CoA[Endoplasmic Reticulum] => Acyl-PI-GlcN[Endoplasmic Reticulum] + CoA[Endoplasmic Reticulum] | Irreversible | GPI biosynthesis | [***Gwt1p***](http://www.yeastgenome.org/cgi-bin/locus.fpl?locus=gwt1) | In the fourth step in ER, PI-Glc-N IS inositol-acylated by Gw1p as enzyme. | [[89](#_ENREF_89), [91](#_ENREF_91)] |
| 54 | GPIB5 |  | Acyl-PI-GlcN[Endoplasmic Reticulum] + Dol-p-man[Endoplasmic Reticulum] => Acyl-PI-GlcN-man[Endoplasmic Reticulum] + Dol-p[Endoplasmic Reticulum] | Irreversible | GPI biosynthesis | [***Gpi14p***](http://www.yeastgenome.org/cgi-bin/locus.fpl?locus=gpi14) | In the fifth step,the first mannose is added to the Acyl-PI-GlcN. | [[89](#_ENREF_89), [92](#_ENREF_92)] |
| 55 | GPIB6 |  | Acyl-PI-GlcN-man[Endoplasmic Reticulum] + EtNP[Endoplasmic Reticulum] => Acyl-PI-GlcN-man-EtNP[Endoplasmic Reticulum] | Irreversible | GPI biosynthesis | [***Mcd4p***](http://www.yeastgenome.org/cgi-bin/locus.fpl?locus=mcd4) | In the sixth step the phosphorylethanolamine is added to Man1 | [[89](#_ENREF_89), [93](#_ENREF_93)] |
| 56 | GPIB7 |  | Acyl-PI-GlcN-man-EtNP[Endoplasmic Reticulum] + Dol-p-man[Endoplasmic Reticulum] => Acyl-PI-GlcN-(man)2-EtNP[Endoplasmic Reticulum] + Dol-p[Endoplasmic Reticulum] | Irreversible | GPI biosynthesis | [***Gpi5p***](http://www.yeastgenome.org/cgi-bin/locus.fpl?locus=gpi5) | In the seventh step the second mannose is added to the Acyl-PI-GlcN. | [[89](#_ENREF_89), [94](#_ENREF_94)] |
| 57 | GPIB8 |  | Acyl-PI-GlcN-(man)2-EtNP[Endoplasmic Reticulum] + Dol-p-man[Endoplasmic Reticulum] => Acyl-PI-GlcN-(man)3-EtNP[Endoplasmic Reticulum] + Dol-p[Endoplasmic Reticulum] | Irreversible | GPI biosynthesis | [***Gpi10p***](http://www.yeastgenome.org/cgi-bin/locus.fpl?locus=gpi10) | In eighth step the third mannose is added to the Acyl-PI-GlcN. | [[89](#_ENREF_89), [95](#_ENREF_95)] |
| 58 | GPIB9 |  | Acyl-PI-GlcN-(man)3-EtNP[Endoplasmic Reticulum] + Dol-p-man[Endoplasmic Reticulum] => Acyl-PI-GlcN-(man)4-EtNP[Endoplasmic Reticulum] + Dol-p[Endoplasmic Reticulum] | Irreversible | GPI biosynthesis | [***Smp3p***](http://www.yeastgenome.org/cgi-bin/locus.fpl?locus=smp3) | In ninth step, the forth mannose is added to the Acyl-PI-GlcN. | [[89](#_ENREF_89), [96](#_ENREF_96)] |
| 59 | GPIB10 |  | Acyl-PI-GlcN-(man)4-EtNP[Endoplasmic Reticulum] + EtNP[Endoplasmic Reticulum] => Acyl-PI-GlcN-(man)4-(EtNP)2[Endoplasmic Reticulum] | Irreversible | GPI biosynthesis | [***Gpi13p***](http://www.yeastgenome.org/cgi-bin/locus.fpl?locus=gpi13) | In tenth step, second phosphophorylethanolamine is added to Man3 | [[89](#_ENREF_89), [97](#_ENREF_97)] |
| 60 | GPIB11 |  | Acyl-PI-GlcN-(man)4-(EtNP)2[Endoplasmic Reticulum]+ EtNP[Endoplasmic Reticulum] => Acyl-PI-GlcN-(man)4-(EtNP)3[Endoplasmic Reticulum] | Irreversible | GPI biosynthesis | [***Gpi17p***](http://www.yeastgenome.org/cgi-bin/locus.fpl?locus=gpi17) | In eleventh step, second phosphophorylethanolamine is added to Man2 | [[89](#_ENREF_89), [98](#_ENREF_98)] |
| 61 | XXX-GPIR1 |  | XXX-G5[Endoplasmic Reticulum] + Acyl-PI-GlcN-(man)4-(EtNP)3[Endoplasmic Reticulum] => XXX-G6[Endoplasmic Reticulum] | Irreversible | GPI transfer | [***Gaa1p***](http://www.yeastgenome.org/cgi-bin/locus.fpl?locus=gaa1)***,***[***Gpi8p***](http://www.yeastgenome.org/cgi-bin/locus.fpl?locus=gpi8)***,***[***Gpi16p***](http://www.yeastgenome.org/cgi-bin/locus.fpl?locus=gpi16)***,***[***Gpi17p***](http://www.yeastgenome.org/cgi-bin/locus.fpl?locus=gpi17)***,***[***Gab1p***](http://www.yeastgenome.org/cgi-bin/locus.fpl?locus=gab1) | GPI-Transaminase (complex enzyme) transfers the GPI to the GPI site on the proteins. | [[89](#_ENREF_89), [99](#_ENREF_99)] |
| 62 | XXX-GPIR2 |  | XXX-G6[Endoplasmic Reticulum] => XXX-G6[Endoplasmic Reticulum] + acyl[Endoplasmic Reticulum] | Irreversible | GPI transfer | [***Bst1p***](http://www.yeastgenome.org/cgi-bin/locus.fpl?locus=bst1) | In this reaction the acyle is removed from the inositol after the GPI has been added to protein. | [[89](#_ENREF_89), [100](#_ENREF_100)] |
| 63 | XXX -ERNG | N-linked glycosylation | XXX-SEC61C[Endoplasmic Reticulum] + ?Dol-pp-GlcNAc(2)man(9)-Glc(3)[Endoplasmic Reticulum] + OSTC[[Endoplasmic Reticulum]] => XXX-G1-SEC61C[Endoplasmic Reticulum] + ?Dol-pp[Endoplasmic Reticulum] + Ost1p[Endoplasmic Reticulum] + Ost2p[Endoplasmic Reticulum] + st3p[Endoplasmic Reticulum] + Ost4p[Endoplasmic Reticulum] + Ost5p[Endoplasmic Reticulum] + Ost6p[Endoplasmic Reticulum] + Stt3p[Endoplasmic Reticulum] + Swp1p[Endoplasmic Reticulum] + Wbp1p[Endoplasmic Reticulum] | Irreversible | **Protein N-glycosylation** | ***OSTC(***[***Stt3p***](http://www.yeastgenome.org/cgi-bin/locus.fpl?locus=stt3)***,***[***Ost1***](http://www.yeastgenome.org/cgi-bin/locus.fpl?locus=ost1)***p,***[***Wbp1p***](http://www.yeastgenome.org/cgi-bin/locus.fpl?locus=wbp1)***,***[***Ost3p***](http://www.yeastgenome.org/cgi-bin/locus.fpl?locus=ost3)***,***[***Ost6p***](http://www.yeastgenome.org/cgi-bin/locus.fpl?locus=ost6)***,***[***Swp1p***](http://www.yeastgenome.org/cgi-bin/locus.fpl?locus=swp1)***,***[***Ost2p***](http://www.yeastgenome.org/cgi-bin/locus.fpl?locus=ost2)***,***[***Ost5p***](http://www.yeastgenome.org/cgi-bin/locus.fpl?locus=ost5)***,***[***Ost4p***](http://www.yeastgenome.org/cgi-bin/locus.fpl?locus=ost4)***)*** | The oligosaccharyl transferase complex (OST complex) (EC 2.4.1.119) transfers 14-sugar branched oligosaccharides from dolichyl pyrophosphate to asparagine residues. The complex contains nine protein subunits: Ost1p( alpha subunit); Ost2p( epsilon subunit); Ost3p; Ost4p; Ost5p; Ost6p; Stt3p; Swp1p( delta subunit); and Wbp1p; all of which are integral membrane proteins of the. Genetic-knockout experiments have revealed that five of these subunits (Ost2p; Ost1p; Stt3p; Swp1p; and Wbp1p) are absolutely essential for yeast viability | [[101-106](#_ENREF_101)] |
| 64 | XXX -EROG | Er O-linked glycosylation | XXX-SEC61C[Endoplasmic Reticulum] + Dol-pp-Man[Endoplasmic Reticulum] => XXX-G1-SEC61C[Endoplasmic Reticulum] + Dol-pp[Endoplasmic Reticulum] |  | **O-Glycosylation** | ***PMTC(***[***Pmt2p***](http://www.yeastgenome.org/cgi-bin/locus.fpl?locus=pmt2)***,***[***Pmt5p***](http://www.yeastgenome.org/cgi-bin/locus.fpl?locus=pmt5)***,***[***Pmt1p***](http://www.yeastgenome.org/cgi-bin/locus.fpl?locus=pmt1)***,***[***Pmt6p***](http://www.yeastgenome.org/cgi-bin/locus.fpl?locus=pmt6)***,***[***Pmt4p***](http://www.yeastgenome.org/cgi-bin/locus.fpl?locus=pmt4)***,*** [***Pmt3p***](http://www.yeastgenome.org/cgi-bin/locus.fpl?locus=pmt3)***)*** | PMTC, protein *O*-mannosyltransferase, transfers mannose residues from dolichyl phosphate-D-mannose to protein Ser/Thr residues; it has been shown it also involved in ER quality control. | [[107-109](#_ENREF_107)] |
| 65 | XXX-ERGB |  | XXX_SEC61C[Endoplasmic Reticulum] + ?Dol-pp_Man[Endoplasmic Reticulum] + ?Dol-pp_GlcNAc(2)man(9)_Glc(3)[Endoplasmic Reticulum] => XXX_G1_SEC61C[Endoplasmic Reticulum] + ?Dol-pp[Endoplasmic Reticulum] |  | **O and N-Glycosylation** |  | This reaction stands fort he protiens which they have both *N* and *O*-linked glycosylation sites to get gylcan chain. |  |
| 66 | XXX-FL1 | SEC61C dissociation | XXX-G1-SEC61C[Endoplasmic Reticulum] => XXX-G1[Endoplasmic Reticulum] + SEC61C[Endoplasmic Reticulum] | Irreversible | protein folding |  | After getting the glycan chain and cleavage of the signal peptide the protein disassociated from SEC61C. |  |
| 67 | XXX-FL2 | ER Glycan trimming I | XXX-G1[Endoplasmic Reticulum] => XXX-G2[Endoplasmic Reticulum] + Glc[Endoplasmic Reticulum] | Irreversible | protein folding | [***Cwh41p***](http://www.yeastgenome.org/cgi-bin/locus.fpl?locus=cwh41) | Cwh41p (glucosidase I, Gls1) removes the most distal glucose from N-linked oligosaccharides ( Glc_3_-Man_9_-GlcNAc_2_).It is a type II ER transmembrane protein. | [[110](#_ENREF_110)] |
| 68 | XXX-FL3 | ER Glycan trimming II | XXX-G2[Endoplasmic Reticulum] => XXX-G3[Endoplasmic Reticulum] + Glc[Endoplasmic Reticulum] | Irreversible | protein folding | [***Rot2p***](http://www.yeastgenome.org/cgi-bin/locus.fpl?locus=rot2) | Rot2p ( glucosidase II ,GLS2) is a lumenal enzyme of the ER. It trims the two remaining glucose moieties previously added by Alg8p and Alg6p.  While Mutants lacking Rot2p do not have growth defect but cell wall biosynthesis and degradation of misfolded proteins have shown to be affected. | [[111](#_ENREF_111)] |
| 69 | XXX- FL3.1 | ER Glycan trimming III | XXX-G3[Endoplasmic Reticulum] => XXX-G4[Endoplasmic Reticulum] + Glc[Endoplasmic Reticulum] | irreversible | protein folding | [***Rot2p***](http://www.yeastgenome.org/cgi-bin/locus.fpl?locus=rot2) | Elemination of the last glucose from the glycan chain. | [[111](#_ENREF_111)] |
| 70 | XXX- FL3.2 | ER demanosylation I | XXX-G4[Endoplasmic Reticulum] => XXX-G5[Endoplasmic Reticulum] + man[Endoplasmic Reticulum] | irreversible | protein folding | [***Mns1p***](http://www.yeastgenome.org/cgi-bin/locus.fpl?locus=mns1) | Mns1p, type II ER membrane protein ,removes one of the mannose residues added by Alg9p from N-linked core oligosaccharides. This is the last trimming reaction that occurs in the ER before maturing proteins migrate to the Golgi apparatus. | [[112](#_ENREF_112), [113](#_ENREF_113)] |
| 71 | XXX- FL4 | Formation of folding complex with Kar2ATP and disulfide bond formation | XXX-G5[Endoplasmic Reticulum] + 5 Kar2p-ATP[Endoplasmic Reticulum]+ 5 Scj1p[Endoplasmic Reticulum] + 5 Jem1p[Endoplasmic Reticulum] + 5 Sec63p[Endoplasmic Reticulum] => XXX-G5-Kar2ATPcplx[Endoplasmic Reticulum] | reversible | protein folding | [***Kar2p***](http://www.yeastgenome.org/cgi-bin/locus.fpl?locus=kar2)***,***[***Scj1p***](http://www.yeastgenome.org/cgi-bin/locus.fpl?locus=scj1)***,***[***Jem1p***](http://www.yeastgenome.org/cgi-bin/locus.fpl?locus=jem1)***,*** [***Sec63p***](http://www.yeastgenome.org/cgi-bin/locus.fpl?locus=sec63) | Kar2p in the lumen of the ER is an essential protein that is a member of the HSP70 family of molecular chaperones which has the main role in secretory protein folding and translocation. Sec63p; Scj1p; and Jem1p ( DnaJ/HSP40 protein chaperone family) are cochaperones that enhance the ATPase activity of Kar2p. | [[114-120](#_ENREF_114)] |
| 72 | XXX - FL5 | XXX -Sulfation | XXX-G5-Kar2ATPcplx[Endoplasmic Reticulum] + Pdi1p[Endoplasmic Reticulum] + Ero1p[Endoplasmic Reticulum] + O2[Endoplasmic Reticulum] + FADH[Endoplasmic Reticulum] + Erv2p[Endoplasmic Reticulum] => XXX-S-G5-Kar2ATPcplx[Endoplasmic Reticulum] + H2O[Endoplasmic Reticulum] + FAD[Endoplasmic Reticulum] + h[Endoplasmic Reticulum] | irreversible | protein folding | [***Pdi1p***](http://www.yeastgenome.org/cgi-bin/locus.fpl?locus=pdi1)***,*** [***Ero1p***](http://www.yeastgenome.org/cgi-bin/locus.fpl?locus=ero1)***,***[***Erv2p***](http://www.yeastgenome.org/cgi-bin/locus.fpl?locus=erv2) | Pdi1p is a well characterized enzyme in ER lumen responsible for disulfide bound formation. It is a member of protein disulfide isomerase (PDI family).Both Ero1p and Erv2p have shown to have disulfide isomerase activity with supportive role for Pdi1p activity. | [[116](#_ENREF_116), [121-123](#_ENREF_121)] |
| 73 | XXX – FL6 |  | XXX-G5-Kar2ATPcplx-[Endoplasmic Reticulum] => XXX-F-G5-Kar2ADPcplx[Endoplasmic Reticulum] + 5 Scj1p[Endoplasmic Reticulum] + 5 Jem1p[Endoplasmic Reticulum] + 5 pi[Endoplasmic Reticulum] + Sec63p[Endoplasmic Reticulum] | irreversible | protein folding |  |  |  |
| 74 | XXX- FL7 | Kar2-ADP complex ATP exchange | XXX-S-G5-Kar2ATPcplx-[Endoplasmic Reticulum] => XXX-F-G5-Kar2ADPcplx[Endoplasmic Reticulum] + 5 Scj1p[Endoplasmic Reticulum] + 5 Jem1p[Endoplasmic Reticulum] + 5 pi[Endoplasmic Reticulum] + Sec63p[Endoplasmic Reticulum] + Pdi1p[Endoplasmic Reticulum] + Ero1p[Endoplasmic Reticulum] + Erv2p[Endoplasmic Reticulum] | irreversible | protein folding | [***Scj1p***](http://www.yeastgenome.org/cgi-bin/locus.fpl?locus=scj1)***,*** [***Jem1p***](jem1)***,*** [***Sec63p***](sec63) |  |  |
| 75 | XXX-FL8 | Folding complex dissociation | XXX-F-G5-Kar2-ADPcplx[Endoplasmic Reticulum] + 5 Sil1p[Endoplasmic Reticulum] + 5 Lhs1p[Endoplasmic Reticulum] + 5 ATP[Endoplasmic Reticulum] => XXX-F-G5[Endoplasmic Reticulum] + 5 Sil1p[Endoplasmic Reticulum] + 5 Lhs1p[Endoplasmic Reticulum] + 5 ADP[Endoplasmic Reticulum] + 5 Kar2p-ATP[Endoplasmic Reticulum] | irreversible | protein folding | [***Kar2p***](http://www.yeastgenome.org/cgi-bin/locus.fpl?locus=kar2p)***,***[***Lhs1p***](http://www.yeastgenome.org/cgi-bin/locus.fpl?locus=lhs1)***,***[***Sil1p***](http://www.yeastgenome.org/cgi-bin/locus.fpl?locus=sil1) | Sil1p and Lhs1p promote the subsequent ADP-ATP exchange of the Kar2p. Lhs1p activity affects the refolding and stability of heat-denatured proteins.Sil1p is the main NEFs for Kar2p. | [[116](#_ENREF_116)] |
| 76 | XXX-ERGL1 | Pre budding complex forming for soluble proteins | XXX-F-G5[Endoplasmic Reticulum] + Sar1-GTP[Endoplasmic Reticulum] + sec23-sec24C[Cytoplasm] + Bet1p[Endoplasmic Reticulum] + Bos1p[Endoplasmic Reticulum]=> XXX-F-G5-COPII-pbud-cplx[Endoplasmic Reticulum] | irreversible | COPII (for transmembrane cargo) | [***Sar1p***](http://www.yeastgenome.org/cgi-bin/locus.fpl?locus=sar1),[***Sec23p***](sec23),[***Sec24p***](http://www.yeastgenome.org/cgi-bin/locus.fpl?locus=sec24),[***Bet1p***](http://www.yeastgenome.org/cgi-bin/locus.fpl?locus=bet1),[***Bos1p***](http://www.yeastgenome.org/cgi-bin/locus.fpl?locus=bos1) | The COPII coated formation and cargo detection component for transmembrane cargo which has been shown to use some different component from the soluble or GPI-anchored proteins.  Sar1p,Sec23p and Sec24p are the common protein among the different cargo selection and coat formation mechanism. | [[69](#_ENREF_69)-72] |
| 77 | XXX-ERGL2 | Pre budding complex forming for soluble proteins | XXX-F-G5[Endoplasmic Reticulum] + Sar1-GTP[Endoplasmic Reticulum] + sec23-sec24C[Cytoplasm] + Erv29p[Endoplasmic Reticulum] + Bet1p[Endoplasmic Reticulum] + Bos1p[Endoplasmic Reticulum]=> XXX-F-G5-COPII-pbud-cplx[Endoplasmic Reticulum] | irreversible | COPII(for soluble cargo) | [***Sar1p***](http://www.yeastgenome.org/cgi-bin/locus.fpl?locus=sar1)***,***[***Sec23p***](http://www.yeastgenome.org/cgi-bin/locus.fpl?locus=sec23)***,***[***Sec24p***](http://www.yeastgenome.org/cgi-bin/locus.fpl?locus=Sec24)***,***[***Erv29p***](Erv29) | selective export of soluble luminal cargo would require specific transmembrane cargo receptors.Yeast Erv29p has been shown to be required for efficient packaging of the glycosylated alpha factor pheromone precursor (gpaf) into COPII vesicles and for efficient secretion of arboxypeptidase Y (CPY) | [[69](#_ENREF_69)-72] |
| 78 | XXX-ERGL3 | Pre budding complex forming for GPI-anchored proteins | XXX-7-G5[Endoplasmic Reticulum] + Sar1-GTP[Endoplasmic Reticulum] + Sec23-Sec24C[Cytoplasm] + Emp24p[Endoplasmic Reticulum] + Bet1p[Endoplasmic Reticulum] + Bos1p[Endoplasmic Reticulum] => XXX-F-G7-COPII-pbud-cplx[Endoplasmic Reticulum] | irreversible | COPII(for GPI-anchored cargo) | [***Sar1p***](http://www.yeastgenome.org/cgi-bin/locus.fpl?locus=sar1)***,***[***Sec23p***](http://www.yeastgenome.org/cgi-bin/locus.fpl?locus=sec23)***,***[***Sec24p***](http://www.yeastgenome.org/cgi-bin/locus.fpl?locus=sec24)***,*** [***Emp24p***](http://www.yeastgenome.org/cgi-bin/locus.fpl?locus=emp24) | GPI-anchored proteins also need adaptor for cargo selection like soluble proteins (as they do not have cytoplasmic tail).But according to the experimental evidence they use different kind of transmembrane adaptors than soluble proteins. Emp24 which is from yeast p24 family proteins is one of these studied proteins which have shown to work as adaptor for efficient transport of GPI-anchored cargo. | [[69](#_ENREF_69)-[[124](#_ENREF_124)]72] |
| 79 | XXX- ERGL4 | COPII formation | XXX-F-G7orG5-COPII-pbud-cplx[Endoplasmic Reticulum] + sec13-sec31C[Cytoplasm] + sec16p[Endoplasmic Reticulum] + sed4p[Endoplasmic Reticulum] + Sec5p[Endoplasmic Reticulum] + Sec17p[Endoplasmic Reticulum] => XXX-F-G5orG7-COPII[COPII] | Irreversible | COPII | [***Sec13p***](http://www.yeastgenome.org/cgi-bin/locus.fpl?locus=sec13)***,***[***Sec31p***](http://www.yeastgenome.org/cgi-bin/locus.fpl?locus=sec31)***,***[***Sec16p***](http://www.yeastgenome.org/cgi-bin/locus.fpl?locus=sec16)***,***[***Sed4p***](http://www.yeastgenome.org/cgi-bin/locus.fpl?locus=sed4)***,*** [***Sec5p***](http://www.yeastgenome.org/cgi-bin/locus.fpl?locus=sec5)***,*** [***Sec17p***](http://www.yeastgenome.org/cgi-bin/locus.fpl?locus=sec17) | prebudding complex  recruits Sec13-Sec31C heterotetramer providing the outer layer of the coat.Although Sar1p; Sec23p; Sec24p; Sec13p; and Sec31p are necessary and sufficient for vesicle formation; additional factors such as Sec16p and Sed4p are also involved in this process. Through interactions with other COPII proteins; Sec16p is thought to facilitate the assembly of the vesicle coat by stabilizing the pre-budding complex while Sed4p may regulate the vesicle budding process by inhibiting the GAP activity of Sec23p. | [[125-127](#_ENREF_125)] |
| 80 | XXX- ERGL5 | COPII fusion | XXX-F-G5-COPII[COPII] + TRAPPIC[COPII] + Ypt1p[COPII] + GTP[COPII] => XXX-F-G5[Golgi] + sar1-GDP[Cytoplasm] + pi[Cytoplasm] + sec13-sec31C[Cytoplasm] + sec16p[Cytoplasm] + sec12-sed24C[Cytoplasm] + Bet3p[Cytoplasm] + Bet5p[Cytoplasm] + Trs20p[Cytoplasm] + Trs23p[Cytoplasm] + Trs31p[Cytoplasm]+ Trs33p[Cytoplasm] + Bet1p[Endoplasmic Reticulum] + Bos1p[Endoplasmic Reticulum] | Irreversible | COPII (for transmembrane cargo) | ***[Ypt1p](http://www.yeastgenome.org/cgi-bin/locus.fpl?locus=ypt1),***[***Uso1p***](http://www.yeastgenome.org/cgi-bin/locus.fpl?locus=uso1)***,***[***bug1p***](http://www.yeastgenome.org/cgi-bin/locus.fpl?locus=bug1)***, \|TRAPPIC (*** [***Bet3p***](http://www.yeastgenome.org/cgi-bin/locus.fpl?locus=bet3)***,*** [***Bet5p***](http://www.yeastgenome.org/cgi-bin/locus.fpl?locus=bet5)***,***[***Trs20p***](http://www.yeastgenome.org/cgi-bin/locus.fpl?locus=trs20)***,***[***Trs23p***](http://www.yeastgenome.org/cgi-bin/locus.fpl?locus=trs23)***,***[***Trs31p***](http://www.yeastgenome.org/cgi-bin/locus.fpl?locus=trs31)***,***[***Trs33p***](http://www.yeastgenome.org/cgi-bin/locus.fpl?locus=trs33)***)*** | This proteins involve in this reaction summarize the docking and tethering step of the COPII vesicles to the Golgi membrane.Ypt1p is a rab family protein and TRAPPIC acts as NEFs of the Ypt1. Uso1p is an essential protein involved in the vesicle-mediated ER to Golgi transport along with Ypt1p and it is required for assembly of the ER-to-Golgi SNARE complex | [[125](#_ENREF_125)]. [[23](#_ENREF_23), [128-133](#_ENREF_128)] |
| 81 | XXX- ERGL6 | COPII fusion | XXX-5-G5-COPII[COPII] + TRAPPIC[COPII] + Ypt1p[COPII] + GTP[COPII] => XXX-F-G5[Golgi] + sar1-GDP[Cytoplasm] + pi[Cytoplasm] + sec13-sec31C[Cytoplasm] + sec16p[Cytoplasm] + sec12-sed24C[Cytoplasm] + Erv29p[Endoplasmic Reticulum] + 2 Bet3p[Cytoplasm] + Bet5p[Cytoplasm] + Trs20p[Cytoplasm] + Trs23p[Cytoplasm] + Trs31p[Cytoplasm]+ Trs33p[Cytoplasm] + Bet1p[Endoplasmic Reticulum] + Bos1p[Endoplasmic Reticulum] | Irreversible | COPII(for soluble cargo) | [***Ypt1p***](http://www.yeastgenome.org/cgi-bin/locus.fpl?locus=ypt1)***,***[***Uso1p***](http://www.yeastgenome.org/cgi-bin/locus.fpl?locus=uso1)***,***[***bug1p***](http://www.yeastgenome.org/cgi-bin/locus.fpl?locus=bug1)***, \|TRAPPIC (*** [***Bet3p***](http://www.yeastgenome.org/cgi-bin/locus.fpl?locus=bet3)***,*** [***Bet5p***](http://www.yeastgenome.org/cgi-bin/locus.fpl?locus=bet5)***,***[***Trs20p***](http://www.yeastgenome.org/cgi-bin/locus.fpl?locus=trs20)***,***[***Trs23p***](http://www.yeastgenome.org/cgi-bin/locus.fpl?locus=trs23)***,***[***Trs31p***](http://www.yeastgenome.org/cgi-bin/locus.fpl?locus=trs31)***,***[***Trs33p***](http://www.yeastgenome.org/cgi-bin/locus.fpl?locus=trs33)***)*** |  | [[125](#_ENREF_125)]. [[23](#_ENREF_23), [128-130](#_ENREF_128)] |
| 82 | XXX- ERGL7 | COPII fusion | XXX-F-G7-COPII[COPII] + TRAPPIC[COPII] + Ypt1p[COPII] + GTP[COPII] => XXX-F-G7[Golgi] + sar1-GDP[Cytoplasm] + pi[Cytoplasm] + sec13-sec31C[Cytoplasm] + sec16p[Cytoplasm] + Sec23p[COPII] + Sec 24p[COPII] + Emp24p[Endoplasmic Reticulum] + Bet3p[Cytoplasm] + Bet5p[Cytoplasm] + Trs20p[Cytoplasm] + Trs23p[Cytoplasm] + Trs31p[Cytoplasm]+ Trs33p[Cytoplasm] + Bet1p[Endoplasmic Reticulum] + Bos1p[Endoplasmic Reticulum] | Irreversible | COPII(for GPI-anchored cargo) | [***Ypt1p***](http://www.yeastgenome.org/cgi-bin/locus.fpl?locus=ypt1)***,***[***Uso1p***](http://www.yeastgenome.org/cgi-bin/locus.fpl?locus=uso1)***,***[***bug1p***](http://www.yeastgenome.org/cgi-bin/locus.fpl?locus=bug1)***, \|TRAPPIC (*** [***Bet3p***](http://www.yeastgenome.org/cgi-bin/locus.fpl?locus=bet3)***,*** [***Bet5p***](http://www.yeastgenome.org/cgi-bin/locus.fpl?locus=bet5)***,***[***Trs20p***](http://www.yeastgenome.org/cgi-bin/locus.fpl?locus=trs20)***,***[***Trs23p***](http://www.yeastgenome.org/cgi-bin/locus.fpl?locus=trs23)***,***[***Trs31p***](http://www.yeastgenome.org/cgi-bin/locus.fpl?locus=trs31)***,***[***Trs33p***](http://www.yeastgenome.org/cgi-bin/locus.fpl?locus=trs33)***)*** |  | [[125](#_ENREF_125)]. [[23](#_ENREF_23), [128-130](#_ENREF_128)] |
| 83 | XXX-GLER1 | COPI formation | XXX-F-G5orG7[Golgi] + Rer1p[Golgi] + Arf1p-GTP[Cytoplasm] + COPIC[Cytoplasm] + Erd2p[Golgi] + COPIV-SNARE[Cytoplasm] <=> XXX-F-G7orG5-COPI[COPI] | Irreversible | COPI | [***Arf1p***](http://www.yeastgenome.org/cgi-bin/locus.fpl?locus=ARF1),[***Rer1p***](http://www.yeastgenome.org/cgi-bin/locus.fpl?locus=rer1)***,*** [***Erd2p***](http://www.yeastgenome.org/cgi-bin/locus.fpl?locus=erd2)  ***COPIC(*** [***Cop1p***](http://www.yeastgenome.org/cgi-bin/locus.fpl?locus=cop1)***,*** [***Sec26p***](http://www.yeastgenome.org/cgi-bin/locus.fpl?locus=sec26)***,*** [***Sec27p***](http://www.yeastgenome.org/cgi-bin/locus.fpl?locus=sec27)***,*** [***Sec21p***](http://www.yeastgenome.org/cgi-bin/locus.fpl?locus=sec21)***,***[***Ret2p***](http://www.yeastgenome.org/cgi-bin/locus.fpl?locus=ret2)***,***[***Sec28p***](http://www.yeastgenome.org/cgi-bin/locus.fpl?locus=sec28)***,***[***Ret3p***](http://www.yeastgenome.org/cgi-bin/locus.fpl?locus=ret3)***)\| COPIV-SNARE*** | Arf1p (member of GTPase Ras superfamily) has a key role in regulating the formation of COPIC (coat complex).Arf1p activity regulation in controlled by guanine nucleotide exchange factors (GEF) such as Gea1p,Gea2p,Sec7p etc. The coat assembly starts and the coatamer complex and cargo selection protein (Rer1p) recruit. | [[134-137](#_ENREF_134)] |
| 84 | XXX- GLER2 | COPI uncoating and fission | XXX-F-G5orG7-COPI[COPI] + Glo3p[Cytoplasm] => XXX-F-G7orG5[Endoplasmic Reticulum] + Arf1p-GDP[Cytoplasm] + Rer1p[Golgi] + Sec27p[Cytoplasm] + Sec21p[Cytoplasm] + Erd2p[Cytoplasm]+ Ret1p[Cytoplasm] + Sec26p[Cytoplasm] + Sec27p[Cytoplasm] + Sec21p[Cytoplasm] + Ret2p[Cytoplasm] + Sec28p[Cytoplasm] + Ret3p[Cytoplasm] + GDP[Cytoplasm] + 2 pi[Cytoplasm] + COPIv-SNARE[Cytoplasm] | Irreversible | COPI | [***Rer1p***](http://www.yeastgenome.org/cgi-bin/locus.fpl?locus=rer1)***,***[***Ret2p***](http://www.yeastgenome.org/cgi-bin/locus.fpl?locus=rer2)***,***[***Cop1p***](http://www.yeastgenome.org/cgi-bin/locus.fpl?locus=cop1)***,***[***Sec27p***](http://www.yeastgenome.org/cgi-bin/locus.fpl?locus=sec27)***,***[***Sec21p***](http://www.yeastgenome.org/cgi-bin/locus.fpl?locus=sec21)***,***[***Bet1p***](http://www.yeastgenome.org/cgi-bin/locus.fpl?locus=bet1) | The uncoating and fusion are two separate step in ER-Golgi vesicle transport, but for simplicity these processes lumped in one reaction .By hydolysing of the ARF1-GTP the uncoating starts and then the uncoated vesicle bind to the golgi membrane by the t-SNARE s. | [[134](#_ENREF_134), [135](#_ENREF_135)] |
| 85 | XXX- ERADL1 | ER demanosylation II; ERAD check point | XXX-misf-G5[Endoplasmic Reticulum] + 4 Kar2p[Endoplasmic Reticulum] + 2 Pdi1pEndoplasmic Reticulum] + Mnl1p[Endoplasmic Reticulum] + 2 FADH2[Endoplasmic Reticulum] => XXX-misf-G8-cplx[Endoplasmic Reticulum] + man[Endoplasmic Reticulum] + 2 FAD[Endoplasmic Reticulum] | Irreversible | ERADL | [***Mnl1p***](http://www.yeastgenome.org/cgi-bin/locus.fpl?locus=mnl1)***,*** [***Kar2p***](http://www.yeastgenome.org/cgi-bin/locus.fpl?locus=kar2)***,***[***Pdi1p***](http://www.yeastgenome.org/cgi-bin/locus.fpl?locus=pdi1) | Mnl1p/Htm1p (α1;2-specific exomannosidase) generates the Man_7_GlcNAc_2_ oligosaccharide with a terminal α1;6-linked mannosyl residue on misfolding proteins. Processing of the N-glycan by glucosidase I; glucosidase II; and mannosidase I is perquisite for Mnl1p exomannosidase activity which result in a sequential order of specific N-glycan structures that reflect the folding status of the glycoprotein.Mnl1p works in complex with Pdi1p and Kar2p. | [[138-140](#_ENREF_138)] |
| 86 | XXX - ERADL2 | Survaliancecplx formation of misfoled protein | XXX-misf-G8-cplx[Endoplasmic Reticulum] + Yos9p[Endoplasmic Reticulum] + Hrd1-Hrd3C[Endoplasmic Reticulum] => XXX-misf-G8-surC[Endoplasmic Reticulum] + 2 Pdi1p[Endoplasmic Reticulum] + 2 Mnl1p[Endoplasmic Reticulum] ERADL] | Irreversible | ERADL | [***Yos9p***](http://www.yeastgenome.org/cgi-bin/locus.fpl?locus=yos9)***,*** [***Hrd3p***](http://www.yeastgenome.org/cgi-bin/locus.fpl?locus=hrd3)***,*** [***Hrd1p***](http://www.yeastgenome.org/cgi-bin/locus.fpl?locus=hrd1)***,*** [***Usa1p***](http://www.yeastgenome.org/cgi-bin/locus.fpl?locus=usa1)***,*** [***Der1p***](http://www.yeastgenome.org/cgi-bin/locus.fpl?locus=der1) | Yos9p (ER quality-control lectin) is an integral subunit of the HRD ligase and binds to glycans with terminal alpha-1;6 linked mannose on misfolded N-glycosylated proteins and participates in targeting proteins to ERAD. Yos9p in conjunction with Hrd3p triggers the ubiquitin-proteasome–dependent hydrolysis of these glycoproteins. The proteins such as Usa1p and Der1p provide a scaffold for the assembly of degradation machinery. | [[141](#_ENREF_141), [142](#_ENREF_142)] |
| 87 | XXX - ERADL3 | Co retrotranslocation ubiquitin addition to the misfold prtotein | XXX-misf-G8-surC[Endoplasmic Reticulum] + Ubc7p[Cytoplasm] + Cue1p[Cytoplasm] + AAAC[Cytoplasm] => XXX-misf-G8-UBcplx[Endoplasmic Reticulum] + 4 kar2p[Endoplasmic Reticulum] + 2 Pdi1p[Endoplasmic Reticulum] + Yos9p[Endoplasmic Reticulum | Irreversible | ERADL | [***Usa1p***](http://www.yeastgenome.org/cgi-bin/locus.fpl?locus=usa1)***, ,***[***Der1p***](http://www.yeastgenome.org/cgi-bin/locus.fpl?locus=der1)***,***[***Ubx2p***](http://www.yeastgenome.org/cgi-bin/locus.fpl?locus=ubx2)***,***  [***Hrd1p***](http://www.yeastgenome.org/cgi-bin/locus.fpl?locus=hrd1)***,SSH1C(***[***Ssh1p***](http://www.yeastgenome.org/cgi-bin/locus.fpl?locus=ssh1)***,*** [***Sbh2p***](http://www.yeastgenome.org/cgi-bin/locus.fpl?locus=sbh2)***,*** [***Sss1p***](http://www.yeastgenome.org/cgi-bin/locus.fpl?locus=sss1)***)*** | Hrd3p interacts with Kar2p and Yos9p to specifically target misfolded cytosolic proteins.  Sec61p, the largest and major subunit of the SEC61C( translocon) facilitates backward transport of mis-folded proteins to the cytoplasm for degradation. The other subunits of the Sec61 complex (Sss1p and Sbh1p) stabilize the complex. | [[143](#_ENREF_143), [144](#_ENREF_144)], |
| 88 |  |  | XXX -misf-(G)-UBcplx[Endoplasmic Reticulum] ?ATP[Cytoplasm] + ub => XXX -misf-(G)-UB[Cytoplasm] + Ubc7p[Cytoplasm] + Cue1p[Cytoplasm] + Ubx2p[Endoplasmic Reticulum]+ Cdc48p[Cytoplasm] + Ufd1p[Cytoplasm] + Npl4p[Cytoplasm]+ Hrd3p[Endoplasmic Reticulum] + Hrd1p[Endoplasmic Reticulum] + Usa1p[Endoplasmic Reticulum] + Der1p[Endoplasmic Reticulum] | Irreversible | ERADL | ***SSH1C(*** [***Sbh1p***](http://www.yeastgenome.org/cgi-bin/locus.fpl?locus=sbh1)***,*** [***Sss1p***](http://www.yeastgenome.org/cgi-bin/locus.fpl?locus=sss1)***,*** [***Ssh1p***](http://www.yeastgenome.org/cgi-bin/locus.fpl?locus=sbh1)***),*** [***Ubc7p***](http://www.yeastgenome.org/cgi-bin/locus.fpl?locus=ubc7)***,*** [***Cue1p***](http://www.yeastgenome.org/cgi-bin/locus.fpl?locus=cue1)***, AAAC(*** [***Ubx2p***](http://www.yeastgenome.org/cgi-bin/locus.fpl?locus=ubx2)***,*** [***Cdc48p***](http://www.yeastgenome.org/cgi-bin/locus.fpl?locus=cdc48)***,*** [***Ufd1p***](http://www.yeastgenome.org/cgi-bin/locus.fpl?locus=ufd1)***,*** [***Npl4p***](http://www.yeastgenome.org/cgi-bin/locus.fpl?locus=npl4)***), Hrd1-Hrd3C(*** [***Hrd3p***](http://www.yeastgenome.org/cgi-bin/locus.fpl?locus=hrd3)***,*** [***Hrd1p***](hrd1)***,*** [***Usa1p***](usa1)***,*** [***Der1p***](der1)***)*** | Misfolded luminal and membrane proteins are ubiquitinated by Hrd1p  (ER membrane) . Each ubiquitin ligase complex interacts with the Cdc48p-Npl4p-Ufd1p AAA ATPase complex via Ubx2p in order to extract ubiquitinated substrates from the ER. Hrd1p-Hrd3pC interacts with Kar2p (by Hrd3p) and Yos9p and specifically target misfolded cytosolic proteins. Ubc7p or Ubc1p can act as the ubiquitin-conjugating enzyme (E2) for Hrd1p. Ubc7p interacts with Hrd1p(RING-H2 domain). |  |
| 89 | XXX - ERADL4 |  | XXX-misf-G8-UBcplx[Endoplasmic Reticulum] + 50 ATP[Cytoplasm] + 50 ub[Cytoplasm] + Uba1p[Cytoplasm] => XXX-misf-G8-UB[Cytoplasm] + Ubc7p[Cytoplasm] + Cue1p[Cytoplasm] + Ubx2p[Endoplasmic Reticulum]+ Cdc48p[Cytoplasm] + Ufd1p[Cytoplasm] + Npl4p[Cytoplasm]+ Hrd3p[Endoplasmic Reticulum] + Hrd1p[Endoplasmic Reticulum] + Usa1p[Endoplasmic Reticulum] + Der1p[Endoplasmic Reticulum] | Irreversible | ERADL | [***Dsk2p***](http://www.yeastgenome.org/cgi-bin/locus.fpl?locus=dsk2)***,***[***Rad23p***](http://www.yeastgenome.org/cgi-bin/locus.fpl?locus=rad23)***,***[***Png1p***](http://www.yeastgenome.org/cgi-bin/locus.fpl?locus=png1)***,*** [***Uba1p***](http://www.yeastgenome.org/cgi-bin/locus.fpl?locus=uba1) |  |  |
| 90 | XXX-ERADL5 |  | XXX-misf-G8-UB[Cytoplasm] + Png1[Cytoplasm] + 50 ATP[Cytoplasm] + 26sproteosome[Cytoplasm] + 50 H2O[Cytoplasm] => G8[Cytoplasm] + 50 Uniquitin[Cytoplasm] + 50 ADP[Cytoplasm] + 50 pi[Cytoplasm] + aminoacid[Cytoplasm] | Irreversible | ERADL |  |  |  |
| 91 | XXX-ERADM1 |  | XXX-misf-G8-cplx[Endoplasmic Reticulum] + Yos9p[Endoplasmic Reticulum] + Hrd1p-Hrd3pC[Endoplasmic Reticulum] => XXX-misf-G8-HRDcplx[Endoplasmic Reticulum] | Irreversible | ERADM | [***Yos9p***](http://www.yeastgenome.org/cgi-bin/locus.fpl?locus=yos9)***, HRD[***[***Hrd1p***](http://www.yeastgenome.org/cgi-bin/locus.fpl?locus=hrd1)***,*** [***Hrd3p***](http://www.yeastgenome.org/cgi-bin/locus.fpl?locus=hrd3)***]*** | Substrates with misfolded intramembrane domains define a use ERAD-M pathway whicht differs from ERAD-L and ERAD-C its independence of Usa1p and Der1p.  Substrates of the ERAD-M pathway may be targeted to the ubiquitin ligase Hrd1p by Yos9p, as is the case for the glycosylated substrate Pdr5*, or by other factors that are too loosely associated to be detected by pull-down experiments. | [[10](#_ENREF_10), [145](#_ENREF_145)] |
| 92 | XXX-ERADM2 |  | XXX-misf-G8-HRDcplx[Endoplasmic Reticulum] + Ubc7p[Cytoplasm] + Cue1p[Cytoplasm] + AAAC[Cytoplasm] => XXX-misf-G8-UBcplx[Endoplasmic Reticulum] + Hrd3p[Endoplasmic Reticulum] + Hrd1p[Endoplasmic Reticulum] + Usa1p[Endoplasmic Reticulum] + Der1p[Endoplasmic Reticulum] + Yos9p[Endoplasmic Reticulum] | Irreversible | ERADM | ***AAAC(***[***Ubx2p***](http://www.yeastgenome.org/cgi-bin/locus.fpl?locus=ubx2)***,*** [***Cdc48p***](http://www.yeastgenome.org/cgi-bin/locus.fpl?locus=cdc48)***,***[***Ufd1p***](http://www.yeastgenome.org/cgi-bin/locus.fpl?locus=ufd1)***,*** [***Npl4p***](http://www.yeastgenome.org/cgi-bin/locus.fpl?locus=npl4)***)*** | The rest of the ERAD-M pathway would be similar to that of ERAD-L substrates, employing the  Cdc48p ATPase complex and the adaptor Ubx2p. However,ERAD-M might either use a smaller channel consisting of Hrd1p alone or directly be extracted from the membrane, as what it has been shown to happen for bacterial FtsH protein . | [[10](#_ENREF_10), [146](#_ENREF_146)] |
| 93 | XXX-ERADM3 |  | XXX-misf-G8-UBcplx[Endoplasmic Reticulum] + 50 ATP[Cytoplasm] + 50 ub[Cytoplasm] => XXX-misf-G8-UB[Cytoplasm] + Ubc7p[Cytoplasm] + Cue1p[Cytoplasm] + Ubx2p[Endoplasmic Reticulum]+ Cdc48p[Cytoplasm] + Ufd1p[Cytoplasm] + Npl4p[Cytoplasm] Hrd3p[Endoplasmic Reticulum] + Hrd1p[Endoplasmic Reticulum] + Usa1p[Endoplasmic Reticulum] + Der1p[Endoplasmic Reticulum] | Irreversible | ERADM |  |  |  |
| 94 | XXX-ERADM4 |  | XXX-misf-G8-UB[Cytoplasm] + Dsk2pp[Cytoplasm] + Rad23pp[Cytoplasm] + Png1p[Cytoplasm] + 50 ATP[Cytoplasm] + 26sproteosome[Cytoplasm] + 50 H2O[Cytoplasm] => G8[Cytoplasm] + 50 Uniquitin[Cytoplasm] + 50 ADP[Cytoplasm] + 50 pi[Cytoplasm] + aminoacid[Cytoplasm] | Irreversible | ERADM |  |  |  |
| 95 | XXX-ERADC1 |  | XXX-misf-G6[Cytoplasm] + Doa10p[Endoplasmic Reticulum] + SSH1C[Endoplasmic Reticulum] + AAAC[Cytoplasm] => XXX-misf-G6-UBcplx[Cytoplasm] | Irreversible | ERADC | [***Sbh1p***](http://www.yeastgenome.org/cgi-bin/locus.fpl?locus=sbh1)***,*** [***Sss1p***](http://www.yeastgenome.org/cgi-bin/locus.fpl?locus=sss1)***, Ssh1p\|AAAC(*** [***Ubx2p***](http://www.yeastgenome.org/cgi-bin/locus.fpl?locus=ubx2)***,*** [***Cdc48p***](http://www.yeastgenome.org/cgi-bin/locus.fpl?locus=cdc48)***,*** [***Ufd1p***](http://www.yeastgenome.org/cgi-bin/locus.fpl?locus=ufd1)***,*** [***Npl4p***](http://www.yeastgenome.org/cgi-bin/locus.fpl?locus=npl4)***)*** | ERAD-C pathway is potential to degrade the membrane proteins with cytoplasmic misfolded domain. While the core proteins are the same, it is shown that the deletion of USA1 does not affect the degradation of ERAD-C substrate. Also, Usa1p is functionally required for the ERAD-L but not the ERAD-C pathway. Similar to the other ERAD pathways the for the degradation part ERAD-C use also use the common protein machinery AAAC and proteasome. | [[145](#_ENREF_145), [147](#_ENREF_147)] |
| 96 | XXX-ERADC2 |  | XXX-misf-G6-UBcplx[Endoplasmic Reticulum] + 50 ATP[Cytoplasm] + 50 ub[Cytoplasm] => XXX-misf-G6-UB + 50 ADP[Cytoplasm] + Doa10p[Endoplasmic Reticulum] Sbh1p[Endoplasmic Reticulum] + Sss1p[Endoplasmic Reticulum] + Ssh1p[Endoplasmic Reticulum] + Ubx2p[Endoplasmic Reticulum]+ Cdc48p[Cytoplasm] + Ufd1p[Cytoplasm] + Npl4p[Cytoplasm] + 50 pi[Cytoplasm] | Irreversible | ERADC |  |  |  |
| 97 | XXX-ERADC3 |  | XXX-misf-G6-UB[Cytoplasm] + Dsk2p[Cytoplasm] + Rad23p[Cytoplasm] + Png1[Cytoplasm] + 50 ATP[Cytoplasm] + 26s proteosome[Cytoplasm] + 50 H2O[Cytoplasm] => G6[Cytoplasm] + 50 Uniquitin[Cytoplasm] + 50 ADP[Cytoplasm] + 50 pi[Cytoplasm] + aminoacid[Cytoplasm] | Irreversible | ERADC |  |  |  |
| 98 | XXX- GLNMAN1 | Golgi N-linked glycosylation I | XXX-G5orG7[Golgi] + GDP-man[Golgi] => XXX-G9[Golgi] + GDP[Golgi] | Irreversible | Golgi processing | [***Och1p***](http://www.yeastgenome.org/cgi-bin/locus.fpl?locus=och1) | Och1p( *cis*-Golgi membrane-bound alpha-1;6-mannosyltransferase ) adds a mannose moiety to core N-linked oligosaccharides upon their arrival in the Golgi apparatus. This is the last modification before the N-linked glycosylation pathway forks to produce either large mannan outer chains or small core-type oligosaccharides. | [[148](#_ENREF_148), [149](#_ENREF_149)]. |
| 99 | XXX- GLNMAN2 | Golgi N-linked glycosylation II | XXX-G9[Golgi] + 5 GDP-man[Golgi] => XXX-G10[Golgi] + 5 GDP[Golgi] | Irreversible | Golgi processing | ***M POL I(***[***Mnn9p***](http://www.yeastgenome.org/cgi-bin/locus.fpl?locus=mnn9)***,*** [***Van1p***](http://www.yeastgenome.org/cgi-bin/locus.fpl?locus=van1)***)*** | M-Pol I is a heterodimeric complex consisting of one copy of Van1p and one copy of Mnn9p.Mnn9p Acts as Both an α-1;2- and an α-1;6-Mannosyltransferase. Both proteins contribute to mannose olymerization in The Golgi.Mnn9p also take part in M Pol II complex. | [[150](#_ENREF_150)] |
| 100 | XXX- GLNMAN3 | Golgi N-linked glycosylation III | XXX-G10[Golgi] + 40 GDP-man[Golgi] => XXX-G11[Golgi] + 40 GDP[Golgi] | Irreversible | Golgi processing | ***M Pol II complex (***[***Anp1p***](http://www.yeastgenome.org/cgi-bin/locus.fpl?locus=anp1)***,*** [***Mnn9p***](http://www.yeastgenome.org/cgi-bin/locus.fpl?locus=mnn9)***,*** [***Mnn10p***](mnn10)***,*** [***Mnn11p***](http://www.yeastgenome.org/cgi-bin/locus.fpl?locus=mnn11)***,*** [***Hoc1p***](http://www.yeastgenome.org/cgi-bin/locus.fpl?locus=hoc1)***)*** | M-Pol II, Golgi mannosyltransferase complex, involves five subunits (at least two of which are present in multiple copies). It contains Mnn9p Anp1p, Mnn10p, Mnn11p, and Hoc1p and elongate the polysaccharide mannan chain | [[150](#_ENREF_150)] |
| 101 | XXX GLOMAN1 | Golgi N-linked pro-peptide hydrolase | XXX-G12[Golgi] + GDP-man[Golgi] => XXX-G13[Golgi] + GDP[Golgi] | Irreversible | Golgi processing | [***Kre2p***](http://www.yeastgenome.org/cgi-bin/locus.fpl?locus=kre2)***,***[***Ktr1p***](http://www.yeastgenome.org/cgi-bin/locus.fpl?locus=ktr1)***,***[***Ktr3p***](http://www.yeastgenome.org/cgi-bin/locus.fpl?locus=ktr3) | Ktr1p and Ktr3p along with the Kre2p/Mnt1p α1,2-mannosyltransferase participate in the addition of the second mannose residue onto O-linked chains. Kre2p has been known to be the main enzyme responsible for the addition of the third mannose on O-glycans. Ktr1p and Ktr3p are also able to add this particular mannose, although to a lesser extent than Kre2p. | [[151-153](#_ENREF_151)] |
| 102 | XXX- GLOMAN3 | First golgi N-linked O –linked manosylation | XXX-G13[Golgi] + GDP-man[Golgi] => XXX-G14[Golgi] + GDP[Golgi] | Irreversible | Golgi processing | [***Kre2p***](http://www.yeastgenome.org/cgi-bin/locus.fpl?locus=kre2) | Kre2, alpha-1,2-mannosyltransferase which together with Ktr1p and Ktr3p is responsible for the second and the third α1,2-linked mannose residues on O-linked carbohydrate chains and that also participate in N-linked outer chain elaboration. , | [[151](#_ENREF_151)] |
| 103 | XXX- GLOMAN3 | Second golgi N-linked O –linked manosylation | XXX-G14[Golgi] + GDP-man[Golgi] => XXX-15[Golgi] + GDP[Golgi] | Irreversible | Golgi processing | [***Mnn1p***](http://www.yeastgenome.org/cgi-bin/locus.fpl?locus=mnn1) | The Mnn1p α1,3- annosyltransferase attaches the fourth mannose residue in the linear chain of up to five mannose residues | [[151](#_ENREF_151)] |
| 104 | XXX- GP | golgi N-linked O –linked pro-peptide hydrolization | XXX-G15[Golgi] => XXX-G16[Golgi] + aminoacid[Golgi] | Irreversible | Golgi processing | [***Kex1p***](http://www.yeastgenome.org/cgi-bin/locus.fpl?locus=kex1)***,***[***Kex2p***](http://www.yeastgenome.org/cgi-bin/locus.fpl?locus=kex2)***,***[***Ste13p***](http://www.yeastgenome.org/cgi-bin/locus.fpl?locus=ste13) | Maturation of the proteins in The Golgi needs cleavage of the polypeptide chain. There are three known protease (Kex1p, Kex2p and Ste13p) in yeast Golgi which are specific to sequence motifs and provide the proteins to be catalytically active or be able to bind to specific receptor. | [[154](#_ENREF_154)] |
| 105 | XXX- ALP | Direct vacuol transit pathway | XXX-M1[Golgi] + 4 GTP[Cytoplasm] + clathrinC[Cytoplasm] + Arf1p[Cytoplasm] + AP3C[Cytoplasm] + Vps1p[Cytoplasm] + Swa2p[Cytoplasm] => XXX-M1[vacuole] + 4 GDP[Cytoplasm] + + Arf1p[Cytoplasm] + Apl6p[Cytoplasm] + Aps3p[Cytoplasm] + Apm3p[Cytoplasm] + Apl5p[Cytoplasm] + Vam3p[Vacuole] + Vps1p[Cytoplasm] + Swa2p[Cytoplasm] | Irreversible | ALP pathway(AP-3 complex) | ***AP3C(*** [***Apl6p***](http://www.yeastgenome.org/cgi-bin/locus.fpl?locus=apl6)***,*** [***Aps3p***](http://www.yeastgenome.org/cgi-bin/locus.fpl?locus=aps3)***,*** [***Apm3p***](http://www.yeastgenome.org/cgi-bin/locus.fpl?locus=apm3)***,*** [***Apl5p***](http://www.yeastgenome.org/cgi-bin/locus.fpl?locus=apl5)***,***[***Vam3p***](http://www.yeastgenome.org/cgi-bin/locus.fpl?locus=vam3) ***)\|t-SNARE\|*** [***Arf1p***](http://www.yeastgenome.org/cgi-bin/locus.fpl?locus=arf1)***\| clathrinC(***[***Chc1p***](http://www.yeastgenome.org/cgi-bin/locus.fpl?locus=chc1)***,***[***Clc1p***](http://www.yeastgenome.org/cgi-bin/locus.fpl?locus=clc1)***)***  ***Vps1p,Swa2p*** | After Golgi processing the mature protein can transport to many destination depended on final localization. ALP pathway is one of the known trafficking routes from Golgi to vacuole. Many key proteins involved as detecting ( ***Vps1p,Swa2p)***, tethering(,ClathrinC,Arf1p) and docking(t-SNAREC) of the vesicles ALP pathway are characterized. Apm3:Mu3-like subunit of the clathrin associated protein complex (AP-3); Apl6: Beta3-like subunit of the AP-3 complex;Aps3p: Small subunit of the clathrin-associated adaptor complex AP-3; Apl5p: Delta adaptin-like subunit of the clathrin associated protein complex (AP-3) are among these proteins which are involved in this transport route. | [[21](#_ENREF_21), [155](#_ENREF_155)] |
| 106 | XXX- CPY1 | CPYI | XXX-M1[Golgi] + 4 GTP[Cytoplasm] + Pep12p[endosome] + Vps45p[endosome] + Vps5p[Golgi] + clathrinC[Cytoplasm] + Arf1p[Cytoplasm] + Gga1p[Cytoplasm] + Gga2p[Cytoplasm] + AP1C[Cytoplasm] + Swa2p[Cytoplasm] => XXX-M1[endosome] + 4 GDP[Cytoplasm] + Pi[Cytoplasm] + Apl6p[Cytoplasm] + Aps3p[Cytoplasm] + Apm3p[Cytoplasm] + Apl5p[Cytoplasm] + Vam3p[Vacuole] + 2 Chc1p[Cytoplasm] + Clc1p[Cytoplasm] | Irreversible | CPY pathway | ***CPYIC,*** [***Gga1p***](http://www.yeastgenome.org/cgi-bin/locus.fpl?locus=gga1)***,*** [***Gga2p***](http://www.yeastgenome.org/cgi-bin/locus.fpl?locus=gga2)***,*** [***Arf1p***](http://www.yeastgenome.org/cgi-bin/locus.fpl?locus=arf1);***AP1C(*** [***Apl4p***](http://www.yeastgenome.org/cgi-bin/locus.fpl?locus=apl4)***,*** [***Apl2p***](http://www.yeastgenome.org/cgi-bin/locus.fpl?locus=apl2)  [***Apm1p***](http://www.yeastgenome.org/cgi-bin/locus.fpl?locus=apm1)***,***  [***Aps1p***](http://www.yeastgenome.org/cgi-bin/locus.fpl?locus=aps1)***;clathrinC(***[***Chc1p***](http://www.yeastgenome.org/cgi-bin/locus.fpl?locus=chc1)***,***[***Clc1p***](http://www.yeastgenome.org/cgi-bin/locus.fpl?locus=clc1)***);***  [***Pep12p***](http://www.yeastgenome.org/cgi-bin/locus.fpl?locus=pep12)***,*** [***Vps45p***](http://www.yeastgenome.org/cgi-bin/locus.fpl?locus=vps45) | CPY pathway is the defult route to the vacuole from Golgi apparatus.A two step process using AP complexes. The pathway is named because it has mainly been  studied in the for trafficking of carboxypeptidase Y (CPY) to the vacuole. AP-1C complex vesicles can transfer proteins from the trans-Golgi to the early or late  endosome. After this, the AP-3C complex vesicle moves proteins from the Golgi/endosome to vacuole. | [[20](#_ENREF_20), [37](#_ENREF_37), [156](#_ENREF_156)] |
| 107 | XXX- CPY2 | CPYII | XXX-M1[endosome] + ATP[Cytoplasm] + CPYIIC[Cytoplasm] + AP3C[Cytoplasm] => XXX-M1[vacuole] + ADP[Cytoplasm] + pi[Cytoplasm] + Vps5p[Golgi] + 2 Chc1p[Cytoplasm] + Clc1p[Cytoplasm] + Gga1p[Cytoplasm] + Gga2p[Cytoplasm] + Apl4p[Cytoplasm] + Apl2p[Cytoplasm] + Apm1p[Cytoplasm] + Aps1p[Cytoplasm + Swa2p[Cytoplasm] + Arf1p[Cytoplasm] + Vps4p[endosome] + Vps27p[endosome] + Pep12p[endosome] + Vps45p[endosome] | Irreversible | CPY pathway | ***CPYIIC \|AP3C(*** [***Apl6p***](http://www.yeastgenome.org/cgi-bin/locus.fpl?locus=apl6)***,*** [***Aps3p***](http://www.yeastgenome.org/cgi-bin/locus.fpl?locus=aps3)***,*** [***Apm3p***](apm3)***,***[***Apl5p***](http://www.yeastgenome.org/cgi-bin/locus.fpl?locus=apl5)***,***[***Vam3p***](http://www.yeastgenome.org/cgi-bin/locus.fpl?locus=vam3) ***)*** |  | [[157](#_ENREF_157)] |
| 108 | XXX-LDSV | LDSV secretion | XXX-M1[Golgi] + GTP[Cytoplasm] + clathrinC[Cytoplasm] + Arf1p[Cytoplasm] + EXOC[Cytoplasm] => XXX-M1[cell membrane] + GDP[Golgi] + 2 Chc1p[Cytoplasm] + Clc1p[Cytoplasm] + Arf1p[Cytoplasm] + Sec3p[Cytoplasm] + Sec5p[Cytoplasm] + Sec6p[Cytoplasm] + Sec8p[Cytoplasm] + Sec10p[Cytoplasm] + Sec15p[Cytoplasm] + Exo70p[Cytoplasm] + Exo84p[Cytoplasm] + sec4p[Cytoplasm] | Irreversible | LDSV(low density secretory vesicle) | [***Arf1p***](http://www.yeastgenome.org/cgi-bin/locus.fpl?locus=arf1); ***LDSV([Sec3p](http://www.yeastgenome.org/cgi-bin/locus.fpl?locus=sec3),*** [***Sec5p***](http://www.yeastgenome.org/cgi-bin/locus.fpl?locus=sec5)***,*** [***Sec6p***](http://www.yeastgenome.org/cgi-bin/locus.fpl?locus=sec6)***,*** [***Sec8p***](http://www.yeastgenome.org/cgi-bin/locus.fpl?locus=sec8)***,*** [***Sec10p***](http://www.yeastgenome.org/cgi-bin/locus.fpl?locus=sec10)***,*** [***Sec15p***](http://www.yeastgenome.org/cgi-bin/locus.fpl?locus=sec15)***,*** [***Exo70p***](http://www.yeastgenome.org/cgi-bin/locus.fpl?locus=sec70)***,*** [***Exo84p***](http://www.yeastgenome.org/cgi-bin/locus.fpl?locus=exo84)***,***[***Sec4p***](http://www.yeastgenome.org/cgi-bin/locus.fpl?locus=sec4)***)\| ;clathrinC(*** [***Chc1p***](http://www.yeastgenome.org/cgi-bin/locus.fpl?locus=chc1)***,*** [***Clc1p***](http://www.yeastgenome.org/cgi-bin/locus.fpl?locus=clc1)***)*** | There are two types of vesicle( pathway) for proteins that will follow the exocytotic pathway from the trans-Golgi called light density secretory vesicles (LDSV) and heavy density secretory vesicles (HDSV)(upon density-based separation experiments). LDSV are known to carry  constitutively expressed cell membrane proteins, such as Bgl2p, Pma1p, and Gas1p and believed to emerge from the trans-Golgi and transit directly to the cell  membrane.  : | [[42-45](#_ENREF_42)] |
| 109 | XXX- HDSV1 | HDSV secretion | XXX-M1[Golgi] + GTP[Cytoplasm] + clathrin[Cytoplasm] + Arf1p[Cytoplasm] + Pep12p[endosome] + AP1C[Cytoplasm] + Swa2p[Cytoplasm] => XXX-M1[endosome] + GDP[Cytoplasm] + Pi[Cytoplasm] + 2 Chc1p[Cytoplasm] + Clc1p[Cytoplasm] | Irreversible | HDSV(high density secretory vesicle) | [***Arf1p***](http://www.yeastgenome.org/cgi-bin/locus.fpl?locus=arf1)***,*** [***Pep12p***](http://www.yeastgenome.org/cgi-bin/locus.fpl?locus=pep12)***,*** [***Swa2p***](http://www.yeastgenome.org/cgi-bin/locus.fpl?locus=swa2)***\| clathrinC( [Chc1p](http://www.yeastgenome.org/cgi-bin/locus.fpl?locus=chc1),*** [***Clc1p***](http://www.yeastgenome.org/cgi-bin/locus.fpl?locus=clc1)***)\| APC1( [Apl4p](http://www.yeastgenome.org/cgi-bin/locus.fpl?locus=apl4),*** [***Apl2p***](http://www.yeastgenome.org/cgi-bin/locus.fpl?locus=apl2)  [***Apm1p***](http://www.yeastgenome.org/cgi-bin/locus.fpl?locus=apm1)***,***  [***Aps1p***](http://www.yeastgenome.org/cgi-bin/locus.fpl?locus=aps1)***)\|*** |  | [[45](#_ENREF_45), [158](#_ENREF_158)] |
| 110 | XXX- HDSV2 | HDSV secretion | XXX-M1[endosome] + GTP[Cytoplasm] + clathrin[Cytoplasm] => XXX-M1[extracellular] + GDP[Cytoplasm] + Arf1p[Cytoplasm] + Pep12p[endosome] + Apl4p[Cytoplasm] + Apl2p[Cytoplasm] + Apm1p[Cytoplasm] + Aps1p[Cytoplasm] + 2 Chc1p[Cytoplasm] + Clc1p[Cytoplasm] | Irreversible | HDSV(high density secretory vesicle) | [***Vps1p***](http://www.yeastgenome.org/cgi-bin/locus.fpl?locus=vps1) ***\|clathrinC(*** [***Chc1p***](http://www.yeastgenome.org/cgi-bin/locus.fpl?locus=chc1)***,*** [***Clc1p***](http://www.yeastgenome.org/cgi-bin/locus.fpl?locus=clc1)***)*** | HDSV vesicles package soluble, secreted proteins, such as invertase  (Suc2p) and acid phosphates (Pho11p, Pho12p, Pho5p) that are transcriptionally regulated and induced under certain conditions. HDSV move from the endosome to the cell membrane, and are thus subject to many of the mutations that block movement to and through the early/late endosome . These mutants, that block the HDSV pathway, were shown to use the LDSV pathway for secretion of proteins normally bound for HDSV pathway. | [[45](#_ENREF_45)] |
|  |  |  |  |  |  |  |  |  |

**Refrences**

1. Ogg, S., M. Poritz, and P. Walter, *Signal recognition particle receptor is important for cell growth and protein secretion in Saccharomyces cerevisiae.* Molecular biology of the cell, 1992. **3**(8): p. 895.

2. Bohni, P.C., R.J. Deshaies, and R.W. Schekman, *SEC11 is required for signal peptide processing and yeast cell growth.* J. Cell Biol., 1988. **106**(4): p. 1035-1042.

3. Babst, M., et al., *Endosome-associated complex, ESCRT-II, recruits transport machinery for protein sorting at the multivesicular body.* Developmental cell, 2002. **3**(2): p. 283-289.

4. Robb, A. and J.D. Brown, *Protein Transport:: Two Translocons Are Better Than One.* Molecular cell, 2001. **8**(3): p. 484-486.

5. Finke, K., et al., *A second trimeric complex containing homologs of the Sec61p complex functions in protein transport across the ER membrane of S. cerevisiae.* The EMBO Journal, 1996. **15**(7): p. 1482.

6. Johnson, A.E. and M.A. van Waes, *The translocon: a dynamic gateway at the ER membrane.* Annu. Rev. Cell. Dev. Biol., 1999. **15**: p. 799-842.

7. Young, B.P., et al., *Sec63p and Kar2p are required for the translocation of SRP-dependent precursors into the yeast endoplasmic reticulum in vivo.* The EMBO Journal, 2001. **20**(1): p. 262-271.

8. Rothblatt, J.A., et al., *Multiple genes are required for proper insertion of secretory proteins into the endoplasmic reticulum in yeast.* The Journal of Cell Biology, 1989. **109**(6): p. 2641.

9. Herscovics, A., *Processing glycosidases of Saccharomyces cerevisiae.* Biochim. Biophys. Acta, 1999. **1426**(2): p. 275-285.

10. Carvalho, P., V. Goder, and T. Rapoport, *Distinct ubiquitin-ligase complexes define convergent pathways for the degradation of ER proteins.* Cell, 2006. **126**(2): p. 361-373.

11. Ye, Y., H.H. Meyer, and T.A. Rapoport, *The AAA ATPase Cdc48/p97 and its partners transport proteins from the ER into the cytosol.* Nature, 2001. **414**(6864): p. 652-656.

12. Miller, E., et al., *Cargo selection into COPII vesicles is driven by the Sec24p subunit.* The EMBO Journal, 2002. **21**(22): p. 6105-6113.

13. Barlowe, C., et al., *COPII: a membrane coat formed by Sec proteins that drive vesicle budding from the endoplasmic reticulum.* Cell, 1994. **77**(6): p. 895-907.

14. Yoshihisa, T., C. Barlowe, and R. Schekman, *Requirement for a GTPase-activating protein in vesicle budding from the endoplasmic reticulum.* Science, 1993. **259**(5100): p. 1466.

15. Nakano, A. and M. Muramatsu, *A novel GTP-binding protein, Sar1p, is involved in transport from the endoplasmic reticulum to the Golgi apparatus.* The Journal of Cell Biology, 1989. **109**(6): p. 2677-2691.

16. Nakano, A., D. Brada, and R. Schekman, *A membrane glycoprotein, Sec12p, required for protein transport from the endoplasmic reticulum to the Golgi apparatus in yeast.* The Journal of Cell Biology, 1988. **107**(3): p. 851.

17. Sacher, M., et al., *Identification and characterization of five new subunits of TRAPP.* European journal of cell biology, 2000. **79**(2): p. 71-80.

18. Sacher, M., et al., *TRAPP, a highly conserved novel complex on the cis-Golgi that mediates vesicle docking and fusion.* The EMBO Journal, 1998. **17**(9): p. 2494-2503.

19. Jiang, Y., et al., *A high copy suppressor screen reveals genetic interactions between BET3 and a new gene: evidence for a novel complex in ER-to-Golgi transport.* Genetics, 1998. **149**(2): p. 833-841.

20. Kirchhausen, T., *Three ways to make a vesicle.* Nature Reviews Molecular Cell Biology, 2000. **1**(3): p. 187-198.

21. Cowles, C.R., et al., *The AP-3 adaptor complex is essential for cargo-selective transport to the yeast vacuole.* Cell, 1997. **91**(1): p. 109-118.

22. Stepp, J.D., K. Huang, and S.K. Lemmon, *The yeast adaptor protein complex, AP-3, is essential for the efficient delivery of alkaline phosphatase by the alternate pathway to the vacuole.* The Journal of Cell Biology, 1997. **139**(7): p. 1761-1774.

23. Guo, W., et al., *Protein complexes in transport vesicle targeting.* Trends in cell biology, 2000. **10**(6): p. 251-255.

24. Lemmon, S.K. and E.W. Jones, *Clathrin requirement for normal growth of yeast.* Science, 1987. **238**(4826): p. 504.

25. Salama, N.R., J. Chuang, and R. Schekman, *Sec31 encodes an essential component of the COPII coat required for transport vesicle budding from the endoplasmic reticulum.* Molecular biology of the cell, 1997. **8**(2): p. 205.

26. Hughes, H. and D.J. Stephens, *Assembly, organization, and function of the COPII coat.* Histochemistry and Cell Biology, 2008. **129**(2): p. 129-151.

27. Fath, S., et al., *Structure and organization of coat proteins in the COPII cage.* Cell, 2007. **129**(7): p. 1325-1336.

28. Peyroche, A., S. Paris, and C.L. Jackson, *Nucleotide exchange on ARF mediated by yeast Geal protein.* 1996.

29. Peyroche, A., et al., *The ARF exchange factors Gea1p and Gea2p regulate Golgi structure and function in yeast.* Journal of cell science, 2001. **114**(12): p. 2241.

30. Cosson, P. and F. Letourneur, *Coatomer (COPI)-coated vesicles: role in intracellular transport and protein sorting.* Current opinion in cell biology, 1997. **9**(4): p. 484-487.

31. Duden, R., et al., *&epsi;-COP is a structural component of coatomer that functions to stabilize α-COP.* The EMBO Journal, 1998. **17**(4): p. 985-995.

32. Gerich, B., et al., *Non-clathrin-coat protein alpha is a conserved subunit of coatomer and in Saccharomyces cerevisiae is essential for growth.* Proceedings of the National Academy of Sciences, 1995. **92**(8): p. 3229.

33. Cosson, P., et al., *Delta-and zeta-COP, two coatomer subunits homologous to clathrin-associated proteins, are involved in ER retrieval.* The EMBO Journal, 1996. **15**(8): p. 1792.

34. Duden, R., et al., *Yeast beta-and beta'-coat proteins (COP). Two coatomer subunits essential for endoplasmic reticulum-to-Golgi protein traffic.* Journal of Biological Chemistry, 1994. **269**(39): p. 24486.

35. Letourneur, F., et al., *Coatomer is essential for retrieval of dilysine-tagged proteins to the endoplasmic reticulum.* Cell, 1994. **79**(7): p. 1199-1207.

36. Becherer, K., et al., *Novel syntaxin homologue, Pep12p, required for the sorting of lumenal hydrolases to the lysosome-like vacuole in yeast.* Molecular biology of the cell, 1996. **7**(4): p. 579.

37. Gerrard, S.R., B.P. Levi, and T.H. Stevens, *Pep12p is a multifunctional yeast syntaxin that controls entry of biosynthetic, endocytic and retrograde traffic into the prevacuolar compartment.* Traffic, 2000. **1**(3): p. 259-269.

38. Piper, R., E. Whitters, and T. Stevens, *Yeast Vps45p is a Sec1p-like protein required for the consumption of vacuole-targeted, post-Golgi transport vesicles.* European journal of cell biology, 1994. **65**(2): p. 305.

39. Bryant, N.J. and D.E. James, *Vps45p stabilizes the syntaxin homologue Tlg2p and positively regulates SNARE complex formation.* The EMBO Journal, 2001. **20**(13): p. 3380-3388.

40. Babst, M., et al., *The Vps4p AAA ATPase regulates membrane association of a Vps protein complex required for normal endosome function.* The EMBO Journal, 1998. **17**(11): p. 2982-2993.

41. Lottridge, J.M., et al., *Vta1p and Vps46p regulate the membrane association and ATPase activity of Vps4p at the yeast multivesicular body.* PNAS, 2006. **103**(16): p. 6202-6207.

42. Finger, F.P. and P. Novick, *Spatial regulation of exocytosis: lessons from yeast.* The Journal of Cell Biology, 1998. **142**(3): p. 609-612.

43. TerBush, D.R., et al., *The Exocyst is a multiprotein complex required for exocytosis in Saccharomyces cerevisiae.* The EMBO Journal, 1996. **15**(23): p. 6483.

44. Guo, W., et al., *The exocyst is an effector for Sec4p, targeting secretory vesicles to sites of exocytosis.* The EMBO Journal, 1999. **18**(4): p. 1071-1080.

45. Harsay, E. and A. Bretscher, *Parallel secretory pathways to the cell surface in yeast.* J. Cell Biol., 1995. **131**(2): p. 297-310.

46. Ng, D.T., J.D. Brown, and P. Walter, *Signal sequences specify the targeting route to the endoplasmic reticulum membrane.* J. Cell Biol., 1996. **134**(2): p. 269-278.

47. Gilmore, R., P. Walter, and G. Blobel, *Protein translocation across the endoplasmic reticulum. II. Isolation and characterization of the signal recognition particle receptor.* The Journal of Cell Biology, 1982. **95**(2): p. 470.

48. Martoglio, B. and B. Dobberstein, *Signal sequences: more than just greasy peptides.* Trends Cell Biol., 1998. **8**(10): p. 410-415.

49. Osborne, A.R., T.A. Rapoport, and B. van den Berg, *Protein translocation by the Sec61/SecY channel.* Annu. Rev. Cell Dev. Biol., 2005. **21**: p. 529-550.

50. Van den Berg, B., et al., *X-ray structure of a protein-conducting channel.* Nature, 2004. **427**(6969): p. 36-44.

51. Rapiejko, P.J. and R. Gilmore, *Empty site forms of the SRP54 and SR alpha GTPases mediate targeting of ribosome-nascent chain complexes to the endoplasmic reticulum.* Cell, 1997. **89**(5): p. 703-713.

52. Egea, P.F., et al., *Substrate twinning activates the signal recognition particle and its receptor.* Nature, 2004. **427**(6971): p. 215-221.

53. Rapiejko, P.J. and R. Gilmore, *Empty site forms of the SRP54 and SRα GTPases mediate targeting of ribosome–nascent chain complexes to the endoplasmic reticulum.* Cell, 1997. **89**(5): p. 703-713.

54. Walter, P. and V.R. Lingappa, *Mechanism of protein translocation across the endoplasmic reticulum membrane.* Annual review of cell biology, 1986. **2**(1): p. 499-516.

55. Zimmermann, R., et al., *Protein translocation across the ER membrane.* Biochimica et Biophysica Acta (BBA)-Biomembranes, 2010.

56. YaDeau, J.T., C. Klein, and G. Blobel, *Yeast signal peptidase contains a glycoprotein and the Sec11 gene product.* Proc. Natl. Acad. Sci. U. S. A., 1991. **88**(2): p. 517-521.

57. Lyman, S.K. and R. Schekman, *Interaction between BiP and Sec63p is required for the completion of protein translocation into the ER of Saccharomyces cerevisiae.* The Journal of Cell Biology, 1995. **131**(5): p. 1163.

58. Hamilton, T.G. and G.C. Flynn, *Cer1p, a novel Hsp70-related protein required for posttranslational endoplasmic reticulum translocation in yeast.* Journal of Biological Chemistry, 1996. **271**(48): p. 30610.

59. Tyson, J.R. and C.J. Stirling, *LHS1 and SIL1 provide a lumenal function that is essential for protein translocation into the endoplasmic reticulum.* EMBO J., 2000. **19**(23): p. 6440-6452.

60. Bernstein, M., F. Kepes, and R. Schekman, *Sec59 encodes a membrane protein required for core glycosylation in Saccharomyces cerevisiae.* Molecular and cellular biology, 1989. **9**(3): p. 1191.

61. Heller, L., P. Orlean, and W.L. Adair, *Saccharomyces cerevisiae sec59 cells are deficient in dolichol kinase activity.* Proceedings of the National Academy of Sciences, 1992. **89**(15): p. 7013.

62. Bernstein, M., F. Kepes, and R. Schekman, *Sec59 encodes a membrane protein required for core glycosylation in Saccharomyces cerevisiae.* Molecular and cellular biology, 1989. **9**(3): p. 1191-1199.

63. Kukuruzinska, M. and P. Robbins, *Protein glycosylation in yeast: transcript heterogeneity of the ALG7 gene.* Proceedings of the National Academy of Sciences of the United States of America, 1987. **84**(8): p. 2145.

64. Barnes, G., et al., *Asparagine-linked glycosylation in Saccharomyces cerevisiae: genetic analysis of an early step.* Molecular and cellular biology, 1984. **4**(11): p. 2381-2388.

65. Herscovics, A. and P. Orlean, *Glycoprotein biosynthesis in yeast.* The FASEB journal, 1993. **7**(6): p. 540-550.

66. Bickel, T., et al., *Biosynthesis of Lipid-linked Oligosaccharides in Saccharomyces cerevisiae.* Journal of Biological Chemistry, 2005. **280**(41): p. 34500-34506.

67. Chantret, I., et al., *Two proteins homologous to the N- and C-terminal domains of the bacterial glycosyltransferase Murg are required for the second step of dolichyl-linked oligosaccharide synthesis in Saccharomyces cerevisiae. Vol. 280 (2005) 9236-9242.* Journal of Biological Chemistry, 2005. **280**(18): p. 18551-18552.

68. Gao, X.-D., et al., *Alg14 Recruits Alg13 to the Cytoplasmic Face of the Endoplasmic Reticulum to Form a Novel Bipartite UDP-N-acetylglucosamine Transferase Required for the Second Step of N-Linked Glycosylation.* Journal of Biological Chemistry, 2005. **280**(43): p. 36254-36262.

69. Wilson, I., et al., *Dolichol is not a necessary moiety for lipid-linked oligosaccharide substrates of the mannosyltransferases involved in in vitro N-linked-oligosaccharide assembly.* Biochemical Journal, 1995. **310**(Pt 3): p. 909.

70. Albright, C.F. and R. Robbins, *The sequence and transcript heterogeneity of the yeast gene ALG1, an essential mannosyltransferase involved in N-glycosylation.* Journal of Biological Chemistry, 1990. **265**(12): p. 7042-7049.

71. Gao, X.D., A. Nishikawa, and N. Dean, *Physical interactions between the Alg1, Alg2, and Alg11 mannosyltransferases of the endoplasmic reticulum.* Glycobiology, 2004. **14**(6): p. 559-570.

72. Huffaker, T.C. and P. Robbins, *Temperature-sensitive yeast mutants deficient in asparagine-linked glycosylation.* Journal of Biological Chemistry, 1982. **257**(6): p. 3203-3210.

73. Yamazaki, H., et al., *Characterization of alg2 encoding a mannosyltransferase in the zygomycete fungus Rhizomucor pusillus.* Gene, 1998. **221**(2): p. 179-184.

74. Cipollo, J.F., et al., *The Yeast ALG11 Gene Specifies Addition of the Terminal α1,2-Man to the Man5GlcNAc2-PP-dolicholN-Glycosylation Intermediate Formed on the Cytosolic Side of the Endoplasmic Reticulum.* Journal of Biological Chemistry, 2001. **276**(24): p. 21828-21840.

75. Thiel, C., et al., *A New Type of Congenital Disorders of Glycosylation (CDG-Ii) Provides New Insights into the Early Steps of Dolichol-linked Oligosaccharide Biosynthesis.* Journal of Biological Chemistry, 2003. **278**(25): p. 22498-22505.

76. Cipollo, J.F., et al., *The Yeast ALG11 Gene Specifies Addition of the Terminal α1, 2-Man to the Man5GlcNAc2-PP-dolicholN-Glycosylation Intermediate Formed on the Cytosolic Side of the Endoplasmic Reticulum.* Journal of Biological Chemistry, 2001. **276**(24): p. 21828-21840.

77. O'Reilly, M.K., G. Zhang, and B. Imperiali, *In vitro evidence for the dual function of Alg2 and Alg11: essential mannosyltransferases in N-linked glycoprotein biosynthesis.* Biochemistry, 2006. **45**(31): p. 9593-9603.

78. Helenius, J., et al., *Translocation of lipid-linked oligosaccharides across the ER membrane requires Rft1 protein.* Nature, 2002. **415**(6870): p. 447-450.

79. Imbach, T., et al., *Deficiency of dolichol-phosphate-mannose synthase-1 causes congenital disorder of glycosylation type Ie.* Journal of Clinical Investigation, 2000. **105**(2): p. 233-239.

80. Orlean, P., C. Albright, and P.W. Robbins, *Cloning and sequencing of the yeast gene for dolichol phosphate mannose synthase, an essential protein.* Journal of Biological Chemistry, 1988. **263**(33): p. 17499-17507.

81. Runge, K., T.C. Huffaker, and P.W. Robbins, *Two yeast mutations in glucosylation steps of the asparagine glycosylation pathway.* Journal of Biological Chemistry, 1984. **259**(1): p. 412.

82. Korner, C., et al., *Carbohydrate deficient glycoprotein syndrome type IV: deficiency of dolichyl-P-Man : Man(5)GlcNAc(2)-PP-dolichyl mannosyltransferase.* Embo Journal, 1999. **18**(23): p. 6816-6822.

83. Frank, C.G. and M. Aebi, *ALG9 mannosyltransferase is involved in two different steps of lipid-linked oligosaccharide biosynthesis.* Glycobiology, 2005. **15**(11): p. 1156-1163.

84. Burda, P., et al., *Ordered assembly of the asymmetrically branched lipid-linked oligosaccharide in the endoplasmic reticulum is ensured by the substrate specificity of the individual glycosyltransferases.* Glycobiology, 1999. **9**(6): p. 617.

85. Reilly, M. and T. Doering, *Biosynthesis of fungal and yeast glycans.* Microbial Glycobiology: Structures, Relevance, and Applications, 2009.

86. Stagljar, I., S. Te Heesen, and M. Aebi, *New phenotype of mutations deficient in glucosylation of the lipid-linked oligosaccharide: cloning of the ALG8 locus.* Proceedings of the National Academy of Sciences, 1994. **91**(13): p. 5977.

87. Burda, P. and M. Aebi, *The ALG10 locus of Saccharomyces cerevisiae encodes the α-1, 2 glucosyltransferase of the endoplasmic reticulum: the terminal glucose of the lipid-linked oligosaccharide is required for efficient N-linked glycosylation.* Glycobiology, 1998. **8**(5): p. 455-462.

88. Orlean, P. and A.K. Menon, *Thematic review series: lipid posttranslational modifications. GPI anchoring of protein in yeast and mammalian cells, or: how we learned to stop worrying and love glycophospholipids.* Journal of lipid research, 2007. **48**(5): p. 993-1011.

89. Pittet, M. and A. Conzelmann, *Biosynthesis and function of GPI proteins in the yeast Saccharomyces cerevisiae.* Biochimica et Biophysica Acta (BBA)-Molecular and Cell Biology of Lipids, 2007. **1771**(3): p. 405-420.

90. Watanabe, R., et al., *Mammalian PIG-L and its yeast homologue Gpi12p are N-acetylglucosaminylphosphatidylinositol de-N-acetylases essential in glycosylphosphatidylinositol biosynthesis.* Biochemical Journal, 1999. **339**(Pt 1): p. 185.

91. Umemura, M., et al., *GWT1 gene is required for inositol acylation of glycosylphosphatidylinositol anchors in yeast.* Journal of Biological Chemistry, 2003. **278**(26): p. 23639.

92. Maeda, Y., et al., *PIG-M transfers the first mannose to glycosylphosphatidylinositol on the lumenal side of the ER.* The EMBO Journal, 2001. **20**(1): p. 250-261.

93. Gaynor, E.C., et al., *MCD4 encodes a conserved endoplasmic reticulum membrane protein essential for glycosylphosphatidylinositol anchor synthesis in yeast.* Molecular biology of the cell, 1999. **10**(3): p. 627-648.

94. Kang, J.Y., et al., *PIG-V involved in transferring the second mannose in glycosylphosphatidylinositol.* Journal of Biological Chemistry, 2005. **280**(10): p. 9489-9497.

95. Sütterlin, C., et al., *Saccharomyces cerevisiae GPI10, the functional homologue of human PIG-B, is required for glycosylphosphatidylinositol-anchor synthesis.* Biochemical Journal, 1998. **332**(Pt 1): p. 153.

96. Grimme, S.J., et al., *The essential Smp3 protein is required for addition of the side-branching fourth mannose during assembly of yeast glycosylphosphatidylinositols.* Journal of Biological Chemistry, 2001. **276**(29): p. 27731.

97. Taron, C.H., et al., *Glycosylphosphatidylinositol biosynthesis defects in Gpi11p-and Gpi13p-deficient yeast suggest a branched pathway and implicate gpi13p in phosphoethanolamine transfer to the third mannose.* Molecular biology of the cell, 2000. **11**(5): p. 1611-1630.

98. Ohishi, K., N. Inoue, and T. Kinoshita, *PIG-S and PIG-T, essential for GPI anchor attachment to proteins, form a complex with GAA1 and GPI8.* The EMBO Journal, 2001. **20**(15): p. 4088-4098.

99. Fraering, P., et al., *The GPI transamidase complex of Saccharomyces cerevisiae contains Gaa1p, Gpi8p, and Gpi16p.* Molecular biology of the cell, 2001. **12**(10): p. 3295-3306.

100. Tanaka, S., et al., *Inositol deacylation of glycosylphosphatidylinositol-anchored proteins is mediated by mammalian PGAP1 and yeast Bst1p.* Journal of Biological Chemistry, 2004. **279**(14): p. 14256.

101. Yan, Q. and W.J. Lennarz, *Studies on the Function of Oligosaccharyl Transferase Subunits.* Journal of Biological Chemistry, 2002. **277**(49): p. 47692-47700.

102. Nilsson, I., et al., *Photocross-linking of nascent chains to the STT3 subunit of the oligosaccharyltransferase complex.* The Journal of Cell Biology, 2003. **161**(4): p. 715-725.

103. Heesen, S.T., et al., *YEAST WBP1P AND SWP1P FORM A PROTEIN COMPLEX ESSENTIAL FOR OLIGOSACCHARYL TRANSFERASE-ACTIVITY.* Embo Journal, 1993. **12**(1): p. 279-284.

104. Zufferey, R., et al., *STT3, A HIGHLY CONSERVED PROTEIN REQUIRED FOR YEAST OLIGOSACCHARYL TRANSFERASE-ACTIVITY IN-VIVO.* Embo Journal, 1995. **14**(20): p. 4949-4960.

105. Yan, Q. and W.J. Lennarz, *Oligosaccharyltransferase: A complex multisubunit enzyme of the endoplasmic reticulum.* Biochemical and Biophysical Research Communications, 1999. **266**(3): p. 684-689.

106. Dempski, R.E. and B. Imperiali, *Heterologous expression and biophysical characterization of soluble oligosaccharyl transferase subunits.* Archives of Biochemistry and Biophysics, 2004. **431**(1): p. 63-70.

107. Girrbach, V. and S. Strahl, *Members of the Evolutionarily Conserved PMT Family of ProteinO-Mannosyltransferases Form Distinct Protein Complexes among Themselves.* Journal of Biological Chemistry, 2003. **278**(14): p. 12554.

108. Goder, V. and A. Melero, *Protein O-mannosyltransferases participate in ER protein quality control.* Journal of Cell Science, 2011. **124**(1): p. 144.

109. Girrbach, V. and S. Strahl, *Members of the Evolutionarily Conserved PMT Family of ProteinO-Mannosyltransferases Form Distinct Protein Complexes among Themselves.* Journal of Biological Chemistry, 2003. **278**(14): p. 12554-12562.

110. Esmon, B., P. Esmon, and R. Schekman, *Early steps in processing of yeast glycoproteins.* Journal of Biological Chemistry, 1984. **259**(16): p. 10322.

111. Jakob, C.A., et al., *Degradation of Misfolded Endoplasmic Reticulum Glycoproteins in Saccharomyces cerevisiae Is Determined by a Specific Oligosaccharide Structure.* The Journal of Cell Biology, 1998. **142**(5): p. 1223-1233.

112. Camirand, A., et al., *Glycoprotein biosynthesis in Saccharomyces cerevisiae. Isolation and characterization of the gene encoding a specific processing alpha-mannosidase.* Journal of Biological Chemistry, 1991. **266**(23): p. 15120-15127.

113. Jelinek-Kelly, S. and A. Herscovics, *Glycoprotein biosynthesis in Saccharomyces cerevisiae. Purification of the alpha-mannosidase which removes one specific mannose residue from Man9GlcNAc.* Journal of Biological Chemistry, 1988. **263**(29): p. 14757-14763.

114. Normington, K., et al., *S. cerevisiae encodes an essential protein homologous in sequence and function to mammalian BiP.* Cell, 1989. **57**(7): p. 1223-1236.

115. Rose, M., L. Misra, and J. Vogel, *KAR2, a karyogamy gene, is the yeast homolog of the mammalian BiP/GRP78 gene.* Cell, 1989. **57**(7): p. 1211-1221.

116. Vembar, S. and J. Brodsky, *One step at a time: endoplasmic reticulum-associated degradation.* Nature Reviews Molecular Cell Biology, 2008. **9**(12): p. 944-957.

117. Scidmore, M.A., H.H. Okamura, and M.D. Rose, *Genetic interactions between KAR2 and SEC63, encoding eukaryotic homologues of DnaK and DnaJ in the endoplasmic reticulum.* Molecular biology of the cell, 1993. **4**(11): p. 1145.

118. Schlenstedt, G., et al., *A yeast DnaJ homologue, Scj1p, can function in the endoplasmic reticulum with BiP/Kar2p via a conserved domain that specifies interactions with Hsp70s.* The Journal of Cell Biology, 1995. **129**(4): p. 979-988.

119. Normington, K., et al., *S. cerevisiae encodes an essential protein homologous in sequence and function to mammalian BiP.* Cell, 1989. **57**(7): p. 1223-1236.

120. Steel, G.J., et al., *Coordinated activation of Hsp70 chaperones.* Science, 2004. **303**(5654): p. 98.

121. Gross, E., et al., *Structure of Ero1p, source of disulfide bonds for oxidative protein folding in the cell.* Cell, 2004. **117**(5): p. 601-610.

122. Tu, B.P. and J.S. Weissman, *Oxidative protein folding in eukaryotes: mechanisms and consequences.* J. Cell Biol., 2004. **164**(3): p. 341-346.

123. Frand, A.R. and C.A. Kaiser, *The ERO1 gene of yeast is required for oxidation of protein dithiols in the endoplasmic reticulum.* Molecular cell, 1998. **1**(2): p. 161-170.

124. Rivier, A.S., et al., *Exit of GPI‐Anchored Proteins from the ER Differs in Yeast and Mammalian Cells.* Traffic, 2010. **11**(8): p. 1017-1033.

125. Lederkremer, G., et al., *Structure of the Sec23p/24p and Sec13p/31p complexes of COPII.* Proceedings of the National Academy of Sciences of the United States of America, 2001. **98**(19): p. 10704.

126. Kodera, C., et al., *Sed4p Stimulates Sar1p GTP Hydrolysis and Promotes Limited Coat Disassembly.* Traffic, 2011. **12**(5): p. 591-599.

127. Supek, F., et al., *Sec16p potentiates the action of COPII proteins to bud transport vesicles.* The Journal of Cell Biology, 2002. **158**(6): p. 1029-1038.

128. Gimeno, R., P. Espenshade, and C. Kaiser, *COPII coat subunit interactions: Sec24p and Sec23p bind to adjacent regions of Sec16p.* Molecular biology of the cell, 1996. **7**(11): p. 1815.

129. Supek, F., et al., *Sec16p potentiates the action of COPII proteins to bud transport vesicles.* The Journal of Cell Biology, 2002. **158**(6): p. 1029.

130. Wang, W., M. Sacher, and S. Ferro-Novick, *TRAPP stimulates guanine nucleotide exchange on Ypt1p.* The Journal of Cell Biology, 2000. **151**(2): p. 289-296.

131. Noda, Y., T. Yamagishi, and K. Yoda, *Specific membrane recruitment of Uso1 protein, the essential endoplasmic reticulum‐to‐Golgi tethering factor in yeast vesicular transport.* Journal of cellular biochemistry, 2007. **101**(3): p. 686-694.

132. Hardwick, K.G. and H. Pelham, *SED5 encodes a 39-kD integral membrane protein required for vesicular transport between the ER and the Golgi complex.* The Journal of Cell Biology, 1992. **119**(3): p. 513-521.

133. Newman, A.P., J. Shim, and S. Ferro-Novick, *BET1, BOS1, and SEC22 are members of a group of interacting yeast genes required for transport from the endoplasmic reticulum to the Golgi complex.* Molecular and cellular biology, 1990. **10**(7): p. 3405-3414.

134. Kamena, F., et al., *Ypt1p is essential for retrograde Golgi-ER transport and for Golgi maintenance in S. cerevisiae.* Journal of cell science, 2008. **121**(Pt 8): p. 1293.

135. Dilcher, M., et al., *Use1p is a yeast SNARE protein required for retrograde traffic to the ER.* EMBO J, 2003. **22**(14): p. 3664-3674.

136. Springer, S., A. Spang, and R. Schekman, *A primer on vesicle budding.* Cell, 1999. **97**(2): p. 145.

137. Eugster, A., et al., *COP I domains required for coatomer integrity, and novel interactions with ARF and ARF-GAP.* The EMBO Journal, 2000. **19**(15): p. 3905-3917.

138. Clerc, S., et al., *Htm1 protein generates the N-glycan signal for glycoprotein degradation in the endoplasmic reticulum.* The Journal of Cell Biology, 2009. **184**(1): p. 159.

139. Gauss, R., et al., *A complex of Pdi1p and the mannosidase Htm1p initiates clearance of unfolded glycoproteins from the endoplasmic reticulum.* Molecular cell, 2011. **42**(6): p. 782-793.

140. Xie, W. and D.T.W. Ng, *ERAD substrate recognition in budding yeast.* Seminars in Cell &amp; Developmental Biology, 2010. **21**(5): p. 533-539.

141. Gauss, R., et al., *A complex of Yos9p and the HRD ligase integrates endoplasmic reticulum quality control into the degradation machinery.* Nature cell biology, 2006. **8**(8): p. 849-854.

142. Szathmary, R., et al., *Yos9 protein is essential for degradation of misfolded glycoproteins and may function as lectin in ERAD.* Molecular cell, 2005. **19**(6): p. 765-775.

143. Lilley, B. and H. Ploegh, *A membrane protein required for dislocation of misfolded proteins from the ER.* Nature, 2004. **429**(6994): p. 834-840.

144. Kostova, Z. and D.H. Wolf, *For whom the bell tolls: protein quality control of the endoplasmic reticulum and the ubiquitin-proteasome connection.* EMBO J, 2003. **22**(10): p. 2309-2317.

145. Huyer, G., et al., *Distinct machinery is required in Saccharomyces cerevisiae for the endoplasmic reticulum-associated degradation of a multispanning membrane protein and a soluble luminal protein.* Journal of Biological Chemistry, 2004. **279**(37): p. 38369-38378.

146. Akiyama, Y. and K. Ito, *Reconstitution of membrane proteolysis by FtsH.* Journal of Biological Chemistry, 2003. **278**(20): p. 18146-18153.

147. Carvalho, P., V. Goder, and T.A. Rapoport, *Distinct ubiquitin-ligase complexes define convergent pathways for the degradation of ER proteins.* Cell, 2006. **126**(2): p. 361-373.

148. Nakayama, K., et al., *OCH1 encodes a novel membrane bound mannosyltransferase: outer chain elongation of asparagine-linked oligosaccharides.* The EMBO Journal, 1992. **11**(7): p. 2511.

149. Lehle, L., et al., *Glycoprotein biosynthesis in Saccharomyces cerevisiae: ngd29, an N-glycosylation mutant allelic to och1 having a defect in the initiation of outer chain formation.* FEBS letters, 1995. **370**(1-2): p. 41-45.

150. Stolz, J. and S. Munro, *The Components of the Saccharomyces cerevisiaeMannosyltransferase Complex M-Pol I Have Distinct Functions in Mannan Synthesis.* Journal of Biological Chemistry, 2002. **277**(47): p. 44801-44808.

151. Lussier, M., et al., *The Ktr1p, Ktr3p, and Kre2p/Mnt1p Mannosyltransferases Participate in the Elaboration of Yeast O-andN-linked Carbohydrate Chains.* Journal of Biological Chemistry, 1997. **272**(24): p. 15527.

152. Häusler, A., et al., *Yeast glycoprotein biosynthesis: MNT1 encodes an alpha-1, 2-mannosyltransferase involved in O-glycosylation.* Proceedings of the National Academy of Sciences, 1992. **89**(15): p. 6846.

153. Lussier, M., et al., *The Ktr1p, Ktr3p, and Kre2p/Mnt1p Mannosyltransferases Participate in the Elaboration of Yeast O-andN-linked Carbohydrate Chains.* Journal of Biological Chemistry, 1997. **272**(24): p. 15527-15531.

154. Cooper, A. and H. Bussey, *Characterization of the yeast KEX1 gene product: a carboxypeptidase involved in processing secreted precursor proteins.* Molecular and cellular biology, 1989. **9**(6): p. 2706.

155. Odorizzi, G., C.R. Cowles, and S.D. Emr, *The AP-3 complex: a coat of many colours.* Trends in cell biology, 1998. **8**(7): p. 282-287.

156. Bonifacino, J.S., *The GGA proteins: adaptors on the move.* Nature Reviews Molecular Cell Biology, 2004. **5**(1): p. 23-32.

157. Dell'Angelica, E.C., et al., *AP-3: an adaptor-like protein complex with ubiquitous expression.* The EMBO Journal, 1997. **16**(5): p. 917-928.

158. Harsay, E. and R. Schekman, *A subset of yeast vacuolar protein sorting mutants is blocked in one branch of the exocytic pathway.* J. Cell Biol., 2002. **156**(2): p. 271-285.
