# Supplementary material for: Genome-Scale Modeling of the Protein Secretory Machinery in Yeast
Source: PLoS One. 2013 May 7;8(5):e63284. doi: 10.1371/journal.pone.0063284 (PMC3646752; doi:10.1371/journal.pone.0063284)
Supplement: Text S1 — The reconstruction approach. The reconstruction approach and assumption of the model is described in details in Text S1. (DOCX) [file pone.0063284.s019.docx]

# Genome-Scale Modeling of the Protein Secretory Machinery in Yeast

**Amir Feizi^1^, Tobias Österlund^1^, Dina Petranovic^1^, Sergio Bordel^1^, Jens Nielsen^1,2*^**

1 Novo Nordisk Foundation Center for Biosustainability, Chalmers University of Technology, Kemivägen 10, SE412 96 Gothenburg, Sweden

2 Novo Nordisk Foundation Center for Biosustainability, Technical University of Denmark, DK2870 Hørsholm, Denmark

*corresponding author contact information:

Table of Contents

[Genome-Scale Modeling of the Protein Secretory Machinery in Yeast 1](#_Toc347330655)

[An example for template reaction list: 3](#_Toc347330656)

[Model subsystems and assumptions 4](#_Toc347330657)

[Co and post-translational translocation 4](#_Toc347330658)

[Transmembrane proteins 5](#_Toc347330659)

[Machinery protein complexes 5](#_Toc347330660)

[ER Glycosylation 6](#_Toc347330661)

[Protein folding and ER quality control (ERQC) 6](#_Toc347330662)

[Transport and vesiculation 8](#_Toc347330663)

[Golgi glycosylation 10](#_Toc347330664)

[Polypeptide chain cleavage 10](#_Toc347330665)

[Post Golgi subsystems 10](#_Toc347330666)

[Supplementary Figures 12](#_Toc347330667)

[Supplementary Tables 16](#_Toc347330668)

[References 21](#_Toc347330669)

## An example for template reaction list:

The formulation of the template reactions falls into the third step of the reconstruction process. The reactions include all the mechanistic knowledge of the secretory machinery in the literature and databases. We explain part of the translocation subsystem’s template reaction lists here as an illustration.

Secretory proteins first need to enter to the ER in order to be properly folded, get post-transcriptional modifications and be directed to its final location in the cell[1, 2]. Translocation enters the target protein to the ER[3]. Figure 2, represents the translocation steps to the ER. The first 7 template reactions for a candidate protein (*XXX*) have been shown below. First, the nascent protein with signal peptide (SP) is detected by the signal peptide recognition (SRP) complex (reaction 1) and then it binds the signal recognition receptor on the ER membrane (SRP-receptor) (reaction 2) [4]. By dephosphorylating 2 GTP molecules this complex introduces the nascent peptide in the *translocon* channel on the ER (reaction 3)[5].Then, the SRP and it is receptor are detached from the nascent protein and are free to get involved in another recognition round. The target protein passes through the channel as it is being translated; meanwhile the signal peptidase enzyme cleaves the signal peptide from the N-terminal end of the protein (reaction 4). Finally, the signal peptide is degraded (reactions 5) (For full list of the template reactions list see the supplementary Table S3).

**Template reaction list**

**[1]** XXX[Cytoplasm] + SRPC[Cytoplasm] => XXX-SRPC[Cytoplasm]

**[2]** XXX-SRPC[Cytoplasm] + SRC[Endoplasmic Reticulum] => XXX-SRPC-SRC[Cytoplasm] [3]XXX-SRPC-SRC[Cytoplasm] + 2 GTP[Cytoplasm] + SEC61C[Endoplasmic Reticulum] => XXX- SRPC-SRC-SEC61C[Cytoplasm]

**[3]** XXX-SRPC-SRC-SEC61C[Cytoplasm] =>XXX-SEC61C[Cytoplasm] + Srp14p[Cytoplasm] + Srp21p[Cytoplasm] + Srp68p[Cytoplasm] + Sec65p[Cytoplasm] + Srp72p[Cytoplasm] + scR1[Cytoplasm] + Srp54p[Cytoplasm] + Srp51p[Cytoplasm] + Srp52p [Cytoplasm] + 2GDP [Cytoplasm] + 2 pi [Cytoplasm]

**[4]** XXX-SEC61C[Cytoplasm] + SPC[Endoplasmic Reticulum] => XXX[Endoplasmic Reticulum] + Sec61p[Endoplasmic Reticulum] + Sbh1p[Endoplasmic Reticulum] + Sss1p[Endoplasmic Reticulum] + Sec11p[Endoplasmic Reticulum] + Spc1p[Endoplasmic Reticulum] + Spc2p[Endoplasmic Reticulum] + Spc3p[Endoplasmic Reticulum] + sp[Cytoplasm]

**[5]** sp[Cytoplasm] => ac[Cytoplasm]

**Translocation process**


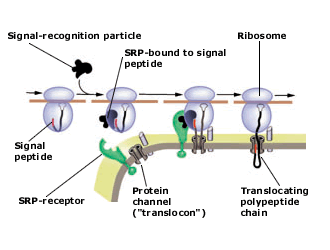


Figure 1- Formulation of the translocation subsystem in terms of template reactions.

The same procedure has been applied to all the subsystems to reach a final list of template reactions (supplementary Table S3).

## Model subsystems and assumptions

### Co and post-translational translocation

- There are two main protein translocation mechanisms, Co- and post-transnational translocations, both of which are believed to occur in *Saccharomyces cerevisiae*. [2, 6]. The hydrophobicity in the *h-region* of the tripartite structure of the *N*-terminal signal peptide allows the protein’s nascent chain to either bind the SRP (signal recognition particle) complex for high hydrophobicity or use the post-translational translocation for lower hydrophobicity [7] [8] [9](Figure 8). It has for example been hypothesized that the post-translational mechanism seems to be the choice for precursors of GPI-anchored membrane proteins, such as variant surface glycoprotein (VSG) [10]. However there is not any database containing information on the type of the translocation mechanism for each specific protein. Therefore, we assumed all the proteins containing signal peptides to use the co-translational translocation mechanism.
- It has been shown that the SRP complex formation occurs in three steps: first *SRP14p*, *SRP21p*, *SRP68p*, *SRP72p*, *SEC65*, and *SRP54*, and the RNA (termed *scR1* and encoded by *SCR1*) are imported into the nucleolus and assembled together; second, the assembled core SRP is released from the nucleolus to the nucleoplasm to bind to the *Sec65p*, third the particle is exported to the cytoplasm, where it binds the *importin-β* homologue *Xpo1p/Crm1p* and the *Srp54p* [11-13]. We did not include this mechanism in the model and simply wrote a reaction including the entire SRP complex.
- It has been shown that the ER translocation is reversible[14] .This means that binding of chaperones such as *Kar2p*,*Pdip*,*OST* (complex protein) and other protein complex can be necessary to drive the translocation process in its right sense [15]. For simplicity and due to the lack of definitive evidence we have ignored the role of the non-translocon proteins in translocation.

### Transmembrane proteins

- Translocation process is mechanistically different between transmembrane and luminal (or and secretory proteins). For transmembrane proteins the translocation is stopped and lateral insertion in the membrane occurs. Since there are various kinds of transmembrane proteins, the mechanisms also are different for each type [16]. For each type, there are experimental data declaring that shows specific mechanism of integration [16-18]. Here, we assumed that the integration process is part of the translocation of transmembrane proteins and is energetically costless.
- Each type of transmembrane protein(single or multiple span type I,II and III,) has it is own orientation depending on the presence of the C or N-terminal domains in the ER lumen or cytoplasm respectively [19]. Folding and processes such as glycosylation or disulfide bond formation is dependent on this orientation and can be catalyzed using enzymes in the ER or the cytoplasm.

### Machinery protein complexes

- We formulated complex formation reactions based on the mechanistic knowledge from literature (supplementary Table S3). For more experimental support, we used the list of the yeast protein complexes (491 complexes) defined by Gavin *et al* (2006) to support the stoichiometry our complex formation reactions [20]

### ER Glycosylation

- There are different factors affecting the glycosylation process, like the amino acid composition around the glycosylation site, the position and the distance of different glycosylation sites, and the availability of the dolichol precursors [21-23]. It is not possible to precisely predict the exact pattern of glycosylation for each protein; therefore we assumed that all retrieved glycosylation sites for each protein from UniProt have the same probability of being glycosylated.
- The *dolichol* pathway starts at the cytoplasm face with the using *UDP-GlcNAc* and *GDP-Man* as glycosyl donors and ends with in the ER lumen side by using the *Dol-P-Man* and *Dol-P-Glc* [24-26]. All the enzymes in dolichol pathway are ER membrane proteins and well characterized in yeast [25, 27] (see supplementary table S3 for biosynthesis reaction of dolichol pathway) we assumed all the reactions to be irreversible and accrue in the lumen side to avoid cytoplasm in compartments.. As, in reality not all the glycosylation sites are glycosylated, the reported values can be overestimated (Figure 6 B and C in the paper).

### Protein folding and ER quality control (ERQC)

Three types of Endoplasmic Reticulum-associated Degradation (Luminal ,ERADL; transmembrane, ERADM; cytosolic ERADC, ERAD C) pathways have been proposed as ER quality control system to detect and degrade the mis-folded protein to ensure the fidelity of proper folding., [[30-34](#_ENREF_30)]. It has been also been shown that large amounts of the wild-type proteins can be degraded by the ERAD pathway [[35](#_ENREF_35)]. This shows how stringent the folding machinery is in order guarantee proper folding [[36](#_ENREF_36)].

- The folding cost in ER includes the amount of the ATP that KAR2P and its co-chaperones hydrolyzes through each folding cycle. The number of the folding cycle and consequently the energy cost, depends on the physicochemical factors such as redox homeostasis and it is proteins specific [[37](#_ENREF_37)]. Due to the limited knowledge,For the proteins accommodated in the same secretory classes,we assumed that the folding cost (ATP hydrolysis) is the same.

It has been shown that folding enzymes such as *KAR2* are highly concentrated in the ER in a millimolar range [[38](#_ENREF_38)]., . We estimated that in average, 5 copies of *Kar2p* are involved in the folding of each protein. We got this number from the following formula: $Kar2p_{avreage}=\frac{\left( {468}^{\mathrm{aa}}\times{4.3}^{Å} \right)}{{400}^{Å}}$ where, the nominator is the average length of secretory proteins and the denominator is the diameter of the Kar2p protein complex [[39](#_ENREF_39)]. This number can be overestimated as the nascent poly peptide chain is not precisely linear and *Kar2p* only binds to the hydrophobic patches [[19](#_ENREF_19)].

- Proteins containing the *Man9-GlcNAc2* residuum are bound by an α-1;2-mannosidase (*MNS1*) and an *α-mannosidase*-like protein (*HTM1/MNL1*), which remove two mannose residues resulting in a *Man7-GlcNAc2* glycan structure[[40](#_ENREF_40), [41](#_ENREF_41)] (Supplementary Table S2). This cleavage step is slow and can be carried on more efficiently if the protein is properly folded. Only after the cleavage the proteins can leave ER lumen to the Golgi. This results in a longer retention time in the ER for mis-folded proteins and increases their probability of entering the degradation pathway. We we ignonerd the multiple folding cycle that can happen due to the retention time.
- The proteins which can be subjected to ERAD pathways are glycoproteins, and it is unclear how non-glycosylated proteins undergo to these three types of ERAD pathway [[42](#_ENREF_42), [43](#_ENREF_43)]. For these proteins we assumed the basic core of the ERAD pathway to be active, which results in similar estimations of the folding ATP costs than those of glycosylated proteins (supplementary Table S2).
- The rate of ERAD pathways for each protein is not known. In contrast to the spontaneous errors that occurs during transcription or translation or mutation, protein folding and consequently the ERAD pathway degradation rateare dependent on the environmental stress. We assumed the degradation rate to be 0.1 for all of the subjected proteins to not overestimate the degradation cost.
- UPR is a regulatory circuit which regulates the expression of the genes corresponding to the mis-folded protein stress in the ER [[44](#_ENREF_44), [45](#_ENREF_45)]. It has been shown that the ERAD components are mostly up-regulated by the UPR under stressful conditions in order to clean the ER from mis-folded proteins. Consequently, UPR increases the folding capacity and regulates the oxidative condition of the ER [[46](#_ENREF_46), [47](#_ENREF_47)].We exclude UPR form our model its activity will eventually change the fraction of proteins being degraded (which we assumed to be 0.1 under non stressed conditions).

### Transport and vesiculation

After the proper folding state, the proteins leave the ER to the Golgi (except ER lumen or membrane resident proteins). Many of the of the major component and mechanisms of the cargo detection, vesiculation and fission have been detected and characterized, however there are many questions to be resolved in terms of regulation and control of the transport [[48-51](#_ENREF_48)].

- There are two known ways for exporting the secreted proteins from the ER to the Golgi
  - Non-specific (passive); bulk-flow; unconventional route.
  - Signal dependent (active); COPII [[52](#_ENREF_52)].

It has been shown that the bulk-flow route is non-specific and its role is negligible [[53](#_ENREF_53), [54](#_ENREF_54)]. It has been also shown that the vesicles budding from the ER can bypass the Golgi and go directly to their final location. For example, the yeast multi-span membrane protein *IST2* has been shown to use a Golgi-independent route to reach the membrane [[55](#_ENREF_55)]. But the alternative secretory pathways are not fully resolved and there are many knowledge gaps in the mechanistic details and component of these pathways [[55](#_ENREF_55), [56](#_ENREF_56)]. While we ignored the alternative secretory pathway, the linearization of COPII vesiculation mechanism also requires many simplifications.

- There are two main mechanisms for cargo detection at the ER transition site: direct binding of the cargo to the coat subunits and adaptor depended mechanism. Most of the transmembrane cargos are supposed to interact directly with the core subunits of the COPII coat complex. On the other hand, most of the soluble cargos and some of the transmembrane and GPI-anchored proteins use transmembrane proteins as adaptors to indirectly bind to the COPII subunits [[57-61](#_ENREF_57)]. We included the current knowledge of the receptor and adaptor proteins which are dealing with cargo selection, vesiculation and fission to the target membrane [[60](#_ENREF_60), [62](#_ENREF_62), [63](#_ENREF_63)](supplementary table S3). However, still there are many knowledge and protein-specific mechanisms to distinguish the cargo from other ER resident proteins remain to be discovered [[50](#_ENREF_50), [62](#_ENREF_62)].
- In contrast to the mammalian ER with a specific site for vesiculation (called ERES) *S.cerevisiae* vesicular budding do not have a certain sub-domain within the membrane and occurs through the whole ER membrane [[64](#_ENREF_64)].Therefore, we excluded any spatial vesiculation.
- There is no enough information about the composition of the COPII vesicles to assess their degree of cargo specificity. On the other hand, some biochemical data have revealed that transmembrane and GPI-anchored proteins differ in abundance in different populations of COPII derived vesicles in yeast [[65](#_ENREF_65)].We did not include any assumption about the cargo COPII composition.
- Some proteins need to form a quaternary multimeric complex with other subunits to be functional and experimentally it has been shown that having the final multi-subunit form is critical in cargo selection by COPII protiens, but it remains to be show how this mechanisim works [[66](#_ENREF_66), [67](#_ENREF_67)] and we did not include the multi-subunit role in selection by cargo receptors in ER.
- Obviously, the size of the COPII should be variable according to the size of cargo and each COPII usually contians more than a protien [[68](#_ENREF_68)]. Also, it is speculated that the size of the vesicules can be dependent on the load of the secretion and upregulated secretion might affect the size of the vesicules [[63](#_ENREF_63)].For the sake of simplicity we ignored the structural and physical constrains on the formation of COPII vesicles.
- The fission step in vesicles transport is a critical and specific process to direct different vesicles to their target compartments. In eukaryotes, SNAREs (soluble N-ethylmaleimide-sensitive factor attachment protein receptors) are key components of the membrane fusion machinery [[69](#_ENREF_69)]. In fact, there are two types of SNARE’s, namely v (vesicles )- and t (target)-SNAREs [[70](#_ENREF_70), [71](#_ENREF_71)]. We assumed the better characterized SNARE to be used for all the vesicles, even if there are other known SNAREs (see the Supplementary table S2 ).
- The role of the homotypic fusion and the role of the microtubules in transport are likely to be less significant in yeast due to the shorter distances along which transport carriers must travel to reach their destination [[72](#_ENREF_72), [73](#_ENREF_73)].
- COPI is the second type of vesicles transferred between the ER and the Golgi. They travel in the oposite sense than COPII [[74](#_ENREF_74)], that’s from the Golgi to the ER. COPI vesicules are responsible for keeping the ER membrane in balance and also return ER luminal or membrane resident proteins that have escaped from ER to the Golgi [[63](#_ENREF_63)]. The problems in formulation of the COPII vesicules also apply for the COPI vesicles. We applied the same stratagy in formulation of the COPI mechanisim (Suplementary Table S2). The only additional issue which is specific to the COPI vesicles is that there is not enough information for the rate of the ER resident protiens escaped to the Golgi apparatus. We assumed that all the ER resident proteins have the possibility to escape from the ER to Golgi (and come back in a COPI vesicule) and they do it once in their life span.

### Golgi glycosylation

In *S.cerevisiae* Golgi apparatus is not a so well-organized structure as in higher eukaryotes [[75](#_ENREF_75)]. The main post-translational modification that occurs in the Golgi apparatus is glycosylation which happens for the *N*-linked and *O-*linked sites of the proteins [[76](#_ENREF_76)].

- In the Golgi apparatus, there are two kinds of manosylation (*N*-linked and *O-*linked) so if a protein has two types of glycosylation sites it needs to undergo two kind of manosylations in the Golgi, but there is no information about the order of these two types of glycosylation. We assumed that if a protein has both kinds of glycosylation sites it goes through both types of glycosylation.
- It has been shown that Golgi enzymes are able to add up to 40 mannoses for *N*-linked and 5 mannoses for *O-*linked glycosylation sites, but in some cases it has been shown that some of the secretory proteins (specially proteins which have a role in the yeast cell wall biogenesis) carry an extensive mannose chain in their *N*-glycosylation site which it supposed to be added in the Golgi apparatus, but there is no information about the signal or exact enzymes which are responsible for extensive mannosylation in the Golgi, consequently we excluded this extra mannosylation steps from the model.

### Polypeptide chain cleavage

The last protein modification in the Golgi before reaching the functional state is the polypeptide chain cleavage. Three Golgi resident peptidases are responsible for the polypeptide chain cleavage based on different sequence motifs. We did not include this step in our template reactions because we could not find any information for each of the proteins cleavage site [[77-79](#_ENREF_77)].

### Post Golgi subsystems

After completing the Golgi processing step, mature proteins need to be sorted to their final destinations. The vesicle transport has been shown to be very rapid while it is very selective. Apparently the post-Golgi sorting step is very critical to direct each protein to its accurate functional location. The main locations are: plasma membrane, early endosome, extracellular space, vacuole, retrograde to ER. The post-Golgi sorting mechanisms are well studied in yeast on proteins with different locations and many of the temperature sensitive mutations resulted in detection of the main component of each of the trafficking routes [[80-84](#_ENREF_80)].

- The main challenges for the post-Golgi reactions are that the sorting sequence motifs and protein receptors have not been discovered for all the machinery clients. We included in the post-Golgi transport mechanism the main components identified through temperature sensitive mutations (See supplementary table S3).

# Supplementary Figures

**
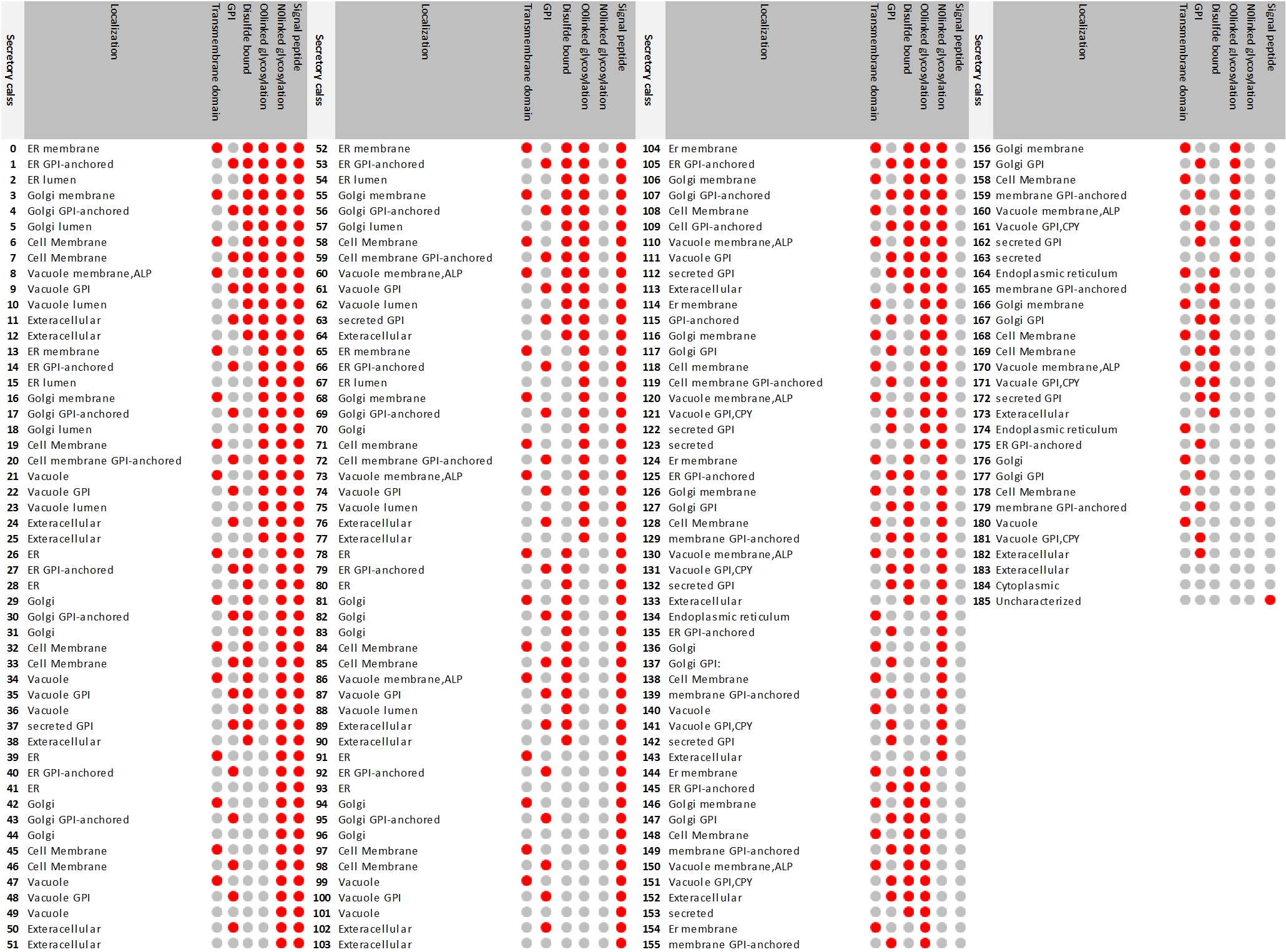
**

**Supplementary Figure S1.** **All of the defined secretory classes for yeast secretory machinery.** The 186 defined secretory classes (starts from class 0 to class 185) with their specific feature combinations. The red spot shows the existence of a feature and gray spot indicates absence. The first 104 class are the classes with signal peptide and the remaining 82 are without signal peptide. The class ids are depicted in the secretory class column. Features description is given at top of each feature column.


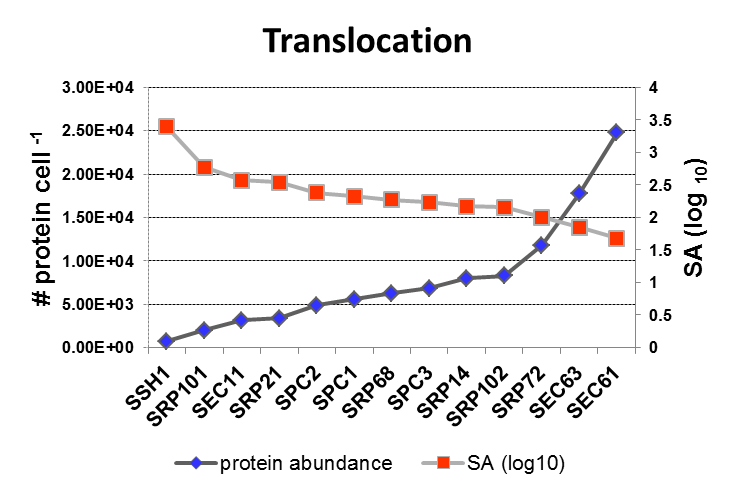

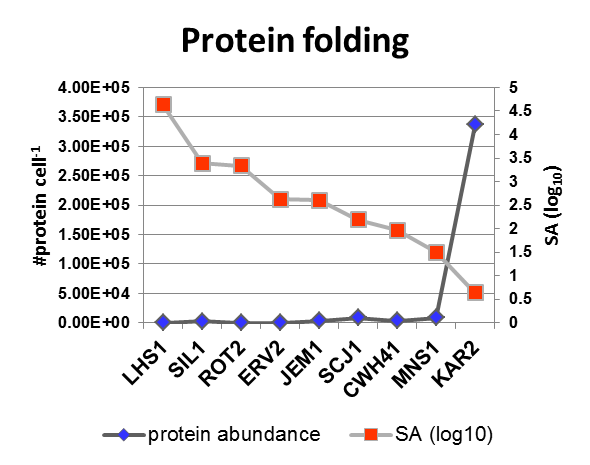

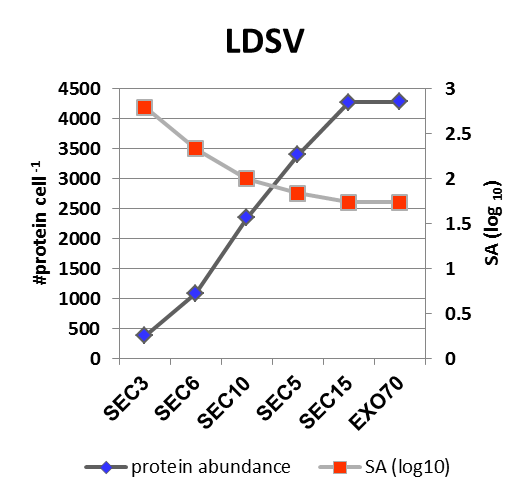

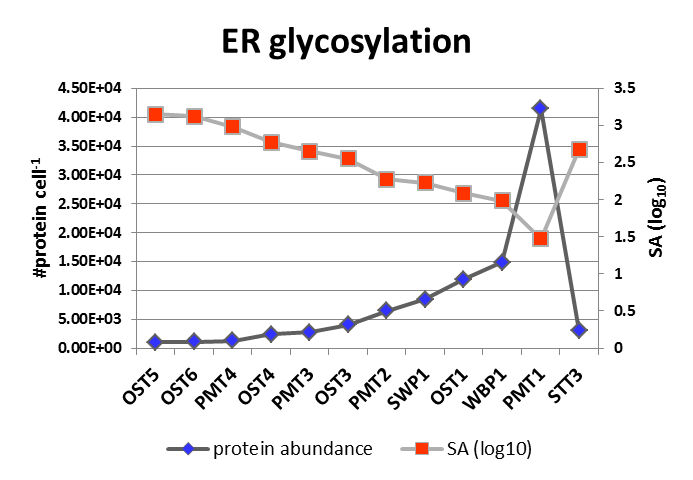

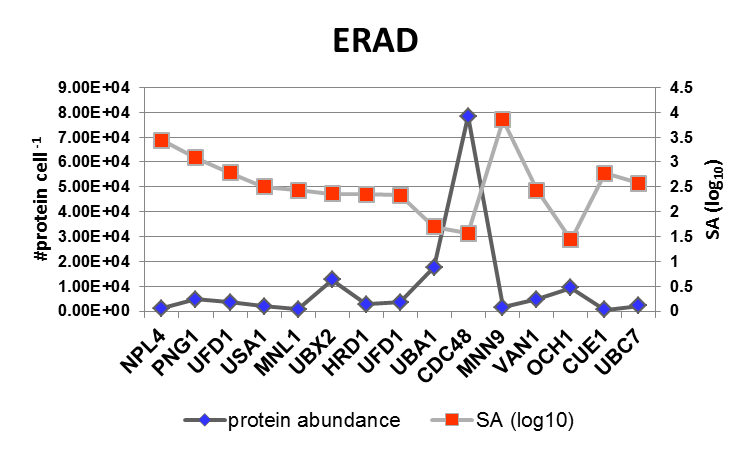

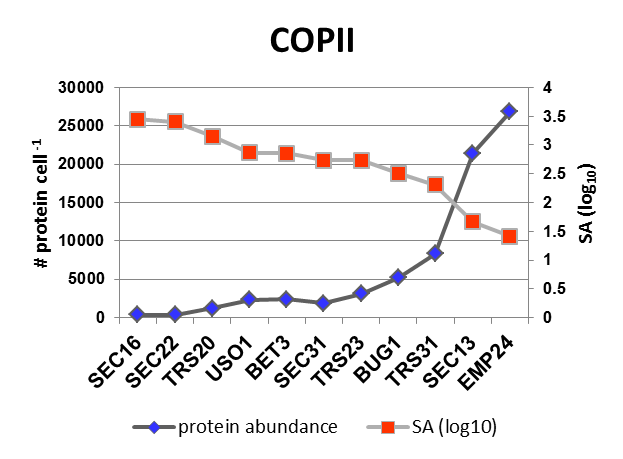

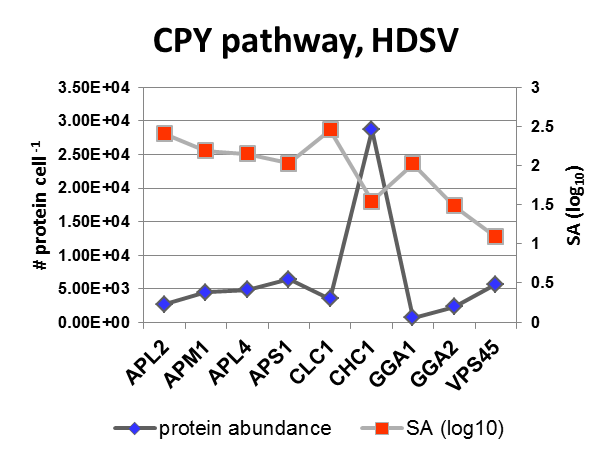

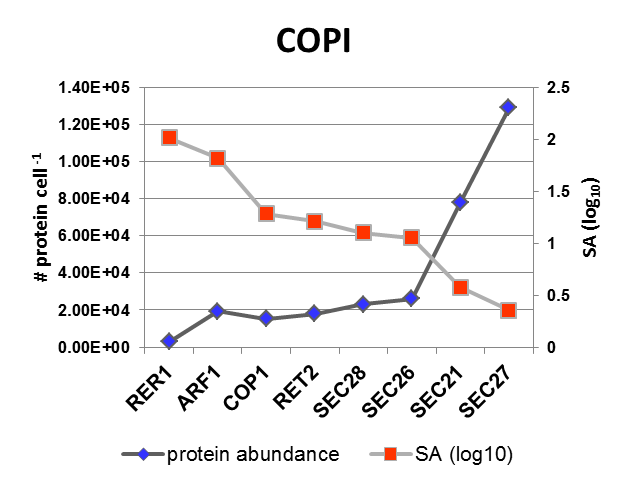

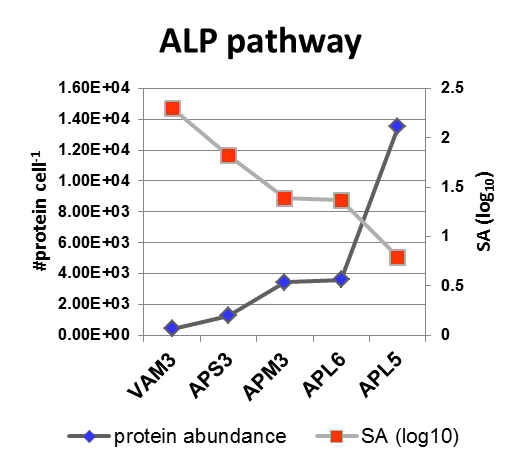

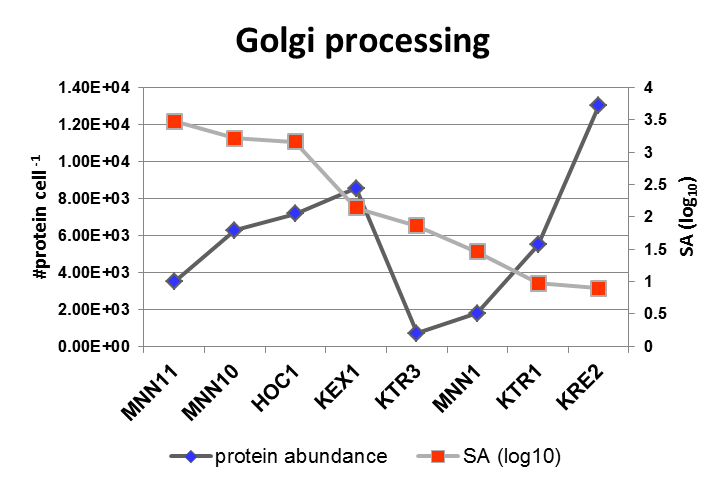

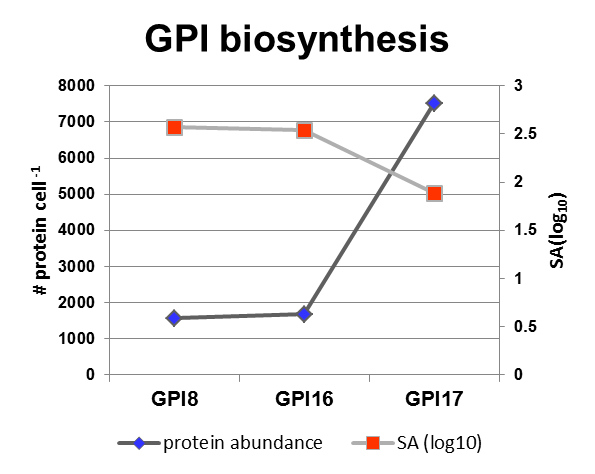


**Supplementary Figure S2.The correlation of the main component of the secretory machinery specific activity(SA) and protein abundance**.The yy-plots for the SA(log _10_)(cell-^1^ h^-1^) and corresponding protein abundance(molecules cell^-1^) of each of the subsystems is shown. The subsystem names are located above each plot.


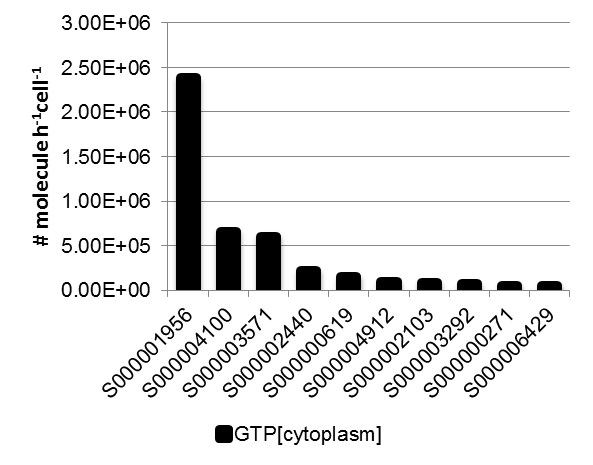

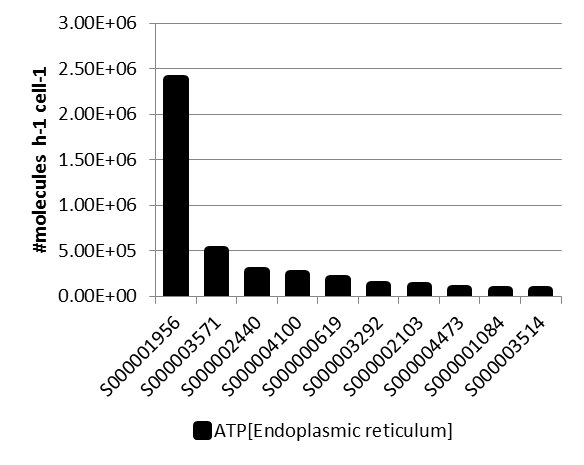

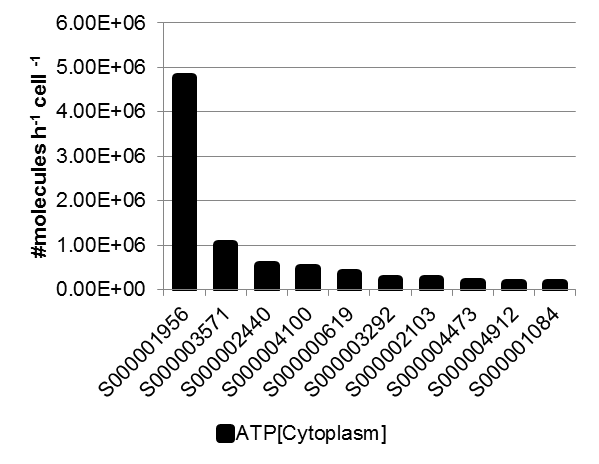

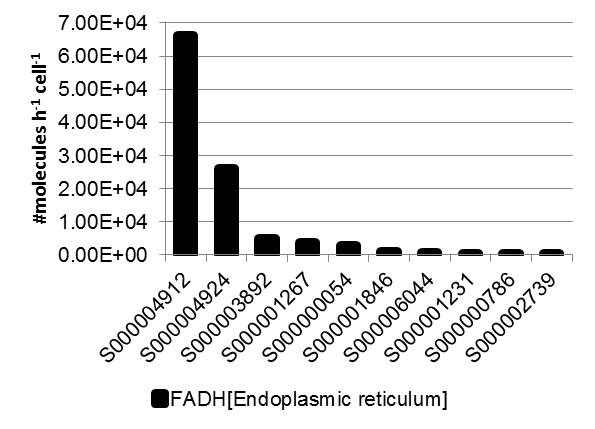

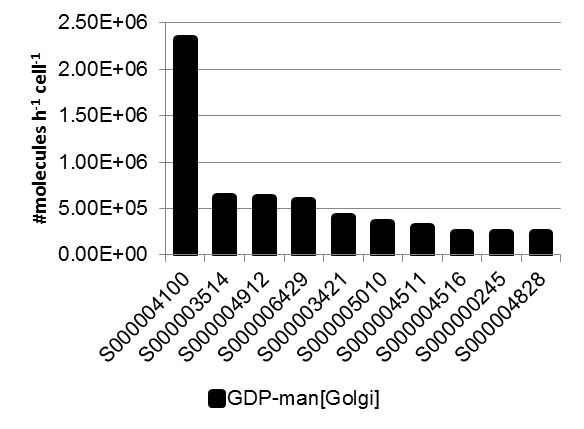

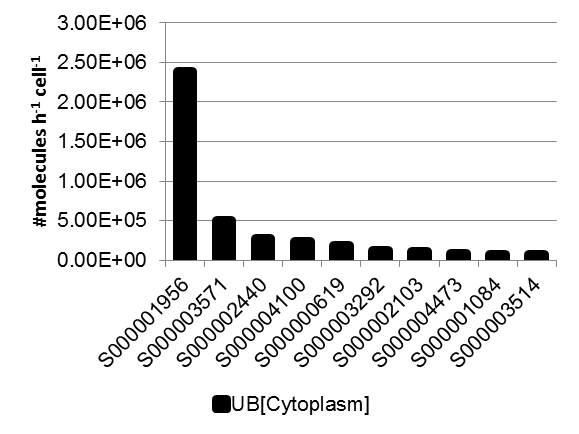

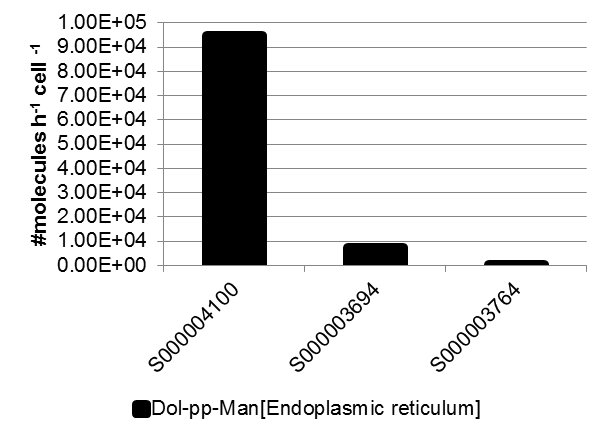

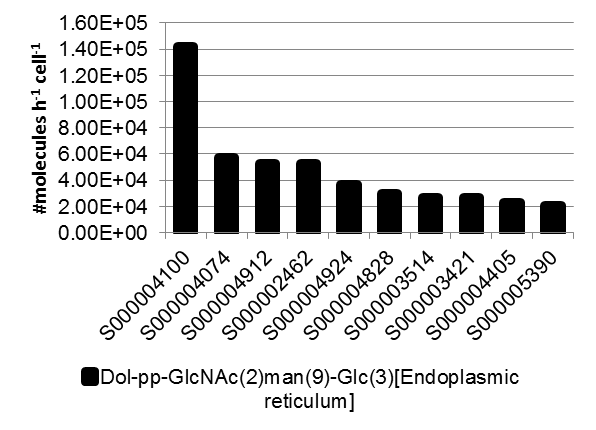

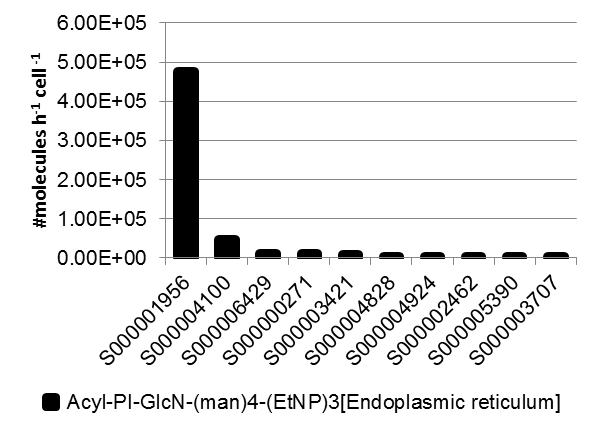

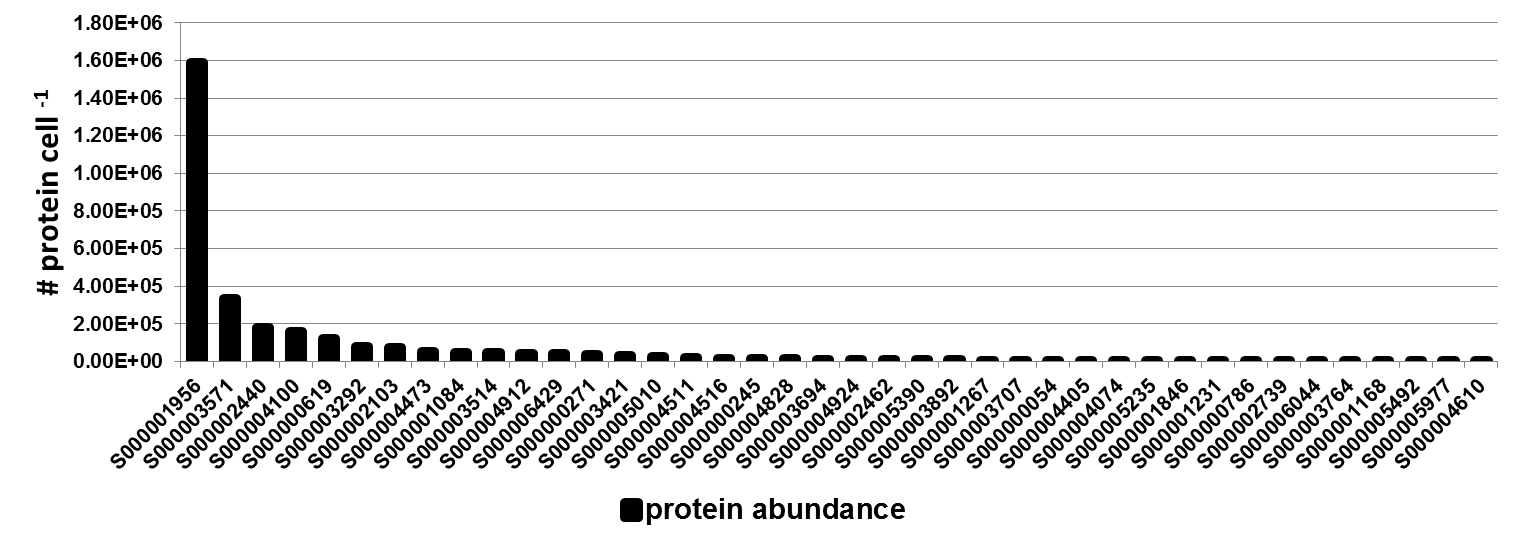


**Supplementary Figure S3. The most metabolic demanded proteins of the secretory machinery.** For each of the metabolic precursors (shown at the bottom of each plot) the top 5 proteins are plotted. For the annotation of these proteins see the Supplementary Table S2. The bottom plot shows the abundance distribution of the highly demanded proteins.


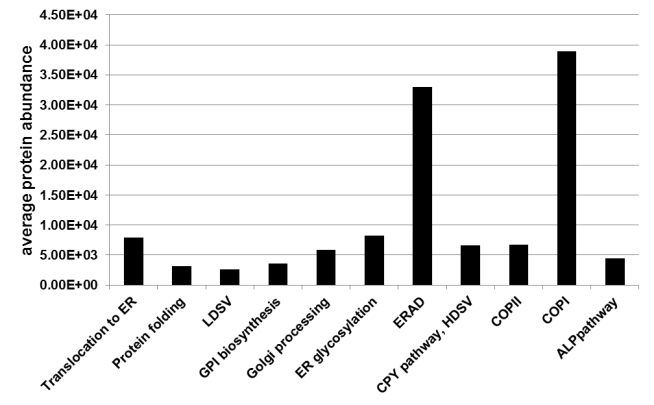


**Supplementary Figure S4. Average abundance of the yeast secretory machinery subsystems component.**

# Supplementary Tables

**Supplementary Table 1.The model exchange metabolites.**

| Metabolite | type | Subsystem |
| --- | --- | --- |
| ATP | energy carriers | folding |
| CTP | Phosphate donor | Dolichol pathway |
| UDP-Glc | Glucose donor | Dolichol pathway |
| GDP-mannose | glycan chain biosynthesis building blocks | GPI biosynthesis, Dolichol pathway, Golgi processing |
| Palmitoyl-CoA | Palmitoyl donor | GPI biosynthesis |
| EtNP (ethanolaminephosphate) | Lipid donor | GPI biosynthesis |
| Dolichol | glycan chain biosynthesis building blocks | GPI biosynthesis, Dolichol pathway |
| UDP-GlcNAC | glycan chain biosynthesis building blocks | GPI biosynthesis, Dolichol pathway |
| Ubiquitin | Protein degradation | ERAD(L,M and C) |
| FADH | electron carrier | folding |
| O2 | Electron acceptor | folding |
| H2O | Electron carrier | folding |

| Subsystem | Name of the subsystem | components | Description |
| --- | --- | --- | --- |
| S1 | Translocation | Co-translocation translocation (***Srp14p,Srp21p,Srp68p,Srp72p,Srp65p,ScR1,Srp101p,Srp102p,Sec61p,Sbh1p,Sss1p,Sec11p,Spc1p,Spc2p,Spc3p,Srp54p,Sss1p,Ssh1p***)  Post-translational translocation ( ***Sbh1p,Sss1p,Ssh1p,Sec63p,Sec62p,Sec66p,Sec72p,Sec61p,Kar2pp,Npl1p***  ) | Simultaneous translocation of the proteins with signal peptide from cytoplasm to the ER through translational process.  Translocation of the protein with signal peptide from cytoplasm to the ER after translation completion |
| S2 | Dolichol pathway | ***Sec59p, Alg7p, Alg13p/14p, Alg1p, Alg11p, Rft1p, Dpm1p, Alg5p, Alg3p, Alg9p, Alg12p, Alg8p, Die2p*** | The pathway for the biosynthesis of the oligosaccharide donor in ER. |
| S3 | **ER glycosylation** | ***Ost1p, Ost2p, Ost3p, Ost4p, Ost5p, Ost6p, Stt3p, Swp1p, Wbp1p***,  PmtC(***Pmt2p,Pmt5P,Pmt1P,Pmt6p,Pmt4p,Pmt3p)*** | The processes needed for glycan transfer (N-linked and O-linked) in the ER to the proteins containing the corresponding glycosylation site. |
| **S4** | Protein folding | ***Cwh41p,Rot2p,Mns1p,Kar2p,Cne1p,Pdi1p,Scj1p,Jem1p,Snf4p,Lhs1p,Sil1p,Eps1p,Ero1p,Kar2p,Lhs1p,Sil1p,Eps1,Erv2p*** | The reactions involved in proper protein folding and disulfide bound formation. |
| S5 | GPI biosynthesis | ***Gpi1p,Gpi2p,Spt14p,Gpi15p,Gpi19p,Eri1p,Gpi12p,Gwt1p,Gpi14p,Mcd4p,Gpi5p,Gpi1p,Smp3p,Gpi13p,Gpi17p,Gaa1p,Gpi8p,Gpi16p,Gab1p,Bst1p*** | Glycosylphosphatidylinositol(GPI) biosynthesis pathway |
| S6 | GPI transfer | ***Gaa1p,Gpi8p,Gpi16p,Gpi17p,Gab1p,Bst1p*** | Transfer the GPI to the GPI site on the proteins. |
| S7 | ERADC | ***Sbh1p, Sss1p,Ssh1p,Ubx2p,Cdc48p,Ufd1p,Npl4p,Doa1p,Png1p***, 26sproteosome | The processes for detection, retro-translocation and degradation of ER membrane GPI-anchored mis-folded proteins. |
| S8 | ERADL | ***Kar2p,Pdi1p,Mnl1p,Yos9p,Ubc7p,Cue1p,Ubc7p,Cue1p,Ubx2p,Cdc48p,Ufd1p,Npl4p,Hrd3p,Hrd1p,Usa1p,Der1p ,Uba1p***  Png1p, 26sproteosome | The processes for detection, retro-translocation and degradation of ER luminal mis-folded proteins. |
| S9 | ERADM | ***Hrd1p,Ubc7p, Cue1p,Usa1p, Der1p,Ubx2p, Cdc48p, Ufd1p,Npl4p, Png1p***,26sproteosome | The processes for detection, retro-translocation and degradation of ER membrane mis-folded proteins. |
| S10 | COPII | ***Sar1p,Sec23p,Sec24p,Bet1p,Bos1p,Erv29p,Emp24p,Sec13p,Sec31p,Sec16p,Sed4p,Sec17p,Sec22p,Ypt1p,Uso1p,bug1p,Bet3p,Bet5p,Trs20p,Trs23p,Trs31p,Trs33p*** | Include all the mechanism needed for specific cargo selection from ER, vesiculation and fusion to the receptor membrane(Golgi) |
| S11 | COPI | ***Arf1p,Ret1p,Sec26p,Sec27p,Sec21p,Ret2p,Sec28p,Ret3p,Gea1p,Gea2p,Rer1p,ret2P,Gol3p,Erd2p,Rer1p,Ret2p,Cop1p,sec27p,sec21p,Bet1p*** | The vesiculation processes for retrieving the ER escaped resident proteins and maintaining the ER membrane balance. |
| S12 | Golgi processing | ***Anp1p,Mnn9p,Mnn10p,Mnn11p,Hoc1p,Kex1p,Kex2p,Ste13p,Kre2p,Mnn1p,Ktr1p,Ktr3p,Set13p*** | All the modification processes (Golgi glycosylation and peptide cleavage) |
| S13 | LDSV(high density secretory vesicle) | ***Arf1p,Sec3p,Sec5p,Sec6p,Sec8p,Sec10p,Sec15p,Exo70p,Exo84p,Sec4p,Chc1p,Clc1p*** | The transport of the cell membrane proteins from Golgi. |
| S14 | HDSV(high density secretory vesicle) | ***Arf1p, Pep12,Swa2p,Chc1p,Clc1p,Apl4p,Apl2p,Apm1p,Aps1p,Vps1p*** | The transport of the secreted and cell wall proteins from Golgi. |
| S15 | CPY pathway | ***Gga1p, Gga2p,Arf1p,Apl4p,Apl2p,Apm1p,Aps1p,Chc1p,Clc1p,Pep12p,Vps45p,Apl6p,Aps3p,Apm3p,Apl5p,Vam3p*** | Responsible for transporting vacuole soluble protein from Glogi. |
| S16 | ALP pathway(AP-3 complex) | *Apl6p, Aps3p, Apm3p,Apl5p,Vam3p,* | Responsible for transporting vacuole membrane protein from Glogi. |

**Supplementary Table S2.** D**efined subsystems and their involved protein components in the the yeast secretory machinery model.**

| complex | Subsystem | component |
| --- | --- | --- |
| **SRPC** | translocation from cytosol to ER | Srp14p, Srp21p,  Srp68p,  Srp72p,  Srp65p,  ScR1, Srp54p |
| **SRC** | Co-translocation from cytosol to ER | Srp101p, Srp102p |
| **SEC61C1** | Co-translocation translocation | Sec61p, Sbh1p Sss1p |
| **SEC61C2** | Post-translational translocation | Sbh1p,  Sss1p,  Ssh1p |
| **SPC** | translocation from cytosol to ER | Sec11p Spc1p Spc2p Spc3p |
| **SEC62C** | Post-translational translocation | Sec63p,  Sec62p,  Sec66p, Sec72p |
| **OSTC** | **Protein N-glycosylation** | Ost1p, Ost2p, Ost3p, Ost4p, Ost5p, Ost6p, Stt3p, Swp1p, Wbp1p |
| **HRD1-HRD3C** | ERADL | Hrd3p, hrd1p , Usa1p, Der1p, |
| **AAAC** | ERADL | Ubx2p, Cdc48, Ufd1p, Npl4p, |
| **Sec23-sec 24C** | COPII | Sec23p, sec 24p, |
| **TRAPPIC** | COPII | Bet3p, Bet5p, Trs20p, Trs23p, Trs31p,Trs33p |
| **AP3C** | ALP pathway(AP-3 complex) | Apl6p, Aps3p, Apm3p,  Apl5p,  Vam3p, |
| **AP1C** | CPY Pathway | Apl4p, Apl2p,Apm1p,Aps1p |
| **AP2C** |  | Apl3p, Apl1p ,Apm4p , Aps2p |
| **Sec13-sec31C** | COPII | Sec13p,  sec31p |
| **ClathrinC** | CPY Pathway | Chc1p,Clc1p |
| **GPIS1C** | GPI biosynthesis | Gpi1p, Gpi2p, Gpi3p,Gpi15p,Gpi19p,Eri11p |
| **Arf1p-GTP** | COPI | Arf1p,  Gea1p,  Gea2p, |
| **COPIC** | COPI | Ret1p, Sec26p, Sec27p, Sec21p, Ret2p,  Sec28p,  Ret3p |
| **CPYIC** | CPYI pathway | Pep12p,  Vps45p, |
| CPYIIC | CPYII pathway | Vps4p,Vps27p |

**Supplementary Table 3** the protein complexes used in the model with the protein involved in complex formation reactions.

**Supplementary Table 4.** The highly metabolic demanded proteins for yeast secretory pathway proteins.

| SGD ID | Protein Name | Description |
| --- | --- | --- |
| S000001956 | Cwp2p | Covalently linked cell wall mannoprotein, major constituent of the cell wall; plays a role in stabilizing the cell wall; involved in low pH resistance; precursor is GPI-anchored |
| S000003571 | Kar2p | ATPase involved in protein import into the ER, also acts as a chaperone to mediate protein folding in the ER and may play a role in ER export of soluble proteins; regulates the unfolded protein response via interaction with Ire1p |
| S000002440 | Mrh1p | Protein that localizes primarily to the plasma membrane, also found at the nuclear envelope; the authentic, non-tagged protein is detected in mitochondria in a phosphorylated state; has similarity to Hsp30p and Yro2p |
| S000004100 | Ccw12p | Cell wall mannoprotein with a role in maintenance of newly synthesized areas of cell wall; localizes to periphery of small buds, septum region of larger buds, and shmoo tip |
| S000000619 | Pmp1p | Small single-membrane span proteolipid that functions as a regulatory subunit of the plasma membrane H(+)-ATPase Pma1p, forms unique helix and positively charged cytoplasmic domain that is able to specifically segregate phosphatidylserines |
| S000003292 | Erg25p | C-4 methyl sterol oxidase, catalyzes the first of three steps required to remove two C-4 methyl groups from an intermediate in ergosterol biosynthesis; mutants accumulate the sterol intermediate 4,4-dimethylzymosterol |
| S000002103 | Pmp2p | Proteolipid associated with plasma membrane H(+)-ATPase (Pma1p); regulates plasma membrane H(+)-ATPase activity; nearly identical to PMP1 |
| S000004473 | Erv25p | Protein that forms a heterotrimeric complex with Erp1, Erp2p, and Emp24, member of the p24 family involved in endoplasmic reticulum to Golgi transport |
| S000001084 | Ncp1p | NADP-cytochrome P450 reductase; involved in ergosterol biosynthesis; associated and coordinately regulated with Erg11p |
| S000003514 | Bgl2p | Endo-beta-1,3-glucanase, major protein of the cell wall, involved in cell wall maintenance |
| S000004912 | Prc1p | Vacuolar carboxypeptidase Y (proteinase C; CPY), broad-specificity C-terminal exopeptidase involved in non-specific protein degradation in the vacuole; member of the serine carboxypeptidase family |
| S000006429 | Ccw14p | Covalently linked cell wall glycoprotein, present in the inner layer of the cell wall |
| S000000363 | Ifa38p | Microsomal beta-keto-reductase; contains oleate response element (ORE) sequence in the promoter region; mutants exhibit reduced VLCFA synthesis, accumulate high levels of dihydrosphingosine, phytosphingosine and medium-chain ceramides |
| S000000271 | Tip1p | Major cell wall mannoprotein with possible lipase activity; transcription is induced by heat- and cold-shock; member of the Srp1p/Tip1p family of serine-alanine-rich proteins |
| S000003421 | Crh1p | Chitin transglycosylase that functions in the transfer of chitin to beta(1-6) and beta(1-3) glucans in the cell wall; similar and functionally redundant to Utr2; localizes to sites of polarized growth; expression induced by cell wall stress |
| S000004594 | Pga3p | Putative cytochrome b5 reductase, localized to the plasma membrane; may be involved in regulation of lifespan; required for maturation of Gas1p and Pho8p, proposed to be involved in protein trafficking |
| S000003168 | Emp24p | Component of the p24 complex; binds to GPI anchor proteins and mediates their efficient transport from the ER to the Golgi; integral membrane protein that associates with endoplasmic reticulum-derived COPII-coated vesicles |
| S000000005 | Erp2p | Protein that forms a heterotrimeric complex with Erp1p, Emp24p, and Erv25p; member, along with Emp24p and Erv25p, of the p24 family involved in ER to Golgi transport and localized to COPII-coated vesicles |
| S000005010 | Sun4p | Cell wall protein related to glucanases, possibly involved in cell wall septation; member of the SUN family |
| S000002996 | Scw11p | Cell wall protein with similarity to glucanases; may play a role in conjugation during mating based on its regulation by Ste12p |

**Supplementary Table 5**.The top 20 most abundant proteins in yeast cell

| SGD ID | Protein Name | Description |
| --- | --- | --- |
| S000001956 | Cwp2p | Covalently linked cell wall mannoprotein, major constituent of the cell wall; plays a role in stabilizing the cell wall; involved in low pH resistance; precursor is GPI-anchored |
| S000002976 | Pma1p | Plasma membrane H+-ATPase, pumps protons out of the cell; major regulator of cytoplasmic pH and plasma membrane potential; P2-type ATPase; Hsp30p plays a role in Pma1p regulation; interactions with Std1p appear to propagate [GAR+] |
| S000001543 | Fba1p | Fructose 1,6-bisphosphate aldolase, required for glycolysis and gluconeogenesis; catalyzes conversion of fructose 1,6 bisphosphate to glyceraldehyde-3-P and dihydroxyacetone-P; locates to mitochondrial outer surface upon oxidative stress |
| S000004347 | Ilv5p | Bifunctional acetohydroxyacid reductoisomerase and mtDNA binding protein; involved in branched-chain amino acid biosynthesis and maintenance of wild-type mitochondrial DNA; found in mitochondrial nucleoids |
| S000004239 | Yef3p | Gamma subunit of translational elongation factor eEF1B, stimulates the binding of aminoacyl-tRNA (AA-tRNA) to ribosomes by releasing eEF1A (Tef1p/Tef2p) from the ribosomal complex; contains two ABC cassettes; binds and hydrolyzes ATP |
| S000004975 | Hhf2p | Histone H4, core histone protein required for chromatin assembly and chromosome function; one of two identical histone proteins (see also HHF1); contributes to telomeric silencing; N-terminal domain involved in maintaining genomic integrity |
| S000002790 | Rpp2Bp | Ribosomal protein P2 beta, a component of the ribosomal stalk, which is involved in the interaction between translational elongation factors and the ribosome; regulates the accumulation of P1 (Rpp1Ap and Rpp1Bp) in the cytoplasm |
| S000000213 | Hhf1p | Histone H4, core histone protein required for chromatin assembly and chromosome function; one of two identical histone proteins (see also HHF2); contributes to telomeric silencing; N-terminal domain involved in maintaining genomic integrity |
| S000003865 | Sod1p | Cytosolic copper-zinc superoxide dismutase; some mutations are analogous to those that cause ALS (amyotrophic lateral sclerosis) in humans |
| S000000933 | Rps26Bp | Protein component of the small (40S) ribosomal subunit; nearly identical to Rps26Ap and has similarity to rat S26 ribosomal protein |
| S000004124 | Pdc5p | Minor isoform of pyruvate decarboxylase, key enzyme in alcoholic fermentation, decarboxylates pyruvate to acetaldehyde, regulation is glucose- and ethanol-dependent, repressed by thiamine, involved in amino acid catabolism |
| S000006161 | Hsp82p | Hsp90 chaperone required for pheromone signaling and negative regulation of Hsf1p; docks with Tom70p for mitochondrial preprotein delivery; promotes telomerase DNA binding and nucleotide addition; interacts with Cns1p, Cpr6p, Cpr7p, Sti1p |
| S000000098 | Htb2p | Histone H2B, core histone protein required for chromatin assembly and chromosome function; nearly identical to HTB1; Rad6p-Bre1p-Lge1p mediated ubiquitination regulates transcriptional activation, meiotic DSB formation and H3 methylation |
| S000005399 | Rpp2Ap | Ribosomal protein P2 alpha, a component of the ribosomal stalk, which is involved in the interaction between translational elongation factors and the ribosome; regulates the accumulation of P1 (Rpp1Ap and Rpp1Bp) in the cytoplasm |
| S000004490 | Tsa1p | Thioredoxin peroxidase, acts as both a ribosome-associated and free cytoplasmic antioxidant; self-associates to form a high-molecular weight chaperone complex under oxidative stress; deletion results in mutator phenotype |
| S000003947 | Ssa2p | ATP binding protein involved in protein folding and vacuolar import of proteins; member of heat shock protein 70 (HSP70) family; associated with the chaperonin-containing T-complex; present in the cytoplasm, vacuolar membrane and cell wall; 98% identical with Ssa1p, but subtle differences between the two proteins provide functional specificity with respect to propagation of yeast [URE3] prions and vacuolar-mediated degradations of gluconeogenesis enzymes |
| S000006239 | Gln1p | Glutamine synthetase (GS), synthesizes glutamine from glutamate and ammonia; with Glt1p, forms the secondary pathway for glutamate biosynthesis from ammonia; expression regulated by nitrogen source and by amino acid limitation |
| S000003571 | Kar2p | ATPase involved in protein import into the ER, also acts as a chaperone to mediate protein folding in the ER and may play a role in ER export of soluble proteins; regulates the unfolded protein response via interaction with Ire1p |
| S000004592 | Pho84p | High-affinity inorganic phosphate (Pi) transporter and low-affinity manganese transporter; regulated by Pho4p and Spt7p; mutation confers resistance to arsenate; exit from the ER during maturation requires Pho86p; cells overexpressing Pho84p accumulate heavy metals but do not develop symptoms of metal toxicity |
| S000004722 | Asc1p | G-protein beta subunit and guanine nucleotide dissociation inhibitor for Gpa2p; ortholog of RACK1 that inhibits translation; core component of the small (40S) ribosomal subunit; represses Gcn4p in the absence of amino acid starvation |

# References

1. Johnson AE, van Waes MA: **The translocon: a dynamic gateway at the ER membrane.** *Annu Rev Cell Dev Biol* 1999, **15:**799-842.

2. Zimmermann R, Eyrisch S, Ahmad M, Helms V: **Protein translocation across the ER membrane.** *Biochimica et Biophysica Acta (BBA)-Biomembranes* 2010.

3. Walter P, Johnson AE: **Signal sequence recognition and protein targeting to the endoplasmic reticulum membrane.** *Annual review of cell biology* 1994, **10:**87-119.

4. Gilmore R, Walter P, Blobel G: **Protein translocation across the endoplasmic reticulum. II. Isolation and characterization of the signal recognition particle receptor.** *The Journal of Cell Biology* 1982, **95:**470.

5. Egea PF, Shan S, Napetschnig J, Savage DF, Walter P, Stroud RM: **Substrate twinning activates the signal recognition particle and its receptor.** *Nature* 2004, **427:**215-221.

6. Hansen W, Garcia PD, Walter P: **In vitro protein translocation across the yeast endoplasmic reticulum: ATP-dependent post-translational translocation of the prepro-[alpha]-factor.** *Cell* 1986, **45:**397-406.

7. Martoglio B, Dobberstein B: **Signal sequences: more than just greasy peptides.** *Trends in cell biology* 1998, **8:**410-415.

8. Gierasch LM: **Signal sequences.** *Biochemistry* 1989, **28:**923-930.

9. Plessis DJF, Nouwen N, Driessen AJM: **The Sec translocase.** *abbr) Biochim Biophys Acta*, **1808**.

10. Goldshmidt H, Sheiner L, Bütikofer P, Roditi I, Uliel S, Günzel M, Engstler M, Michaeli S: **Role of protein translocation pathways across the endoplasmic reticulum in Trypanosoma brucei.** *Journal of Biological Chemistry* 2008, **283:**32085.

11. Ciufo LF, Brown JD: **Nuclear export of yeast signal recognition particle lacking Srp54p by the Xpo1p/Crm1p NES-dependent pathway.** *Current Biology* 2000, **10:**1256-1264.

12. Ogg S, Poritz M, Walter P: **Signal recognition particle receptor is important for cell growth and protein secretion in Saccharomyces cerevisiae.** *Molecular biology of the cell* 1992, **3:**895.

13. Rothblatt JA, Deshaies RJ, Sanders SL, Daum G, Schekman R: **Multiple genes are required for proper insertion of secretory proteins into the endoplasmic reticulum in yeast.** *The Journal of Cell Biology* 1989, **109:**2641.

14. Ooi CE, Weiss J: **Bidirectional movement of a nascent polypeptide across microsomal membranes reveals requirements for vectorial translocation of proteins.** *Cell* 1992, **71:**87-96.

15. Klappa P, Freedman RB, Zimmermann R: **Protein disulphide isomerase and a lumenal cyclophilin-type peptidyl prolyl cis-trans isomerase are in transient contact with secretory proteins during late stages of translocation.** *European Journal of Biochemistry* 1995, **232:**755-764.

16. Mothes W, Heinrich SU, Graf R, Nilsson I, Von Heijne G, Brunner J, Rapoport TA: **Molecular mechanism of membrane protein integration into the endoplasmic reticulum.** *Cell* 1997, **89:**523-533.

17. Hartmann E, Rapoport TA, Lodish HF: **Predicting the orientation of eukaryotic membrane-spanning proteins.** *Proceedings of the National Academy of Sciences* 1989, **86:**5786.

18. White SH, von Heijne G: **How translocons select transmembrane helices.** *Annu Rev Biophys* 2008, **37:**23-42.

19. Anken E, Braakman I: **Versatility of the endoplasmic reticulum protein folding factory.** *Critical reviews in biochemistry and molecular biology* 2005, **40:**191-228.

20. Gavin AC, Aloy P, Grandi P, Krause R, Boesche M, Marzioch M, Rau C, Jensen LJ, Bastuck S, Dümpelfeld B: **Proteome survey reveals modularity of the yeast cell machinery.** *Nature* 2006, **440:**631-636.

21. Senger RS, Karim MN: **Variable Site‐Occupancy Classification of N‐Linked Glycosylation Using Artificial Neural Networks.** *Biotechnology progress* 2005, **21:**1653-1662.

22. Kasturi L, Chen H, Shakin-Eshleman SH: **Regulation of N-linked core glycosylation: use of a site-directed mutagenesis approach to identify Asn-Xaa-Ser/Thr sequons that are poor oligosaccharide acceptors.** *Biochemical Journal* 1997, **323:**415.

23. Mellquist J, Kasturi L, Spitalnik S, Shakin-Eshleman S: **The amino acid following an asn-X-Ser/Thr sequon is an important determinant of N-linked core glycosylation efficiency.** *Biochemistry* 1998, **37:**6833-6837.

24. Trimble R, Verostek M: **Glycoprotien Oligosaccharides Synthesis and Processing in Yeast.** *TRENDS IN GLYCOSCIENCE AND GLYCOTECHNOLOGY* 1995, **7:**1-34.

25. Schenk B, Fernandez F, Waechter CJ: **The ins (ide) and outs (ide) of dolichyl phosphate biosynthesis and recycling in the endoplasmic reticulum.** *Glycobiology* 2001, **11:**61R.

26. Orci L, Malhotra V, Amherdt M, Serafini T, Rothman JE: **Dissection of a single round of vesicular transport: sequential intermediates for intercisternal movement in the Golgi stack.** *Cell* 1989, **56:**357-368.

27. Bickel T, Lehle L, Schwarz M, Aebi M, Jakob CA: **Biosynthesis of Lipid-linked Oligosaccharides in Saccharomyces cerevisiae.** *Journal of Biological Chemistry* 2005, **280:**34500-34506.

28. Burda P, Aebi M: **The dolichol pathway of N-linked glycosylation.** *Biochimica et Biophysica Acta (BBA)-General Subjects* 1999, **1426:**239-257.

29. Lippincott-Schwartz J: **An evolving paradigm for the secretory pathway?** *Molecular biology of the cell* 2011, **22:**3929-3932.

30. McCracken AA, Brodsky JL: **Assembly of ER-associated protein degradation in vitro: dependence on cytosol, calnexin, and ATP.** *The Journal of Cell Biology* 1996, **132:**291.

31. Carvalho P, Goder V, Rapoport TA: **Distinct ubiquitin-ligase complexes define convergent pathways for the degradation of ER proteins.** *Cell* 2006, **126:**361-373.

32. Denic V, Quan EM, Weissman JS: **A luminal surveillance complex that selects misfolded glycoproteins for ER-associated degradation.** *Cell* 2006, **126:**349-359.

33. Gauss R, Jarosch E, Sommer T, Hirsch C: **A complex of Yos9p and the HRD ligase integrates endoplasmic reticulum quality control into the degradation machinery.** *Nature cell biology* 2006, **8:**849-854.

34. Lilley BN, Ploegh HL: **A membrane protein required for dislocation of misfolded proteins from the ER.** *Nature* 2004, **429:**834-840.

35. Varga K, Jurkuvenaite A, Wakefield J, Hong JS, Guimbellot JS, Venglarik CJ, Niraj A, Mazur M, Sorscher EJ, Collawn JF: **Efficient intracellular processing of the endogenous cystic fibrosis transmembrane conductance regulator in epithelial cell lines.** *Journal of Biological Chemistry* 2004, **279:**22578.

36. Ferreira T, Mason AB, Pypaert M, Allen KE, Slayman CW: **Quality control in the yeast secretory pathway.** *Journal of Biological Chemistry* 2002, **277:**21027-21040.

37. Margittai É, Sitia R: **Oxidative protein folding in the secretory pathway and redox signaling across compartments and cells.** *Traffic* 2011, **12:**1-8.

38. Stevens FJ, Argon Y: **Protein folding in the ER* 1.** In. Elsevier; 1999: 443-454.

39. Ming Y, Jingzhi L, Bingdong S: **Structural analysis of the Sil1-Bip complex reveals the mechanism for Sil1 to function as a nucleotide-exchange factor.** *Biochemical Journal* 2011, **438:**447-455.

40. Clerc S, Hirsch C, Oggier D, Deprez P, Jakob C, Sommer T, Aebi M: **Htm1 protein generates the N-glycan signal for glycoprotein degradation in the endoplasmic reticulum.** *The Journal of Cell Biology* 2009, **184:**159.

41. Vembar S, Brodsky J: **One step at a time: endoplasmic reticulum-associated degradation.** *Nature Reviews Molecular Cell Biology* 2008, **9:**944-957.

42. Stanley AM, Carvalho P, Rapoport T: **Recognition of an ERAD-L substrate analyzed by site-specific in vivo photocrosslinking.** *FEBS letters* 2011, **585:**1281-1286.

43. Xie W, Ng DTW: **ERAD substrate recognition in budding yeast.** *Seminars in Cell &amp; Developmental Biology* 2010, **21:**533-539.

44. Casagrande R, Stern P, Diehn M, Shamu C, Osario M, Zuniga M, Brown PO, Ploegh H: **Degradation of proteins from the ER of S-cerevisiae requires an intact unfolded protein response pathway.** *Molecular Cell* 2000, **5:**729-735.

45. Kohno K: **Stress-sensing mechanisms in the unfolded protein response: similarities and differences between yeast and mammals.** *Journal of biochemistry* 2010, **147:**27-33.

46. Patil C, Walter P: **Intracellular signaling from the endoplasmic reticulum to the nucleus: the unfolded protein response in yeast and mammals.** *Curr Opin Cell Biol* 2001, **13:**349-355.

47. Schroder M, Kaufman RJ: **ER stress and the unfolded protein response.** *Mutat Res* 2005, **569:**29-63.

48. Sato K, Nakano A: **Mechanisms of COPII vesicle formation and protein sorting.** *FEBS letters* 2007, **581:**2076-2082.

49. Mossessova E, Bickford LC, Goldberg J: **SNARE selectivity of the COPII coat.** *Cell* 2003, **114:**483-495.

50. Kuehn M, Herrmann J, Schekman R: **COPII–cargo interactions direct protein sorting into ER-derived transport vesicles.** *Nature* 1998, **391:**187-190.

51. Miller EA, Barlowe C: **Regulation of coat assembly—sorting things out at the ER.** *Current opinion in cell biology* 2010, **22:**447-453.

52. Zanetti G, Pahuja KB, Studer S, Shim S, Schekman R: **COPII and the regulation of protein sorting in mammals.** *Nature cell biology* 2012, **14:**20-28.

53. Rossanese OW, Soderholm J, Bevis BJ, Sears IB, O'Connor J, Williamson EK, Glick BS: **Golgi Structure Correlates with Transitional Endoplasmic Reticulum Organization in Pichia pastoris and Saccharomyces cerevisiae.** *The Journal of Cell Biology* 1999, **145:**69-81.

54. Nickel W, Rabouille C: **Mechanisms of regulated unconventional protein secretion.** *Nat Rev Mol Cell Biol* 2009, **10:**148-155.

55. Nickel W: **Pathways of unconventional protein secretion.** *Current opinion in biotechnology* 2010, **21:**621-626.

56. Nickel W: **The Unconventional Secretory Machinery of Fibroblast Growth Factor 2.** *Traffic* 2011.

57. Nishimura N, Balch WE: **A di-acidic signal required for selective export from the endoplasmic reticulum.** *Science* 1997, **277:**556.

58. Ma D, Zerangue N, Lin YF, Collins A, Yu M, Jan YN, Jan LY: **Role of ER export signals in controlling surface potassium channel numbers.** *Science* 2001, **291:**316.

59. Belden WJ, Barlowe C: **Role of Erv29p in collecting soluble secretory proteins into ER-derived transport vesicles.** *Science* 2001, **294:**1528.

60. Muñiz M, Nuoffer C, Hauri HP, Riezman H: **The Emp24 complex recruits a specific cargo molecule into endoplasmic reticulum–derived vesicles.** *The Journal of Cell Biology* 2000, **148:**925-930.

61. Rivier AS, Castillon GA, Michon L, Fukasawa M, Romanova‐Michaelides M, Jaensch N, Hanada K, Watanabe R: **Exit of GPI‐Anchored Proteins from the ER Differs in Yeast and Mammalian Cells.** *Traffic* 2010, **11:**1017-1033.

62. Miller E, Antonny B, Hamamoto S, Schekman R: **Cargo selection into COPII vesicles is driven by the Sec24p subunit.** *The EMBO Journal* 2002, **21:**6105-6113.

63. Spang A: **On vesicle formation and tethering in the ER-Golgi shuttle.** *Current opinion in cell biology* 2009, **21:**531-536.

64. Orci L, Ravazzola M, Meda P, Holcomb C, Moore HP, Hicke L, Schekman R: **Mammalian Sec23p homologue is restricted to the endoplasmic reticulum transitional cytoplasm.** *Proceedings of the National Academy of Sciences* 1991, **88:**8611.

65. Castillon GA, Aguilera-Romero A, Manzano-Lopez J, Epstein S, Kajiwara K, Funato K, Watanabe R, Riezman H, Muñiz M: **The yeast p24 complex regulates GPI-anchored protein transport and quality control by monitoring anchor remodeling.** *Molecular biology of the cell* 2011, **22:**2924-2936.

66. Anelli T, Sitia R: **Protein quality control in the early secretory pathway.** *The EMBO Journal* 2008, **27:**315-327.

67. Okuda-Shimizu Y, Shen Y, Hendershot L: **Protein Quality Control in the Endoplasmic Reticulum.** *Regulation of Organelle and Cell Compartment Signaling: Cell Signaling Collection* 2011**:**383.

68. Stagg SM, LaPointe P, Razvi A, Gurkan C, Potter CS, Carragher B, Balch WE: **Structural basis for cargo regulation of COPII coat assembly.** *Cell* 2008, **134:**474-484.

69. Scidmore MA, Okamura HH, Rose MD: **Genetic interactions between KAR2 and SEC63, encoding eukaryotic homologues of DnaK and DnaJ in the endoplasmic reticulum.** *Molecular biology of the cell* 1993, **4:**1145.

70. Schlenstedt G, Harris S, Risse B, Lill R, Silver PA: **A yeast DnaJ homologue, Scj1p, can function in the endoplasmic reticulum with BiP/Kar2p via a conserved domain that specifies interactions with Hsp70s.** *The Journal of Cell Biology* 1995, **129:**979-988.

71. Normington K, Kohno K, Kozutsumi Y, Gething MJ, Sambrook J: **S. cerevisiae encodes an essential protein homologous in sequence and function to mammalian BiP.** *Cell* 1989, **57:**1223-1236.

72. Zanetti G, Pahuja KB, Studer S, Shim S, Schekman R: **COPII and the regulation of protein sorting in mammals.** *Nature cell biology* 2012, **14:**221.

73. Watson P, Forster R, Palmer KJ, Pepperkok R, Stephens DJ: **Coupling of ER exit to microtubules through direct interaction of COPII with dynactin.** *Nature cell biology* 2004, **7:**48-55.

74. Cosson P, Letourneur F: **Coatomer (COPI)-coated vesicles: role in intracellular transport and protein sorting.** *Current opinion in cell biology* 1997, **9:**484-487.

75. Nakano A: **Yeast Golgi apparatus--dynamics and sorting.** *Cell Mol Life Sci* 2004, **61:**186-191.

76. Herscovics A: **Processing glycosidases of Saccharomyces cerevisiae.** *Biochim Biophys Acta* 1999, **1426:**275-285.

77. Cooper A, Bussey H: **Characterization of the yeast KEX1 gene product: a carboxypeptidase involved in processing secreted precursor proteins.** *Mol Cell Biol* 1989, **9:**2706-2714.

78. Rockwell NC, Krysan DJ, Komiyama T, Fuller RS: **Precursor processing by kex2/furin proteases.** *Chem Rev* 2002, **102:**4525-4548.

79. Julius D, Blair L, Brake A, Sprague G, Thorner J: **Yeast alpha factor is processed from a larger precursor polypeptide: the essential role of a membrane-bound dipeptidyl aminopeptidase.** *Cell* 1983, **32:**839-852.

80. Bard F, Malhotra V: **The formation of TGN-to-plasma-membrane transport carriers.** *Annu Rev Cell Dev Biol* 2006, **22:**439-455.

81. Bonifacino JS, Glick BS: **The mechanisms of vesicle budding and fusion.** *Cell* 2004, **116:**153-166.

82. Gurunathan S, David D, Gerst JE: **Dynamin and clathrin are required for the biogenesis of a distinct class of secretory vesicles in yeast.** *EMBO J* 2002, **21:**602-614.

83. Nakatsu F, Ohno H: **Adaptor protein complexes as the key regulators of protein sorting in the post-Golgi network.** *Cell Struct Funct* 2003, **28:**419-429.

84. Bowers K, Stevens TH: **Protein transport from the late Golgi to the vacuole in the yeast Saccharomyces cerevisiae.** *Biochimica et Biophysica Acta (BBA)-Molecular Cell Research* 2005, **1744:**438-454.
